# Tetrasubstituted imidazoles as incognito Toll-like receptor 8 a(nta)gonists

**DOI:** 10.1038/s41467-021-24536-4

**Published:** 2021-07-16

**Authors:** Yi Yang, Adam Csakai, Shuangshuang Jiang, Christina Smith, Hiromi Tanji, Jian Huang, Torey Jones, Kentaro Sakaniwa, Lindsey Broadwell, Chengrui Shi, Subada Soti, Umeharu Ohto, Yaohui Fang, Shu Shen, Fei Deng, Toshiyuki Shimizu, Hang Yin

**Affiliations:** 1 School of Pharmaceutical Sciences, Key Laboratory of Bioorganic Phosphorous Chemistry and Chemical Biology (Ministry of Education), Department of Chemistry, Beijing Advanced Innovation Center for Structural Biology, Tsinghua-Peking Joint Center for Life Sciences, Tsinghua University, Beijing, China; 2 Department of Chemistry & Biochemistry and the BioFrontiers Institute, University of Colorado Boulder, Boulder, CO USA; 3 Graduate School of Pharmaceutical Sciences, The University of Tokyo, Tokyo, Japan; 4 Wuhan Institute of Virology, Chinese Academy of Sciences, Wuhan, China

**Keywords:** Medicinal chemistry, Toll-like receptors, Mechanism of action, Small molecules, Drug discovery and development

## Abstract

Small-molecule modulators of TLR8 have drawn much interests as it plays pivotal roles in the innate immune response to single-stranded RNAs (ssRNAs) derived from viruses. However, their clinical uses are limited because they can invoke an uncontrolled, global inflammatory response. The efforts described herein culminate in the fortuitous discovery of a tetrasubstituted imidazole **CU-CPD107** which inhibits R848-induced TLR8 signaling. In stark contrast, **CU-CPD107** shows unexpected synergistic agonist activities in the presence of ssRNA, while **CU-CPD107** alone is unable to influence TLR8 signaling. **CU-CPD107**’s unique, dichotomous behavior sheds light on a way to approach TLR agonists. **CU-CPD107** offers the opportunity to avoid the undesired, global inflammation side effects that have rendered imidazoquinolines clinically irrelevant, providing an insight for the development of antiviral drugs.

## Abstract (web summary)

Toll-like receptor 8 (TLR8) plays essential roles in the innate immune response to viral single-stranded RNA (ssRNA), so small molecule modulators of TLR8 are of interest, however adverse effects limit their use. Here, the authors report a tetrasubstituted imidazole **CU-CPD107** with dichotomous behaviour, which inhibits R848-induced TLR8 signaling, but shows synergistic activity in the presence of ssRNA, making it a potential antiviral agent.

## Introduction

Toll-like receptors (TLRs) are highly conserved and crucial components of the innate immune system^1,2^. TLR3, 7, 8, and 9 located on endosomal membranes can recognize nucleic acids from viruses or bacteria, triggering potent antiviral and antitumor immune responses. Particularly, human TLR8 mediates antiviral immunity by recognizing ssRNA viruses^3–5^. While TLR1, 2, 4, 5, and 6 expressed on the plasma membrane can recognize extracellular stimuli. Once detected, these signals trigger an inflammatory response through transcription factors, such as NF-κB and interferon regulatory factors (IRFs). Activation of these transcription factors results in the production of pro-inflammatory cytokines and type I interferons, which are crucial in the activation of adaptive immunity^6^. The immune response is paramount to the host organism’s ability to defend itself against invading pathogens, and underactivation of TLRs leaves the host vulnerable to widespread infection^7^. However, this immune response can be a double-edged sword. Aberrant activation of TLRs results in an overabundance of inflammatory factors that may lead to several autoimmune disorders and septic shock or even killing the host^8,9^.

Recent work has shown that TLR8 can uniquely induce a robust inflammatory response in newborns, who are particularly vulnerable to infection^10^. TLR agonists are promising vaccine adjuvants and antiviral agents, as TLR activation can control the induction, extent, and duration of an adaptive immune response through the production of cytokines^6^. For example, monophosphoryl lipid A activates TLR4 which is one of the FDA-approved adjuvants^11^. Despite the lack of formal approval, it has been observed that many vaccines contain TLR agonists which act as adjuvants, including the Influvac and yellow fever vaccines^12–14^. TLR8 is a particularly enticing target due to its expression on myeloid dendritic cells (mDCs) and the proven capacity to activate the Type 1 helper T cells (Th1) cell-mediated immune response^6^. Particularly, TLR8 also plays an important role in the antiviral immune response through recognition of viral RNA in endocytic compartments^15^.

Enhancing the body’s innate immunity through TLR7 or TLR8 has been proved to be an effective antivirus strategy^3,5,16^. R848, a TLR7 and TLR8 dual agonist^17^, also known as Resiquimod, is an FDA-approved drug formulated as a topical gel for the treatment of skin lesions caused by the herpes simplex virus and cutaneous T-cell lymphoma. Naturally, these small molecules have been tested as potential vaccine adjuvants and antiviral reagents, but few are suited for pharmacological development^6^ because they can cause nonspecific, global inflammation, and poor immune recruitment adjacent to the injection. Despite these shortcomings, imidazoquinolines are a prudent starting point in the search for TLR8-selective modulators, especially with their strong affinity and known binding mode^18^. However, designing TLR8 selective modulators has been a challenge due to the high degree of homology with TLR7. While few specific TLR8 antagonists are present in the literature^19–21^, imidazoquinoline-based agonists have been widely used for the study of TLR8 and TLR7^17^. Specifically, these compounds bind to TLR8 via utilizing hydrophobic interactions at the 2-position R-group, and three key hydrogen bonds are facilitated by Asp 543* and Thr 574*. Pi-stacking also occurs between Phe 405 and the quinoline ring of the scaffold, respectively (Supplementary Fig. 1).

In this work, we showed a series of rationally designed TLR8-specific small molecules with unique synergistic activities in the presence of ssRNA, but inactive without the aid of ssRNA, making them potential antiviral reagents with characteristics distinct from imidazoquinolines. By considering the structural differences between the homologous TLR7 and TLR8, we identified a small-molecule scaffold that is tunable in both potency and specificity. Finally, we further assayed the lead compound **CU-CPD107** in both cellular and biophysical systems to ascertain its mechanism and function.

## Results

### Design and rationale

**Fig. 1:**
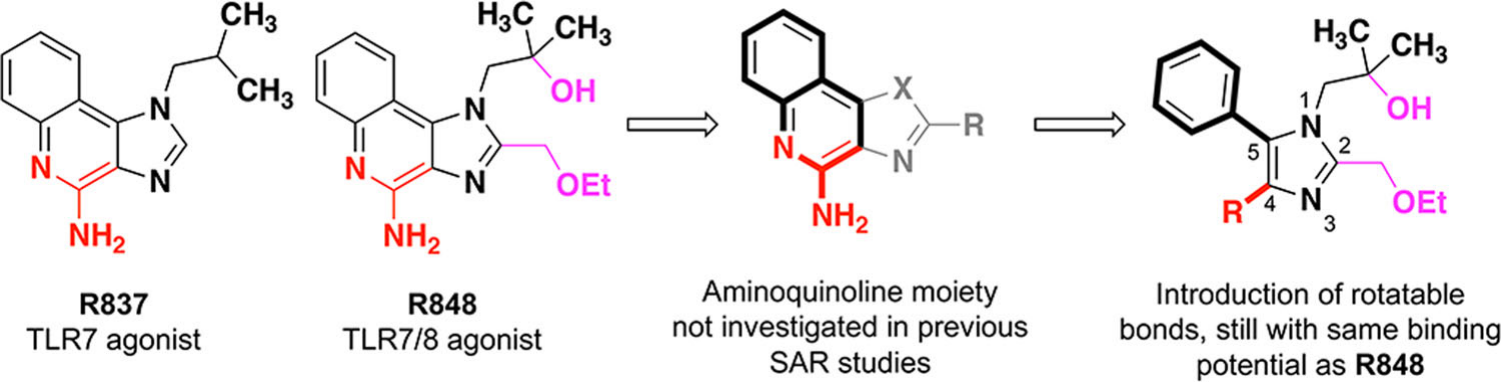
Design and rationale of tetrasubstituted imidazole derivatives. The structure of R837 (a selective TLR7 agonist) and R848 (a nonselective TLR7 and TLR8 agonist) are shown. Previous SAR efforts resulted in R848 and R837 mainly focused on the 5-membered azole ring and its substituents. In this work, we broke apart the aminoquinoline ring to introduce more rotatable bonds but maintained the substituents that originally provided TLR8 activity.

The original discovery of imidazoquinoline agonists included many selective TLR7 agonists^22–26^ and nonselective TLR7/8 agonists^3,22,23,26^, but few TLR8-selective agonists^27^. Consistent across these molecules is a fused 2-aminopyridine moiety, and most of structure–activity relationship (SAR) studies involved modifications on the remaining five-membered ring of the scaffold. From these studies, we can see that a selective TLR7 agonist R837 can be modified to gain TLR8 activity via the incorporation of a hydroxyl group and an ethoxymethyl chain, compared with R848 (Fig. 1). If TLR8 selectivity was to be obtained, we assumed it necessary to maintain these two additions. Since the original imidazoquinoline synthesis yielded little compounds lacking TLR7 activity, we thought it best to seek TLR8 selectivity via SAR studies of the little-explored 2-aminoimdazole moiety which had already produced a TLR8-specific agonist and a pan-TLR inhibitor^27^.

In our opinion, the most interesting approach for this SAR study is to break apart the fused ring system of the quinoline ring to give a tetrasubstituted imidazole (Fig. 1). This design may most probably maintain the potential for all the previously discovered imidazoquinoline binding interactions to occur but also allows for additional flexibility from the newly rotatable bonds at the 4-position and 5-position of the imidazole ring. A wide range of substitutions could be installed at the 4-position to provide a small library of compounds. We envisioned a variety of carboxylate derivatives as suitable bioisosteres for the 2-aminoquinoline moiety, and also found interest in functional groups that were unlikely to reproduce 2-aminoquinoline behavior, such as simple aliphatics and halogens. We believe that this strategy would open the possibility for the discovery of both agonists and antagonists since the proposed molecules could theoretically fit the same imidazoquinoline binding site, but potentially in a different configuration.

### Synthesis of imidazoquinoline derivatives

**Fig. 2:**
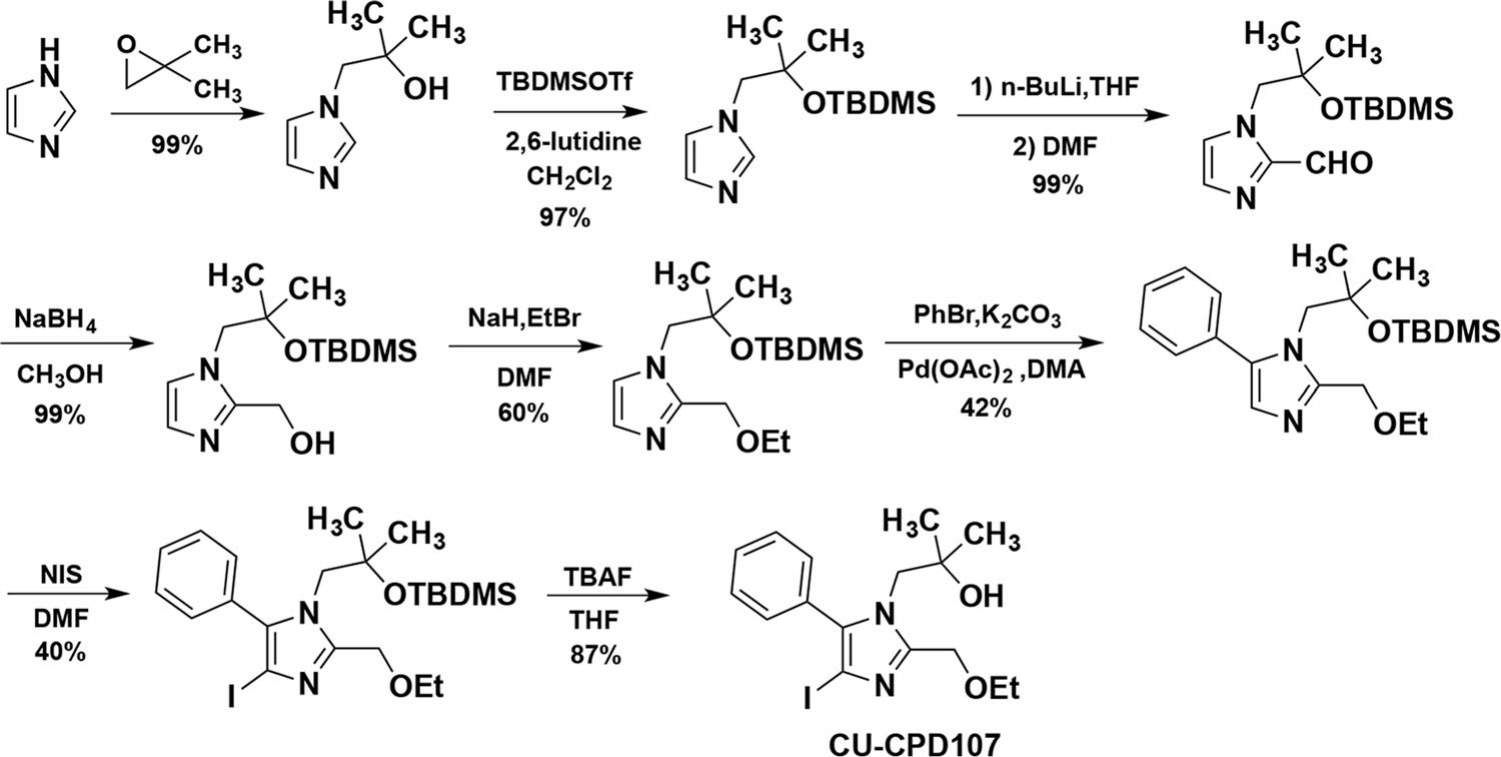
Representative synthetic route of the imidazoquinoline derivatives, taking CU-CPD107 as an example. **CU-CPD107** was synthesized in eight steps.

The biggest challenge in the synthesis of a tetrasubstituted imidazole scaffold is the regioselective functionalization of positions 1, 2, 4, and 5. Given our previously described target design, it was also important for us to have 4-position functionalized with a group that would be inert to functionalization chemistry at the other imidazole positions, and could then be easily modified to give rise to several other 4-position functional groups. The latter approach was employed in a synthesis, all the compounds in Table 1 were synthesized according to Supplementary Information. Taking **CU-CPD107** as an example (Fig. 2), it was started from the alkylation of imidazole with isobutylene oxide, as previously reported^28^. The newly formed tertiary alcohol required a protecting group that would last for the duration of the remaining synthesis, only to be removed in the last step. For this task, we chose to convert the alcohol into the *tert*-butyldimethylsilyl (TBDMS) ether with TBDMS triflate and 2,6-lutidine. Next, we would take advantage of the acidity at the 2-position of the imidazole by *n*-butyl lithium, and quenching with *N*,*N*-dimethylformamide (DMF) to give a 2-formylimidazole. Without purification, we carried out aldehyde reduction, followed by Williamson ether synthesis which successfully added the ethoxymethyl chain at 2-position. Then, we took advantage of the superior nucleophilicity of the 4-position and treated the imidazole with *N*-iodosuccinimide (NIS) to give the 4-iodo intermediate. With the neighboring iodine atom installed, a Suzuki coupling was carried out and finally removing protective groups with tetrabutylammonium fluoride (TBAF) in tetrahydrofuran (THF) to give **CU-CPD107**.

**Table 1:** Analysis of the structure–activity relationship of the tetrasubstituted imidazole analogs.


Compounds	R^1^	R^2^	R^3^	R^4^	R^5^	TLR7 Inhibition^[a]^	TLR8 Inhibition^[a]^
**1a**	–CN	–OH	–H	–H	–H	1.0 ± 0.2	1.1 ± 0.2
**1b**		–OH	–H	–H	–H	0.8 ± 0.1	1.0 ± 0.2
**1c**	–CONH_2_	–OH	–H	–H	–H	1.0 ± 0.1	0.7 ± 0.1
**1d**	–CHO	–OH	–H	–H	–H	1.2 ± 0.2	1.2 ± 0.2
**1e**	–COOCH_3_	–OH	–H	–H	–H	0.9 ± 0.2	0.9 ± 0.2
**1f**	–CH_2_OH	–OH	–H	–H	–H	0.9 ± 0.2	1.0 ± 0.2
**1g**	–CH_2_OCH_3_	–OH	–H	–H	–H	0.9 ± 0.2	1.0 ± 0.3
**1h**	–CH_3_	–OH	–H	–H	–H	0.9 ± 0.1	0.8 ± 0.2
**1i**	–CH_2_F	–OH	–H	–H	–H	0.8 ± 0.2	1.0 ± 0.2
**1j**	–C(CH_3_)_2_OH	–OH	–H	–H	–H	0.8 ± 0.1	0.9 ± 0.1
**1k**	–CH(CH_3_)_2_	–OH	–H	–H	–H	0.7 ± 0.1	0.9 ± 0.2
**1l**	–Ph	–OH	–H	–H	–H	0.8 ± 0.1	0.6 ± 0.0
**1m**	–H	–OH	–H	–H	–H	1.0 ± 0.1	1.1 ± 0.3
**1n**	–Cl	–OH	–H	–H	–H	1.1 ± 0.2	0.6 ± 0.1
**1o**	–Br	–OH	–H	–H	–H	0.8 ± 0.2	0.3 ± 0.2
**1p (CU-CPD107)**	–I	–OH	–H	–H	–H	1.0 ± 0.2	0.1 ± 0.1
**1q**	–COOH	–OH	–H	–H	–H	0.9 ± 0.2	0.9 ± 0.2
**2a**	–H	–H	–H	–H	–H	0.6 ± 0.2	0.8 ± 0.1
**2b**	–Cl	–H	–H	–H	–H	0.6 ± 0.2	0.5 ± 0.2
**2c**	–Br	–H	–H	–H	–H	0.1 ± 0.1	0.0 ± 0.1
**2d**	–I	–H	–H	–H	–H	0.1 ± 0.2	0.0 ± 0.0
**3a**	–I	–OH	–H	–H	–Ph	0.4 ± 0.0	0.3 ± 0.1
**3b**	–I	–OH	–H	–Ph	–H	0.7 ± 0.1	0.1 ± 0.1
**3c**	–I	–OH	–CH_3_	–H	–H	1.0 ± 0.0	0.9 ± 0.1
**3d**	–I	–OH	–H	–CH_3_	–H	1.0 ± 0.1	0.8 ± 0.1
**3e**	–I	–OH	–H	–H	–CH_3_	1.0 ± 0.0	0.9 ± 0.1

^[a]^The values reported represent the fraction of signaling observed using SEAP assay in HEK-Blue hTLR7 and hTLR8 cells compared to uninhibited R848 signaling, which was normalized to 1.0. Data are representative of the average and standard deviation of at least three independent experiments.

### Engineering tunable selectivity and potency

**Fig. 3:**
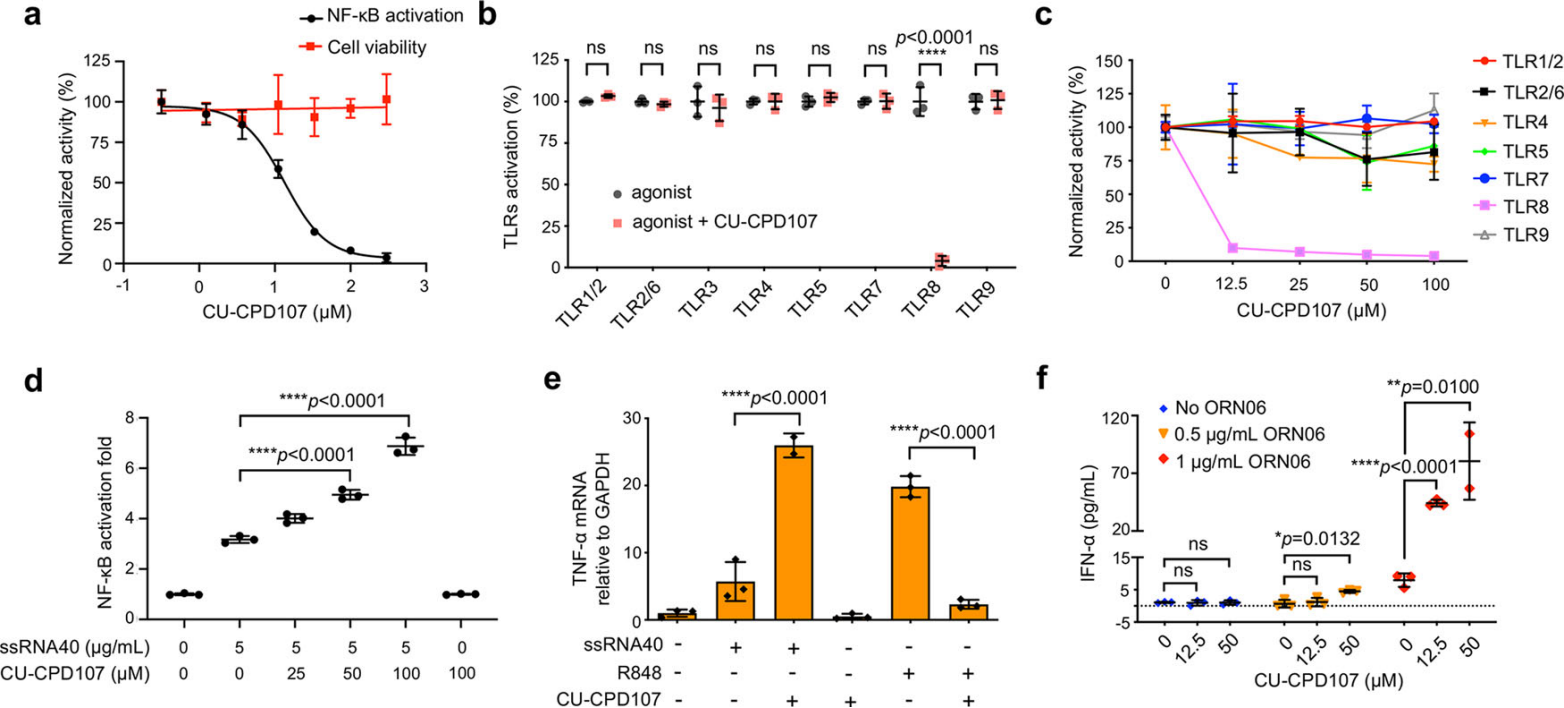
Dichotomous behavior of CU-CPD107 in modulating the TLR8 signaling pathway. **a** The dose–response inhibition curve of 2.9 µM R848-induced signaling in HEK-Blue hTLR8 cells is shown. Inhibition is clearly observed in HEK-Blue hTLR8 cells (IC_50_ = 13.7 ± 1.1 µM) with no obvious toxicity up to 300 μM. Data are mean ± s.d.; *n* = 3 independent experiments. **b** Specificity test for **CU-CPD107** (100 µM) using TLR-specific ligands to selectively activate the respective TLRs in HEK-Blue hTLRs cells for 24 h. Data are mean ± s.d.; *n* = 3 independent experiments. **c** Specificity test of **CU-CPD107** in PBMCs. Except for TLR8, **CU-CPD107** alone did not inhibit other TLR pathways. Data are normalized to **CU-CPD107** untreated cells. Data are mean ± s.d.; *n* = 3 independent experiments with independent blood donors. **d** **CU-CPD107** could also synergistically activate TLR8 signaling in the presence of ssRNA40 (5 μg/mL) with a dose-dependent manner, while **CU-CPD107** alone was unable to induce any activation. Signals are normalized to the untreated cells. Data are mean ± s.d.; *n* = 3 independent experiments. **e** **CU-CPD107** could synergistically upregulate the mRNA levels of TNF-α in the presence of ssRNA40 (5 μg/mL), while it could inhibit R848-induced activation and itself had no effect in HEK-Blue hTLR8 cells. Data are mean ± s.d.; the data shown are representative of three biologically independent experiments. **f** The synergistic effect of **CU-CPD107** in human PBMCs stimulated with ORN06. The production of IFN-α was measured by ELISA. Data are mean ± s.d.; the data shown are representative of three samples of independent blood donors examined over three biologically independent experiments. A one-way analysis of variance with Bonferroni’s multiple comparisons test for multiple comparisons was used for statistical analysis. Statistical significance of the data is indicated as follows: *P < 0.05, **P < 0.01, ***P < 0.001, ****P < 0.0001; ns not significant. Source data of Fig. 3 are provided as a Source data file.

With the rationale and synthetic routes above, we synthesized a series of tetrasubstituted imidazole derivatives (Table 1) and carried out SAR studies. With the first round of synthesis completed, all compounds were tested for their activity in HEK-Blue hTLR8 or hTLR7 cells activated by 100 µM R848 using SEAP assay (Table 1). We did not observe agonism in either cell line. To our surprise, the carboxylate derivatives (**1b**, **1c**, **1d**, **1e**, **and 1q** in Table 1) and aliphatic analogs (**1a**, **1f**, **1g**, **1h**, **1i**, **1j**, **1k**, and **1m** in Table 1) had little to no effect on R848-induced signaling at 100 μM, despite having such structural similarities to the imidazoquinolines. However, there was a clear inhibitory trend observed within the halogenated analogs (**1m**, **1n,1o**, and **CU-CPD107** in Table 1), favoring the larger, more polarizable iodo analog (**CU-CPD107**). To our delight, using **CU-CPD107** we observed significant inhibition of R848-induced signaling in HEK-Blue hTLR8 cells with an IC_50_ value of 13.7 ± 1.1 µM and no obvious toxicity up to 300 µM as detected by a WST1 assay (Fig. 3a) but had no effect on TLR7-mediated signaling. Intrigued by this newfound selectivity, we pursued more molecules bearing halogens at the 4-position of the imidazole. We thought an interesting hybrid R848/R837/**CU-CPD107** molecule could be made by removing the hydroxyl group that is common to R848 and **CU-CPD107**, but still maintaining the ethoxymethyl chain. Larger halogen substitutions at the 4-position decreased TLR7 and TLR8 signaling, however, this was due to increased toxicity of the molecules (**2a**, **2b**, **2c**, and **2d** in Table 1 and Supplementary Table 1).

One challenge in developing modulators to target TLRs is to engineer specificity and potency. There are at least 13 homologous TLRs present in mice and 10 in humans^29^. We, therefore, tested **CU-CPD107** against a panel of homologous hTLRs, including TLR1/TLR2, TLR2/TLR6, TLR3, TLR4, TLR5, TLR7, and TLR9 using TLR-specific ligands to selectively activate the particular TLR-overexpressing cells and human peripheral blood mononuclear cells (PBMCs). We found that **CU-CPD107** only inhibited synthesized small-molecule agonist-induced TLR8 signaling without affecting other TLRs, showing its high selectivity in intact cells (Fig. 3b, c).

### CU-CPD107 unexpectedly converts to an agonist in the presence of ssRNA

Upon the completion of the second round of optimization, **CU-CPD107** was identified as the most promising molecule among the entire series. While R848 is widely used to activate TLR7 and TLR8, it is not a natural ligand that has no biological relevance. So, it is important to test **CU-CPD107** in the presence of ssRNA ligands which better emulate a viral infection. ORN06 and ssRNA40 are two commercially available GU-rich ssRNA known to be effective agonists of mouse TLR7 and human TLR8. Much to our surprise, we observed a remarkable reversal of activity. Rather than inhibiting ssRNA-induced TLR8 signaling which would be consistent with R848-induced signaling, we observed that **CU-CPD107** behaved an agonist in the presence of ssRNA (Fig. 3d) in HEK-Blue hTLR8 cells, resulting in a fivefold activation at 100 μΜ by SEAP assay relative to the control. Crucially, **CU-CPD107** displayed no agonistic potential in the absence of ssRNA.

Next, we used a secondary cellular assay to confirm this unique, dichotomous behavior of **CU-CPD107**. TLR8 activation or inhibition can influence the downstream signaling transduction resulting in increased or decreased production of the cytokines^4^. We investigated the effects of **CU-CPD107** on the mRNA level of several downstream cytokines by reverse transcriptase quantitative PCR (RT-qPCR) in HEK-Blue hTLR8 cells. As shown in Fig. 3e and Supplementary Fig. 2, treatment with 100 μM **CU-CPD107** nearly abolished the elevation of IL-8, IFN-β, TNF-α, IL-1β, and IL-6 mRNA levels induced by 22.8 µM R848. By contrast, **CU-CPD107** synergistically increased IFN-β, TNF-α, IL-1β, IL-6, and IL-8 mRNA expression levels in the presence of 5 μg/ml ssRNA40, whereas **CU-CPD107** alone did not. It is important to note that ssRNA40 is a phosphorothioate (PS) modified RNA. To rule out the interference effects of PS, we further used ORN06 which is a native viral RNA sequence to confirm its synergistic activities with uridine as a positive control (Supplementary Fig. 3). The same behavior of **CU-CPD107** was also observed in human PBMCs by co-treating cells with TL8-506 or ORN06 (Supplementary Fig. 4). These phenomena were further confirmed the dichotomous behavior of **CU-CPD107** and exactly in agreement with the results in the SEAP assay.

Next, we detected the production of downstream cytokines in human PBMCs. To test the synergistic effect of **CU-CPD107** in the presence of ssRNA in PBMCs, the production of IFN-α and TNF-α was measured by an enzyme-linked immunosorbent assay (ELISA). We found that the production of IFN-α was strongly and synergistically upregulated in PBMCs when co-treated with ORN06 and **CU-CPD107** (Fig. 3f), which was in good agreement with the results shown in HEK-Blue hTLR8 cells. Besides, TNF-α could also be induced by **CU-CPD107** in the presence of ORN06 (Supplementary Fig. 5). Furthermore, **CU-CPD107** showed negligible cytotoxicity in PBMCs up to 100 μM (Supplementary Fig. 6). To sum up, **CU-CPD107**, different from the conventional agonists, only activated TLR8-mediated signaling with the aid of ssRNA, thus may avoid invoking an uncontrolled, global inflammatory response.

Based on the promising preliminary results, we also attempted to explore the structure of **CU-CPD107** to further understand its binding modes and structural constraints. We designed a series of analogs to extend the SAR studies around the benzene ring (**3a**, **3b**, **3c**, **3d**, and **3e** in Table 1). Compounds **3a** and **3b** showed moderate inhibitory effects on both TLR7 and TLR8 signaling. Compounds **3c**, **3d**, and **3e** showed no inhibitory effects on both TLR7 and TLR8 signaling but enhanced TLR8 signaling to more than fourfold with the aid of ssRNA compared to the control (Supplementary Fig. 7).

### *Kd* determination

In order to investigate the binding affinity of **CU-CPD107**, a series of isothermal titration calorimetry (ITC) experiments were performed with the isolated ectodomain of human TLR8 (Supplementary Fig. 8). First, **CU-CPD107** (100 µM) alone was titrated into purified TLR8 (10 µM), with no detectable change in heat observed (Supplementary Fig. 8a); R848 (100 µM) alone was titrated into TLR8 (10 µM) with an observed *K*_*d*_ of 35.7 nM (Supplementary Fig. 8b). For comparison, R848 (100 µM) was titrated into TLR8 protein (10 µM) premixed with **CU-CPD107** (100 µM), with no heat change observed (Supplementary Fig. 8c). These results indicated that **CU-CPD107** hindered R848 binding to TLR8, in agreement with the inhibition observed previously. Next, ORN06 (200 μM) was titrated to TLR8 (20 μM) with a *K*_*d*_ = 0.9 μM (Supplementary Fig. 8d). **CU-CPD107** (100 µM) was titrated into TLR8 protein (10 µM) in the presence of ORN06 (20 µM), showing an observed *K*_*d*_ of 146 nM (Supplementary Fig. 8e). Uridine (1 mM) was also titrated to TLR8 (20 μM) with a *K*_*d*_ = 12 μM (Supplementary Fig. 8f). While the *K*_*d*_ value for uridine in the presence of the oligonucleotide was 2.7 μM (Supplementary Fig. 8g) similar to the previous data^30^ which was about 18-fold weaker than **CU-CPD107**. Finally, **CU-CPD107** (100 µM) was titrated to ORN06 (40 µM) which did not show significant heat change (Supplementary Fig. 8h). Considering these results above, the heat change in Supplementary Fig. 8e would be derived from **CU-CPD107** binding to TLR8, but not the binding to ssRNA. This suggested that ssRNA greatly enhanced **CU-CPD107** binding to TLR8 in a similar manner as uridine did. Taken together, these results demonstrated that **CU-CPD107** differentially affects R848 and ssRNA ligands binding to TLR8.

### Dichotomous mechanism of CU-CPD107

**Fig. 4:**
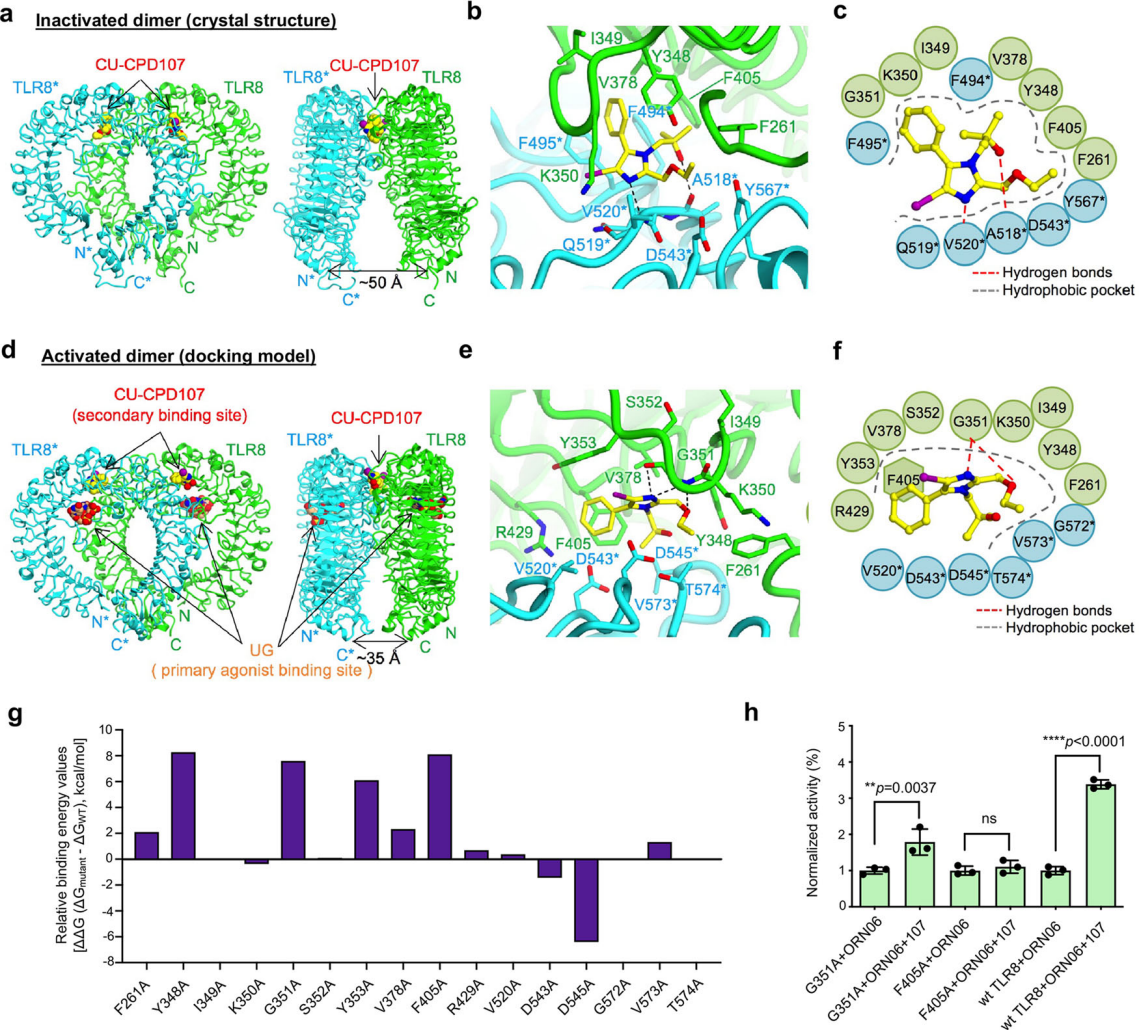
Unexpected molecular mechanism of CU-CPD107. **a** Crystal structure of the TLR8/**CU-CPD107** complex (PDB: 7CRF). Front (left) and side (right) views of the complex. TLR8 and its dimerization partner, TLR8*, are colored green and cyan, respectively. The ligand molecules are illustrated by space-filling representations. The C, O, and N atoms of the ligands are colored yellow, red, and blue, respectively. **b** and **c** are the close-up views and schematic representation of interactions between **CU-CPD107** and the TLR8 protein, respectively. The hydrophobic pocket and hydrogen bonds are shown as dashed gray arcs and dashed red lines, respectively. **d** Computational molecular modeling of **CU-CPD107** docked to TLR8/ssRNA40 (PDB: 4R08) suggesting that the dimeric interface is the most likely target region for **CU-CPD107**. **e** and **f** are the close-up views and schematic representation of interactions between the **CU-CPD107** with TLR8/ssRNA40 complex. **g** In silico alanine scanning of the residues within the **CU-CPD107**-binding site. The relative binding energy change (∆∆G) of each mutant over the wild type was calculated using the Prime-MM/GBSA method. **h** Activities of **CU-CPD107** in the mutants and wild-type hTLR8, stimulated by 5 μg/mL ORN06. The NF-κB induction fold of the mutants and wild-type TLR8 were analyzed by a SEAP assay using HEK-Blue Null1 cells. Data are mean ± s.d.; *n* = 3 independent experiments. A one-way analysis of variance with Bonferroni’s multiple comparisons test for multiple comparisons was used for statistical analysis. Statistical significance of the data is indicated as follows: *P < 0.05, **P < 0.01, ***P < 0.001, ****P < 0.0001; ns not significant. Source data are provided as a Source data file.

Aiming to further understand the molecular mechanism of this unexpected, unique, and dichotomous behavior of **CU-CPD107**, we successfully obtained the crystal structure of the TLR8/**CU-CPD107** complex (Fig. 4a). **CU-CPD107** utilized hydrogen bonds with A518* and V520* (Fig. 4b, c). This newly identified compound **CU-CPD107** inhibited TLR8 signaling via stabilizing its resting state which was the same mechanism with the first TLR8-specific inhibitor **CU-CPT8m** we have reported previously^19^. The distances between the C termini of the two protomers of TLR8 dimer (TLR8/**CU-CPD107**, 50 Å) was similar to that of the unliganded dimer (51 Å). Superimposition of the TLR8 structure complexed with **CU-CPD107** onto the corresponding unliganded TLR8 segment (residues 32–816) produced a root-mean-square deviation (RMSD) value of 1.2 Å.

Previous reports have shown a two-site activation mechanism for TLR8 with ssRNA degradation products^8^. The primary binding site recognizes dinucleotides (e.g., UG) which are found within the concave leucine-rich repeats of the TLR8 horseshoe structure. The secondary binding site is highly conserved, locating near the dimer interface which recognizes uridine. Even though uridine alone is inactive, its occupation of the secondary site is able to enhance TLR8 signaling in the presence of dinucleotides. Based on the observation that **CU-CPD107** has the same behavior of that with uridine, we utilized the high-resolution X-ray crystal structure of human TLR8/ssRNA40 (PDB: 4R08) as a model and docked **CU-CPD107** to the secondary binding site (Fig. 4d). The docking results suggested that **CU-CPD107** interacts with G351 by additional hydrogen bonding and formed π-π stacking with F405 (Fig. 4e, f). To validate the specific interactions observed, we performed additional in silico alanine scanning of the residues within the **CU-CPD107**-binding site. The reduced association energy predicted with mutants harboring Y348A, G351A, Y353A, and F405A (Fig. 4g) agreed with the docking results and previous works^18,31^ that emphasizes the importance of the additional interactions in ligand recognition. Therefore, we rationalized that ssRNA converts TLR8 to its activated conformation, and **CU-CPD107** binds to the secondary site instead of uridine. This binding by **CU-CPD107** of the secondary site is much tighter than that of uridine, augmenting ssRNA-induced TLR8 activation. This ligand-induced signaling could be a beneficial adjuvant and antiviral agent, as the immune system would only be activated in the presence of ssRNA. To further confirm the function of the specific residues recognizing **CU-CPD107**, we experimentally mutated these residues and tested the activities of **CU-CPD107** in HEK-Blue null1 cells transfected the mutant or wild-type plasmids using Lipofectamine 3000. When mutation of key residues to G351A and F405A, the synergistic activities were decreased (Fig. 4h), confirming that these residues were essential for binding to the **CU-CPD107**.

## Discussion

The precise regulation of innate immunity plays a very important role in clinical application, especially in microbial infection which may induce a deadly inflammatory cytokine storm. The innate immune system is critical, not only for the first line of defense to fight against bacterial or viral infections but also for the activation of the adaptive immune response. Here, we reported the identification of a selective, dual-activity small-molecule **CU-CPD107** which demonstrated differential activity against the small-molecule TLR8 agonists and ssRNA ligands. In the presence of R848, **CU-CPD107** acted as a TLR8 signaling inhibitor. Not only did this inhibitor exhibit selectivity favoring TLR8 over the closely related TLR7 but it also showed no significant toxicity, which may be used as a chemical tool in cell-culture studies. Far more importantly, the activity of **CU-CPD107** was reversed when TLR8 signaling was induced by ssRNA and synergistic agonism was observed. This was observed from both the SEAP assay at the cellular level and ITC assay at the protein level. In the absence of all other ligands, **CU-CPD107** showed no pure agonistic activity, addressing a major challenge that has existed for previous TLR7 and TLR8 agonists as vaccine adjuvants or antiviral drugs. We believe this dichotomous characteristic should have great appeal beyond just vaccine adjuvants. This type of dormancy would be advantageous for any potential antiviral therapeutic, since only tissue affected with PAMPs, like ssRNA viruses, would elicit an inflammatory immune response and healthy tissue would remain unaffected, avoiding unwanted immune responses. This first-of-a-kind and previously unconsidered activity should change the way vaccine adjuvant and TLR researchers approach their own efforts in the future.

## Methods

### Cell culture

HEK-Blue cells stably transfected with TLR1-TLR9 and an NF-κB SEAP reporter were used to assess the potency and specificity of compounds. HEK-Blue hTLR cells were either purchased from Invivogen or prepared in-house. Cells were grown in Dulbecco’s modified Eagle’s medium (DMEM) media with 10% (v/v) heat-inactive FBS, 50 U/ml penicillin, and 50 mg/mL streptomycin. To select for TLR and reporter expression, HEK-Blue hTLR2 cells and HEK-Blue hTLR4 cells were maintained in presence of 100 μg/mL Normocin (Invivogen, No. ant-nr-1) and 1× HEK-Blue Selection (Invivogen, No. hb-sel). Other HEK-Blue hTLR cells were grown in the complete media supplemented with 10 μg/mL blasticidin (Invivogen, No. ant-bl-05) and 100 μg/mL zeocin (Invivogen, No. ant-zn-05). HEK-Blue Null1 cells were grown in media in the presence of 100 μg/mL Normocin and 100 μg/mL Zeocin. All cells were incubated at 37 °C in a humidified atmosphere of 5% CO_2_.

### Secreted embryonic alkaline phosphatase (SEAP) assay

In total, 50,000 cells/well were plated in a tissue-culture treated 96-well plate (Costar, No. 3596) in unsupplemented DMEM. Cells were then treated with 100 μM R848 (InvivoGen, No. tlrl-r848), 5 μg/mL ssRNA40/LyoVec (InvivoGen, No. tlrl-lrna40), and/or 5 μg/mL ORN06/LyoVec (InvivoGen, No. tlrl-orn6) and the indicated concentrations of the compounds. Cells were incubated overnight at 37 °C in a humidified atmosphere of 5% CO_2_ and assayed for NF-κB signaling. Cell media was assayed with Quanti-Blue (InvivoGen, No. rep-qbs3) per the manufacturer’s recommendations. Specificity test in HEK-Blue hTLRs cells for **CU-CPD107** using TLR-specific ligands to selectively activate the corresponding TLRs: 100 ng/mL of Pam_3_CSK_4_, 100 ng/mL of Pam_2_CSK_4_, 5 μg/mL of polyriboinosinic: polyribocytidylic acid [poly(I:C)], 20 ng/mL of LPS (lipopolysaccharide), 50 ng/mL of Flagellin, 2.9 µM of R848, 22.8 µM of R848, and 0.5 μM of ODN2006 were used to selectively activate hTLR1/2, hTLR2/6, hTLR3, hTLR4, hTLR5, hTLR7, hTLR8, and hTLR9 cells, respectively. The SEAP reporter is constructed as a IFN-β promoter fused to five NF-κB and AP-1-binding sites. SEAP was quantified by measuring absorbance at 620 nm. Data were normalized with ligand + DMSO as 100% activity and untreated cells as 0. Each data point represents the average and standard deviation of at least three biological replicates.

### ELISA analysis in human PBMCs

Fresh whole blood donated from healthy volunteers was collected with informed consent under the Institution Review Board (IRB) of Peking Union Medical College Hospital (PUMCH)-approved protocol. PBMCs-related experiments have been described and approved by the IRB of PUMCH (No. S-478), consistent with Institutional Guidelines. Human PBMCs were separated from fresh blood using Density Gradient Centrifugation^32^. After separation, cells were seeded at a density of 6 × 10^5^ cells/well in 96-well plates immediately in 0.2 mL of Roswell Park Memorial Institute (RPMI) 1640 medium supplemented with 10% (v/v) heat-inactive FBS, 100 U/ml penicillin, 100 μg/L streptomycin, 2 mM l-glutamine, and 0.05 mM 2-mercaptoethanol and cultured at 37 °C with humidified air containing 5% CO_2_. Specificity test in PBMCs for **CU-CPD107** using TLR-specific ligands to selectively activate the corresponding TLRs: 10 μg/mL of Pam_3_CSK_4_, 10 μg/mL of Pam_2_CSK_4_, 0.5 mg/mL of LPS (lipopolysaccharide), 1 μg/mL of Flagellin, 0.5 mg/mL of R837, 1 μg/mL of TL8-506 and 5 μM of ODN2006 were used to selectively activate hTLR1/2, hTLR2/6, hTLR4, hTLR5, hTLR7, hTLR8, and hTLR9 cells, respectively. Various concentrations of **CU-CPD107** (0, 12.5, 25, 50, 100 μM) were added and co-incubated at 37 °C in a humidified atmosphere of 5% CO_2_. After 24 h, the supernatant was collected and used for the measurement of TNF-α using BD OptEIATM human TNF-ELISA kit (BD Biosciences, No. 555212) according to the manufacturer’s instructions. To test the synergism of **CU-CPD107** in PBMCs stimulated by ssRNA, cells were incubated with indicated concentrations of ORN06 followed by the treatment of **CU-CPD107**. The production of TNF-α (6 h after treatment) and IFN-α (24 h after treatment) in the supernatant were measured using BD OptEIATM human TNF-ELISA kit or human IFN-α-ELISA kit (Multisciences, No. EK199-96) according to the manufacturers’ instructions.

### WST1 toxicity assay

Cellular toxicity was assessed using the Roche Cell Proliferation Reagent WST1 per the manufacturer’s recommendation. Briefly, WST1 was diluted 1:10 into cell-containing media and assayed with absorbance at 450 nm. Data were normalized with untreated cells as 100% viability, and 25% DMSO treated samples as 0. Each data point represents the average and standard deviation of at least three biological replicates.

### RT-qPCR analysis

Seed HEK-Blue hTLR8 cells in a 12-well plate with 1 × 10^6^ cells/well. Replaced the medium by fresh medium containing no serum after 24-h incubation. Then, the cells were treated or untreated as below: ssRNA40 (5 μg/mL), ssRNA40 (5 μg/mL) with **CU-CPD107** (100 μM), **CU-CPD107** (100 μM) alone, R848 (22.8 µM), or R848 (22.8 µM) with **CU-CPD107** (100 μM), respectively. ORN06 (5 μg/mL) was also used in this experiment instead of ssRNA40, and uridine was used as a positive control. After 8-h treatment, cells were washed with PBS. TRIZOL reagent (Invitrogen, No. 15596026) was used to extract total RNA following standard protocols. iScript^TM^ cDNA Synthesis Kit (Bio-rad, No.1708890) and iTaq Universal SYBR Green Supermix (Bio-Rad, No.1725120) were used to perform reverse transcription and qPCR. TNF-α, IFN-β, IL-1β, IL-6, IL-8, and GAPDH primers were synthesized from Ruibiotech, and the primer sequences are listed in Supplementary Table 2. The ΔΔCt method was used to analyze the data. The relative mRNA levels of cytokine genes were compared with GAPDH gene and normalized to control. RT-qPCR performed in human PBMCs followed the similar procedure mentioned above. ORN06 (2 μg/mL) or TL8-506 (1 μg/mL) were used to stimulate the cells instead of ssRNA40 or R848. In all, 1 × 10^7^ cells were collected to extract total RNA for each data point.

### Protein expression, purification, and crystallization

The ectodomain of hTLR8 (residues 27–827) was prepared as described previously^18^. The sample was stocked in the concentration of 8.2 mg/mL in the solution of 150 mM NaCl and 10 mM Tris-HCl (pH 8.0). For the co-crystallization, we prepared the protein solution containing 7.0 mg/mL hTLR8 with an equimolar 12 mer phosphorothioate RNA (CCCCCGCCCCCC) and a tenfold excess of **CU-CPD107** in the buffer composed of 150 mM NaCl, 10 mM Tris-HCl (pH 8.0), and 5% DMSO. Crystallization trials were performed at 293 K with sitting-drop vapor-diffusion methods. Crystals of TLR8/**CU-CPD107** were obtained by mixing the equivolume of the protein solution and the reservoir solutions containing 0.2 M NaCl, 20-27% (w/v) PEG 1000, and 0.1 M MES-NaOH (pH 6.8).

### Data collection and structure determination

X-ray diffraction dataset of TLR8/**CU-CPD107** crystals was collected on the beamline PF NE3A at Photon Factory (Ibaraki, Japan). The experiment was conducted under the cryogenic condition at 100 K, with a wavelength of 1.0000 Å. The dataset was processed using the XDS program^33^. The initial phase for TLR8/**CU-CPD107** structure was determined by the molecular replacement method using the Molrep program^34^ with a search model based on the ligand-removed TLR8/**CU-CPT8m** structure (PDB ID: 5WYX). Further model refinement was conducted with stepwise cycles of manual model building with the COOT program^35^ and restrained refinement with REFMAC^36^ until the R factor was converged. **CU-CPD107** molecules, N-glycans, and water molecules were built into the electron density map at the latter cycles of the refinement. The stereochemical quality of the final structure was evaluated with the PDB validation server (http://wwpdb-validation.wwpdb.org/). The Ramachandran plot showed that the favored and the allowed regions were 91% and 8% residues for TLR8/**CU-CPD107**, respectively. The electron-density map around **CU-CPD107** is shown in Supplementary Fig. 9. The statistics of the data collection and refinement are summarized in Supplementary Table 3. The coordinate and the structure factor of TLR8/**CU-CPD107** were deposited in the Protein Data Bank with PDB ID 7CRF.

### Isothermal titration calorimetry (ITC) assay

ITC experiments were performed at 298 K in the buffer condition of 0.20 M NaCl, 25 mM MES (pH 5.5), and 5% DMSO using a MicroCal iTC200 (GE Healthcare). The titration sequence started with a single injection (0.4 μL) and was followed by 18 injections (2 μL each), spaced by 120 s between injections. The titration conditions were as follows: 100 μM **CU-CPD107** into 10 μM hTLR8; 100 μM R848 into 10 μM hTLR8; 100 μM R848 into 10 μM hTLR8 in the presence of 100 μM **CU-CPD107**; 100 μM **CU-CPD107** into 10 μM hTLR8 in the presence of 20 μM ORN06; 1 mM uridine into 20 μM hTLR8; 200 μM ORN06 into 20 μM TLR8; 200 μM uridine into 20 μM TLR8 and 40 μM ORN06; 100 μM **CU-CPD107** into 40 μM ORN06. The raw ITC data for each condition were processed by OrigineLab software (GE Healthcare).

### Molecular docking

**CU-CPD107** was docked against a complex of TLR8/ssRNA40 (PDB: 4R08) by AutoDock 4.2^37^. Chem3D ultra 8.0 was used to construct the three-dimensional conformation structure of **CU-CPD107**. A box of 60 × 60 × 60-point grid (0.375-Å spacing between the grid points) and the affinity maps were generated and processed using AutoGrid 4.2 by default setting. For each docking case, 200 Lamarckian genetic algorithm runs were processed by default setting using AutoDock 4.2. The top-scored hit was chosen and visualized for further analysis. CueMol (http://www.cuemol.org) or PyMOL (http://www.pymol.org) was used to prepare all the molecular graphics.

### In silico alanine scanning

The structure of the predicted TLR8/ssRNA40/**CU-CPD107** complex was processed by default setting using the Protein Preparation Wizard in Schrödinger suite 2018-1^38^. The binding pocket was defined by identifying residues in direct contact with **CU-CPD107**, including F261, Y348, I349, K350, G351, S352, Y353, V378, F405, R429, V520, D543, D545, G572, V573, and T574. To validate the role of these residues in the ligand binding, each residue was mutated to alanine in silico within Maestro. Then, the relative binding free energy change (∆∆G) of each mutant over the wild type was calculated using the Prime-molecular mechanics/generalized Born surface area (MM/GBSA) method, keeping the ligand and other residues fixed.

### Plasmids and mutagenesis

The hTLR8 expression plasmid (pcDNA3-TLR8) was constructed by inserting hTLR8 gene (from pcDNA3-TLR8-YFP) into the pcDNA3 expression vector. pcDNA3-TLR8-YFP was a gift from Doug Golenbock (Addgene plasmid # 13024). Mutations were generated into pcDNA3-TLR8 plasmid via Fast Mutagenesis System kit (Transgen, No. FM111). All the sequences of hTLR8 and variants were confirmed by the sequence analysis at Genwiz Inc.

### Activities of CU-CPD107 in mutant TLR8 and wtTLR8

HEK-Blue Null1 cells were seeded in 24-well plates at a density of 1 × 10^4^ cells per well. After 24 h, media were replaced with fresh complete growth media and transiently transfected with pcDNA3-TLR8 or various mutant plasmids (500 ng) using Lipofectamine 3000 reagent (Invitrogen, No. L3000015). After 36 h transfection, cells were stimulated with 5 μg/ml ORN06, or 11.4 µM R848 followed by the addition of 100 μM **CU-CPD107**. After 24 h, 50 μL of supernatant was collected in a new 96-well plate and add 50 μL of Quanti-Blue. The mixture was incubated at 37 °C for 20–40 min, and the plate was measured on a Thermo Varioskan Flash microplate spectrophotometer at 620 nm. Data were normalized as readouts of ligand-treated cells are 100% activation and untreated cells are 0.

### Statistical analysis

Data are displayed as individual points or as mean ± s.d., with sample size indicated in the figure legend. Statistical significance was evaluated using GraphPad Prism 6.0 for mac. A one-way analysis of variance with Bonferroni’s multiple comparisons test for multiple comparisons or Student’s two-sample unpaired *t* test was used for statistical analysis. Statistical significance of the data is indicated as follows: *P < 0.05, **P < 0.01, ***P < 0.001, ****P < 0.0001; ns = not significant.

### Reporting summary

Further information on research design is available in the Nature Research Reporting Summary linked to this article.

## Supplementary information

Supplementary Information

Reporting Summary

## Data Availability

All data generated during this study are included in this manuscript and available for download. The final atomic coordinates and experimental structure factors were deposited in the Protein Data Bank with accession codes 7CRF for the TLR8/**CU-CPD107** complex. Structure information of TLR8/**CU-CPT8m** and TLR8/ssRNA40 mentioned in the manuscript are also available in the Protein Data Bank with accession codes 5WYX and 4R08, respectively. Source data are provided with this paper.

## Source data

Source Data
