# Supplementary material for: Tetrasubstituted imidazoles as incognito Toll-like receptor 8 a(nta)gonists
Source: Nat Commun. 2021 Jul 16;12:4351. doi: 10.1038/s41467-021-24536-4 (PMC8285539; doi:10.1038/s41467-021-24536-4)
Supplement: Supplementary file 1 — Supplementary Information [file 41467_2021_24536_MOESM1_ESM.pdf]

Supplementary Information

Tetrasubstituted Imidazoles as Incognito Toll-like Receptor 8 A(nta)gonists

Yi Yang[1,2#], Adam Csakai[3#], Shuangshuang Jiang[1,2#], Christina Smith[3], Hiromi Tanji[4], Jian Huang[1,2], Torey Jones[3], Kentaro Sakaniwa[4], Lindsey Broadwell[3], Chengrui Shi[1], Subada Soti[3], Umeharu Ohto[4], Yaohui Fang[5], Shu Shen[5], Fei Deng[5], Toshiyuki Shimizu[4] and Hang Yin[1]*

[1] *School of Pharmaceutical Sciences, Tsinghua University-Peking University Joint Center for Life Sciences, Tsinghua University, Beijing 100082, China*

[2] *Department of Chemistry, Tsinghua University, Beijing 100082, China*

[3] *Department of Chemistry & Biochemistry and the BioFrontiers Institute, University of Colorado Boulder, Boulder, Colorado 80309-0596, United States of America*

[4] *Graduate School of Pharmaceutical Sciences, The University of Tokyo, Tokyo 113-0033, Japan*

[5] *Wuhan Institute of Virology, Chinese Academy of Sciences, Wuhan 430071, China*

[#] These authors contributed equally to this work.

*Corresponding Author: yin_hang@tsinghua.edu.cn

Contents

Supplementary Fig. 1 Key interactions of imidazoquinolines binding to TLR8.

Supplementary Fig. 2 The effects of **CU-CPD107** on the mRNA level of downstream cytokines by RT-qPCR in HEK-Blue hTLR8 cells.

Supplementary Fig. 3 **CU-CPD107** could synergistically upregulate the mRNA levels of IL-8 and TNF-α with the presence of 5 μg/mL ORN06.

Supplementary Fig. 4 The effects of **CU-CPD107** on the mRNA level of downstream cytokines by RT-qPCR in human PBMCs.

Supplementary Fig. 5 The effects of **CU-CPD107** in induction of TNF-α in human PBMCs in the presence of ORN06.

Supplementary Fig. 6 **CU-CPD107** showed neglected toxicity in human PBMCs.

Supplementary Fig. 7 The activities of the compounds in the presence of 5 μg/mL ssRNA40.

Supplementary Fig. 8 ITC experiments with TLR8.

Supplementary Fig. 9 Electron density map around **CU-CPD107**.

Supplementary Table 1 Toxicity of the tetrasubstituted imidazoles analogues.

Supplementary Table 2 Primer lists.

Supplementary Table 3 Data collection and refinement statistic for hTLR8/**CU-CPD107**.

General Chemistry Methods

Supplementary Fig. 10-20 Chemical synthetic routes of compounds.

Compounds synthesis and characterization

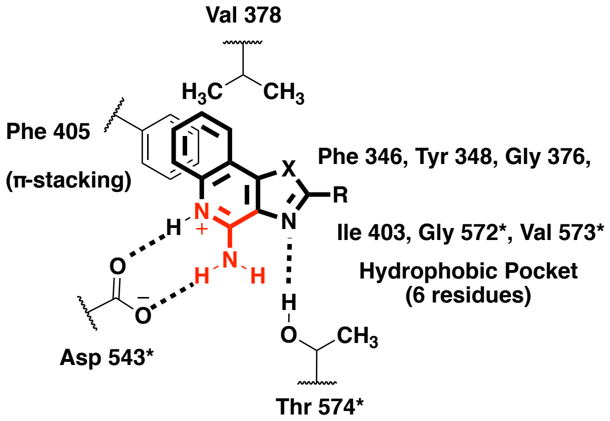

Supplementary Fig. 1 Key interactions of imidazoquinolines binding to TLR8 are shown (residues marked with * indicate that they are located on a different TLR8 protein in the dimer complex from unmarked residues).

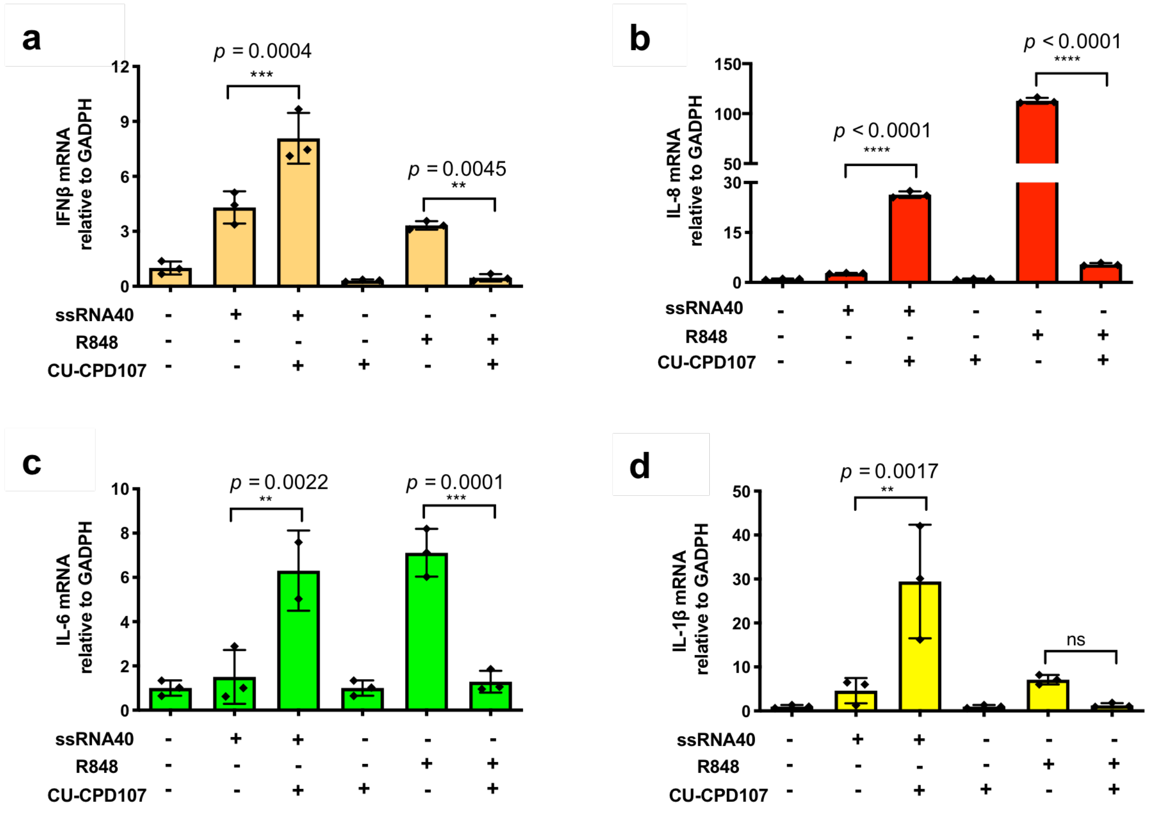

Supplementary Fig. 2 The effects of **CU-CPD107** on the mRNA level of downstream cytokines by RT-qPCR in HEK-Blue hTLR8 cells. **CU-CPD107** could synergistically upregulate the mRNA levels of IFN-β (**a**), IL-8 (**b**), IL-6 (**c**), and IL-1β (**d**) in the presence of 5 μg/mL ssRNA40, while it could inhibit R848-induced activation and itself had no effect in HEK-Blue hTLR8 cells. Data are mean ± s.d.; the data shown are representative of three independent experiments. A one-way analysis of variance with Bonferroni's multiple comparisons test for multiple comparisons was used for statistical analysis. Statistical significance of the data is indicated as follows: *p < 0.05, **p < 0.01, ***p < 0.001, ****p < 0.0001; ns = not significant. Source data are provided as a Source data file.

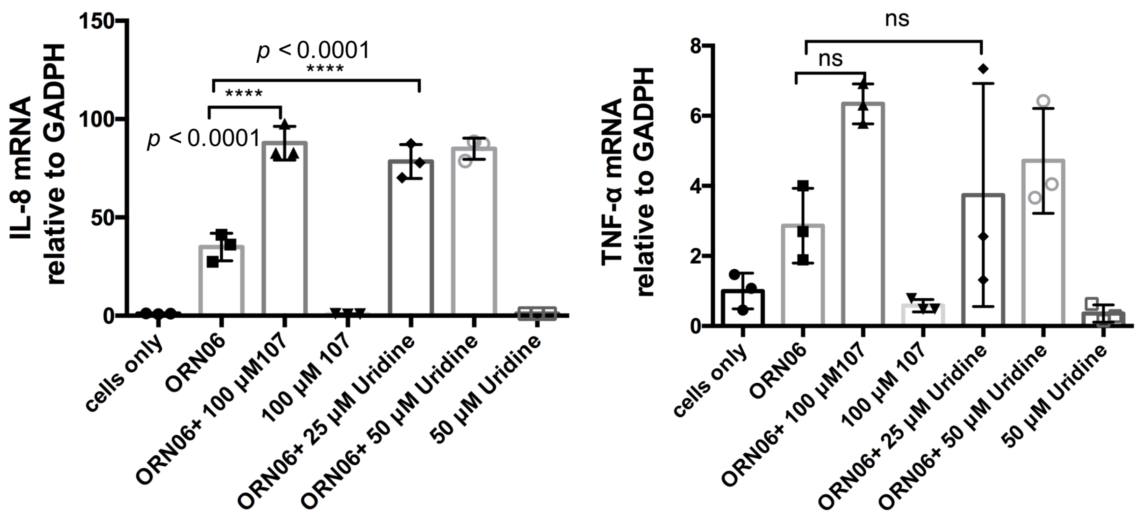

Supplementary Fig. 3 CU-CPD107 could synergistically upregulate the mRNA levels of IL-8 and TNF-α with the presence of 5 μg/mL ORN06. Uridine was used as a positive control and itself has no effects on TLR8 signaling in HEK-Blue TLR8 cells. Data are mean ± s.d.; the data shown are representative of three independent experiments. A one-way analysis of variance with Bonferroni's multiple comparisons test for multiple comparisons was used for statistical analysis. Statistical significance of the data is indicated as follows: *p < 0.05, **p < 0.01, ***p < 0.001, ****p < 0.0001; ns = not significant. Source data are provided as a Source data file.

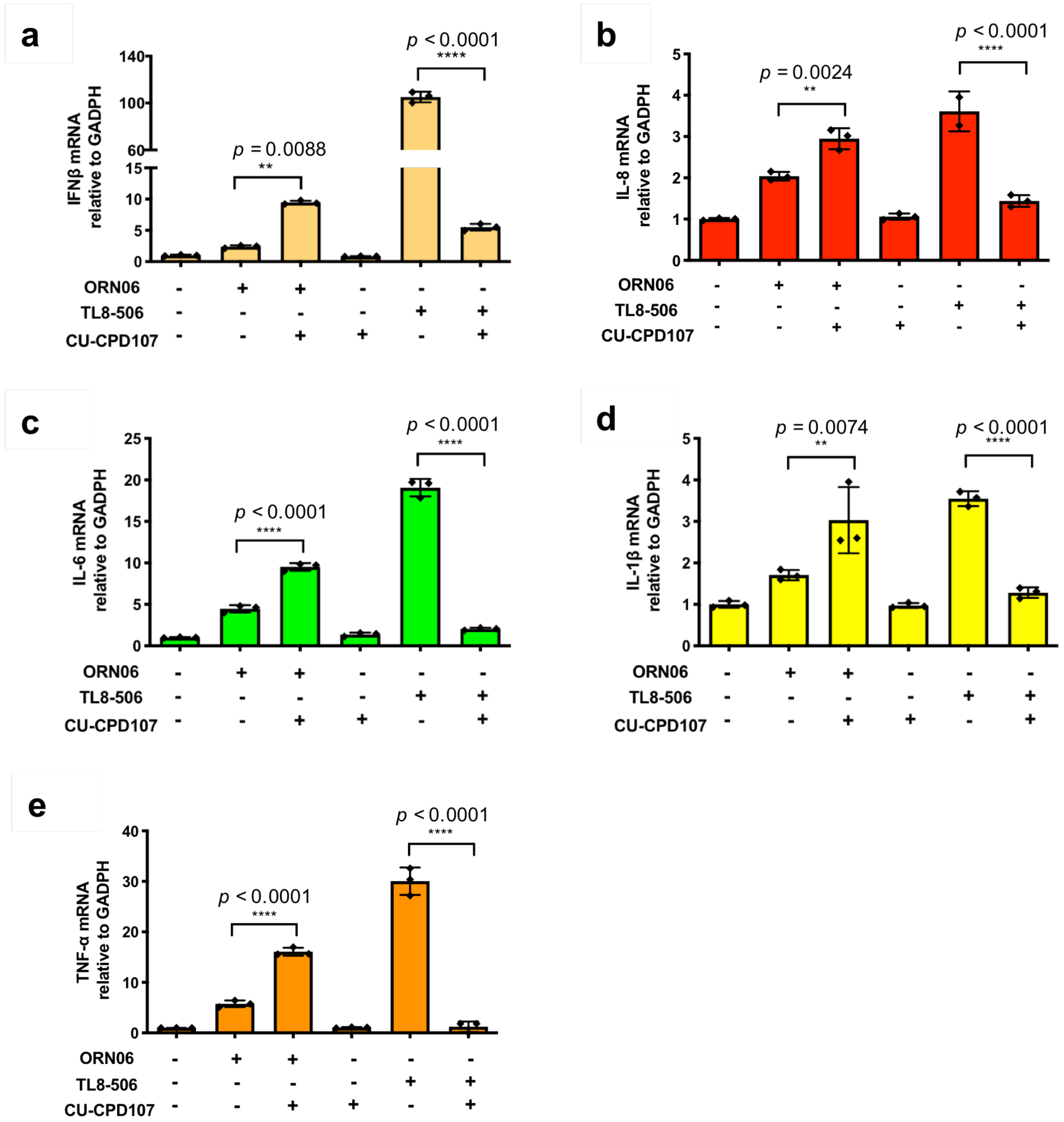

Supplementary Fig. 4 The effects of **CU-CPD107** on the mRNA level of downstream cytokines by RT-qPCR in human PBMCs. **CU-CPD107** could synergistically upregulate the mRNA levels of IFN-β (**a**), IL-8 (**b**), IL-6 (**c**), IL-1β (**d**), and TNF-α (**e**) in the presence of 2 μg/mL ORN06, while it could inhibit 1 μg/mL TL8-506-induced activation whereas itself had no effect in PBMCs. Data are mean ± s.d.; the data shown are representative of three independent experiments with three independent blood donors. A

one-way analysis of variance with Bonferroni's multiple comparisons test for multiple comparisons was used for statistical analysis. Statistical significance of the data is indicated as follows: $*p < 0.05$, $**p < 0.01$, $***p < 0.001$, $****p < 0.0001$; ns = not significant. Source data are provided as a Source data file.

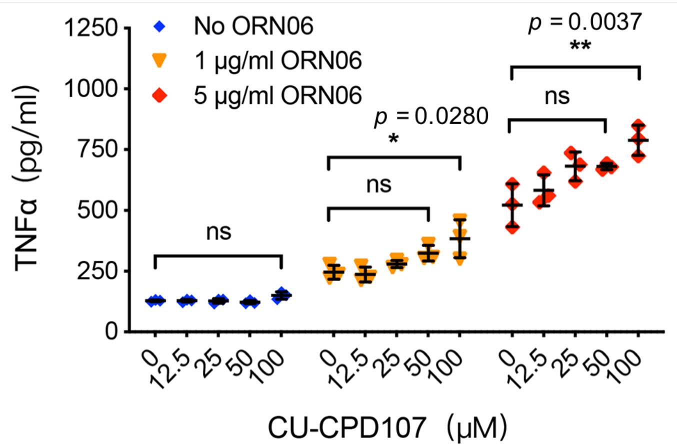

Supplementary Fig. 5 The effects of **CU-CPD107** in induction of TNF-α in human PBMCs in the presence of ORN06. The production of TNF-α were measured 6 hours after treatment of ORN06. Data are mean ± s.d.; the data shown are representative of six independent experiments with six independent blood donors. A one-way analysis of variance with Bonferroni's multiple comparisons test for multiple comparisons was used for statistical analysis. Statistical significance of the data is indicated as follows: *$p < 0.05$, **$p < 0.01$, ***$p < 0.001$, ****$p < 0.0001$; ns = not significant. Source data are provided as a Source data file.

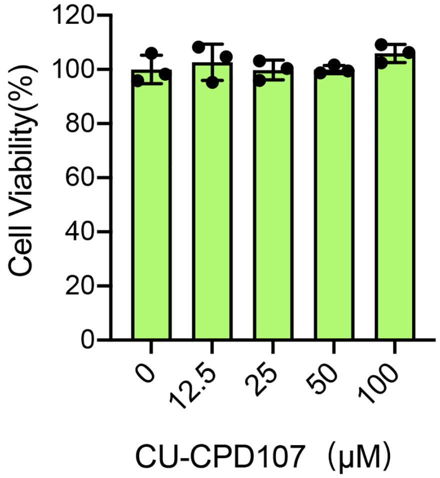

Supplementary Fig. 6 CU-CPD107 showed neglected toxicity in human PBMCs. Data are normalized to **CU-CPD107** untreated cells as 100%. Data are mean ± s.d.; n = 3 biologically independent experiments. Source data are provided as a Source data file.

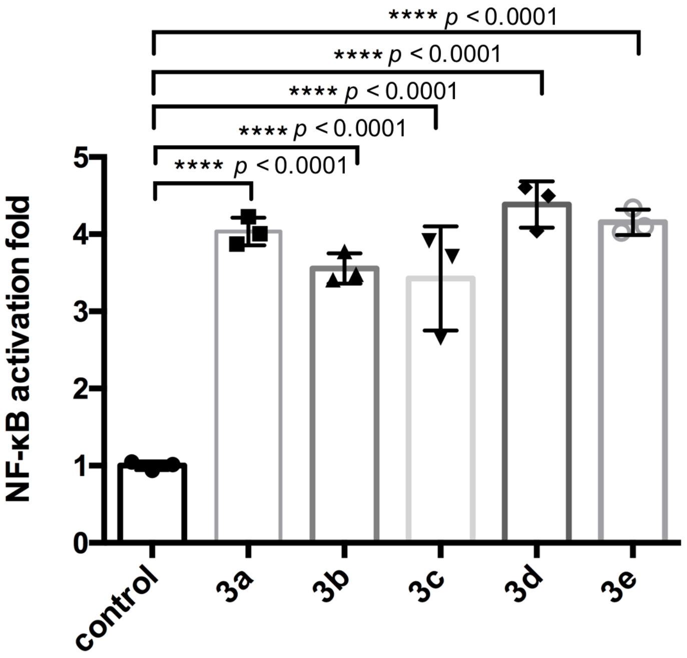

Supplementary Fig. 7 The activities of the compounds in the presence of 5 μg/mL ssRNA40. The TLR8 activation fold induced by **3a**, **3b**, **3c**, **3d** and **3e** at 100 μM in the presence of ssRNA (5 μg/mL) compared to ssRNA (5 μg/mL) control. Data are mean ± s.d.; n = 3 biologically independent experiments. A one-way analysis of variance with Bonferroni's multiple comparisons test for multiple comparisons was used for statistical analysis. Statistical significance of the data is indicated as follows: *p < 0.05, **p < 0.01, ***p < 0.001, ****p < 0.0001; ns = not significant. Source data are provided as a Source data file.

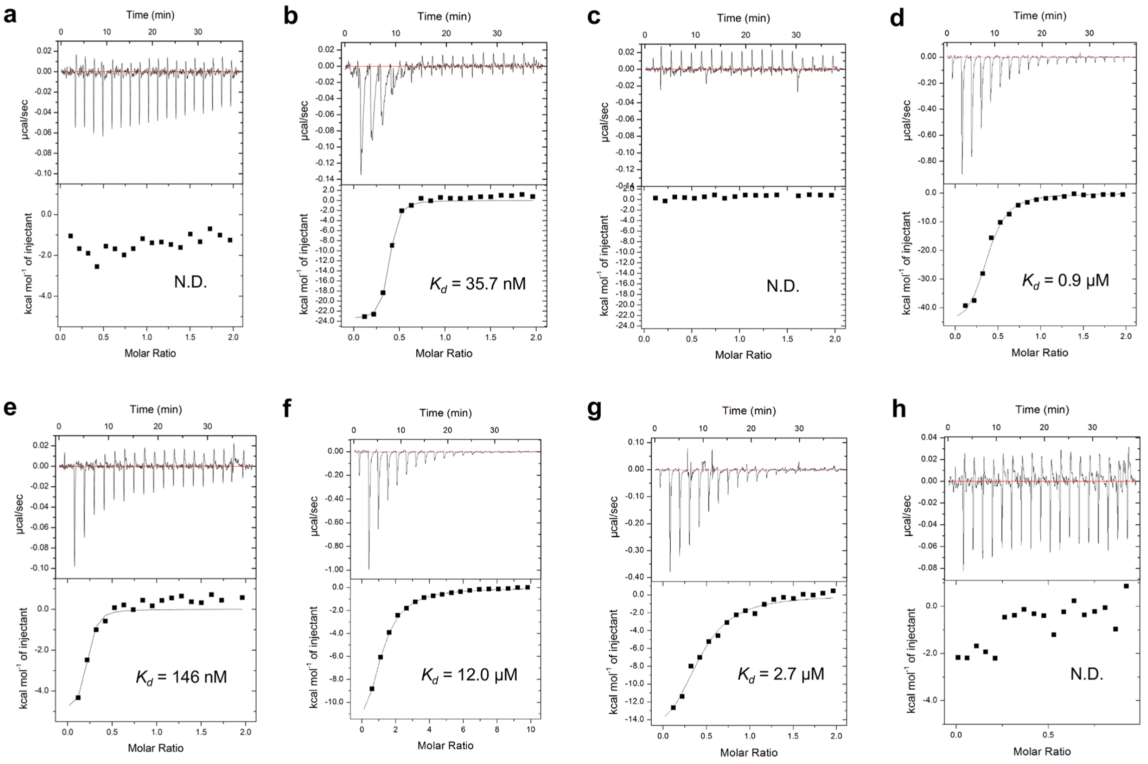

Supplementary Fig. 8 ITC experiments with TLR8. a CU-CPD107 (100 µM) was titrated into TLR8 (10 µM), showing little heat change. **b** R848 (100 µM) was titrated into TLR8 (10 µM), showing a binding curve with a K_d = 35.7 nM. **c** R848 (100 µM) was titrated into TLR8 (10 µM) with **CU-CPD107** (100 µM), showing inhibited binding by no heat change. **d** ORN06 (200 µM) was titrated to TLR8 (20 µM) with a K_d = 0.9 µM. **e CU-CPD107** (100 µM) was titrated into TLR8 (10 µM) with ORN06 (20 µM), showing a binding curve with a K_d = 146 nM. **f** Uridine (1 mM) was titrated to TLR8 (20 µM) with a K_d = 12 µM. **g** Uridine (200 µM) was titrated to TLR8 (20 µM) and ORN06 (40 µM) with a K_d = 2.7 µM. **h CU-CPD107** (100 µM) was titrated to ORN06 (40 µM) which could not evaluate a K_d-value due to little heat change.

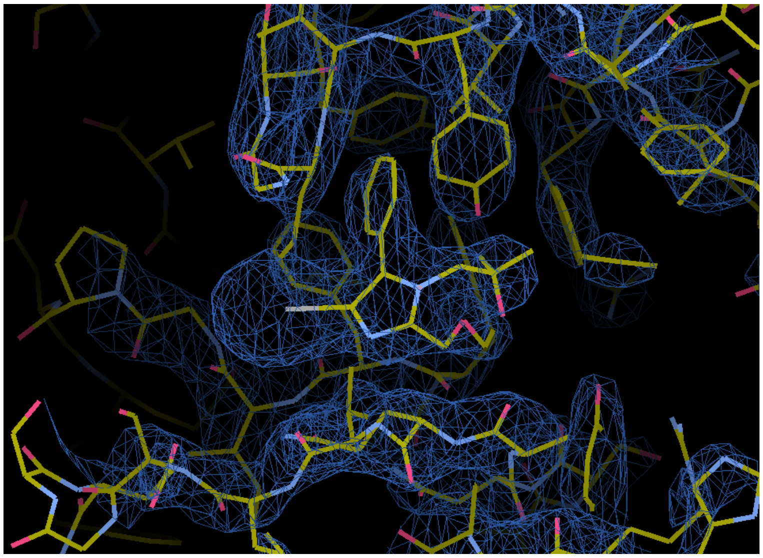

Supplementary Fig. 9 Electron density map around **CU-CPD107**.

Supplementary Table 1 Toxicity of the tetrasubstituted imidazoles analogues.

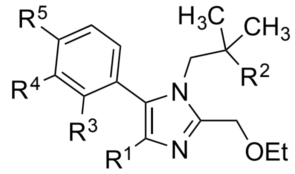

compounds	R1	R2	R3	R4	R5	Cell viability[a]
**1a**	-CN	-OH	-H	-H	-H	0.7 ± 0.3
**1b**	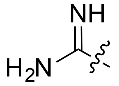	-OH	-H	-H	-H	0.7 ± 0.2
**1c**	-$CONH_2$	-OH	-H	-H	-H	0.8 ± 0.3
**1d**	-CHO	-OH	-H	-H	-H	0.7 ± 0.2
**1e**	-$COOCH_3$	-OH	-H	-H	-H	0.9 ± 0.1
**1f**	-CH_2OH	-OH	-H	-H	-H	0.8 ± 0.1
**1g**	-CH_2OCH_3	-OH	-H	-H	-H	0.8 ± 0.2
**1h**	-CH_3	-OH	-H	-H	-H	0.8 ± 0.3
**1i**	-CH_2F	-OH	-H	-H	-H	0.9 ± 0.3
**1j**	-$C(CH_3)_2OH$	-OH	-H	-H	-H	TBD
**1k**	-$CH(CH_3)_2$	-OH	-H	-H	-H	0.8 ± 0.3
**1l**	-Ph	-OH	-H	-H	-H	TBD
**1m**	-H	-OH	-H	-H	-H	0.9 ± 0.2
**1n**	-Cl	-OH	-H	-H	-H	0.9 ± 0.2
**1o**	-Br	-OH	-H	-H	-H	1.1 ± 0.3
**1p (CU-CPD107)**	-I	-OH	-H	-H	-H	0.9 ± 0.2
**1q**	-COOH	-OH	-H	-H	-H	1.1 ± 0.3
**2a**	-H	-H	-H	-H	-H	0.9 ± 0.3

**2b**	-Cl	-H	-H	-H	-H	0.7 ± 0.2
**2c**	-Br	-H	-H	-H	-H	0.7 ± 0.1
**2d**	-I	-H	-H	-H	-H	0.3 ± 0.2
**3a**	-I	-OH	-H	-H	-Ph	0.8 ± 0.1
**3b**	-I	-OH	-H	-Ph	-H	0.8 ± 0.0
**3c**	-I	-OH	$-CH_3$	-H	-H	0.8 ± 0.0
**3d**	-I	-OH	-H	$-CH_3$	-H	0.8 ± 0.0
**3e**	-I	-OH	-H	-H	$-CH_3$	0.9 ± 0.1

[a] Cell viability was measured by WST1 as compared to uninhibited R848 signaling, which was normalized to 1.0. Data are representative of the average and standard deviation of at least three independent experiments.

Supplementary Table 2 Primer lists.

Primer Name	Sequence (5' to 3')
TNF-α F	CCCAGGGACCTCTCTCTAATC
TNF-α R	ATGGGCTACAGGCTTGTCACT
IL-1β F	AACCTCTTCGAGGCACAAG
IL-1β R	GTTTAGGGCCATCAGCTTCA
IL-6 F	GTACATCCTCGACGGCATCTC
IL-6 R	GGCAAGTCTCCTCATTGAATC
IL-8 F	CCAGGAAGAAACCACCGGAAG
IL-8 R	TGGTCCACTCTCAATCACTCTCAG
IFN-β F	AGGACAGGATGAACTTTGAC
IFN-β R	TGATAGACATTAGCCAGGAG
GAPDH F	CCCACTCCTCCACCTTTGACG
GAPDH R	CACCACCCTGTTGCTGTAGCCA

Supplementary Table 3 Data collection and refinement statistic for hTLR8/**CU-CPD107.**

	hTLR8/**CU-CPD107**
**Data collection**	
Space group	*C*2
Cell dimensions	
a, *b*, *c* (Å)	144.3, 100.0, 140.9
α, β, γ (°)	90.0, 105.6, 90.0
Resolution (Å)	2.89
R_{sym} or R_{merge}	0.071 (0.678)
$I / \sigma I$	17.0 (2.8)
Completeness (%)	99.9 (100.0)
Redundancy	6.9 (6.6)
Total No. of reflections	298,805
No. of unique reflections	43,396
**Refinement**	
Resolution (Å)	40.71-2.89
No. of reflections	41,017
R_{work} / R_{free}	0.210/0.268
No. of atoms	
Protein	12,036
Glycan	682
**CU-CPD107**	42
B-factors	
Protein	79.5
Glycan	99.1
**CU-CPD107**	70.7
R.m.s. deviations	
Bond lengths (Å)	0.005
Bond angles (°)	1.48

General Chemistry Methods

NMR spectra were acquired on Bruker 400 spectrometer, running at 400 MHz for ^{1}H and 101 MHz for ^{13}C respectively. ^{1}H NMR spectra were recorded at 400 MHz in DMSO-d_6 using residual DMSO (2.50 ppm) as the internal standard. ^{13}C NMR spectra were recorded at 101 MHz in DMSO-d_6 using residual DMSO (39.52 ppm) as internal reference. Thin layer chromatography was performed on Merck Kieselgel 60 Å F254 plates eluting with the solvent indicated, visualized by a 254 nm UV lamp. Compounds were purified using flash chromatography, (Silica gel 60Å, 230-400 mesh, Sorbent Technologies). Mass spectrometry was performed at the mass spectrometry facility of the Department of Chemistry at University of Colorado at Boulder on a double focusing high resolution mass spectrometer. Unless otherwise noted, analytical grade solvents and commercially available reagents were used without further purification. The purity of tested compounds was evaluated via ^{1}H NMR (>95% sample purity). All compounds tested in the SEAP assay were confirmed via high-resolution mass spectrometry. All other intermediate compounds were confirmed with either high-resolution or standard resolution mass spectrometry, and all compounds were characterized via ^{13}C and/or ^{1}H NMR.

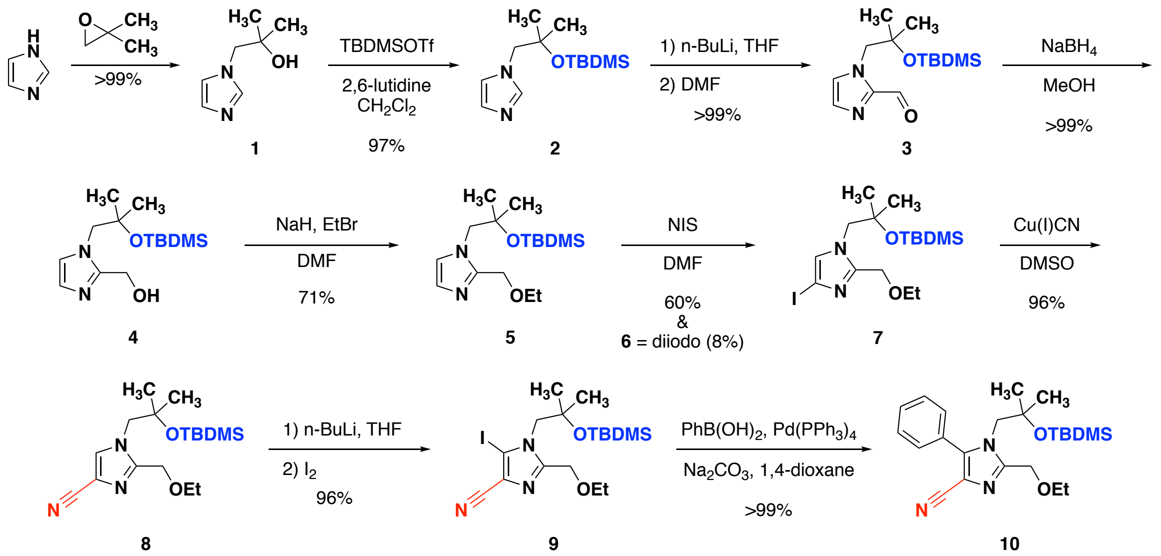

Supplementary Fig. 10 Key intermediate synthesis: Key intermediate **10** was synthesized from imidazole in 38% yield over nine steps.

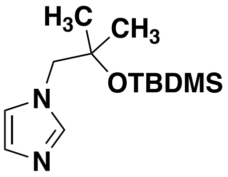

1-{2-[(*tert*-butyldimethylsilyl)oxy]-2-methylpropyl}-1H-imidazole (2)

To a solution of 1-(1H-imidazol-1-yl)-2-methylpropan-2-ol (10.3 g, 73.5 mmol, prepared as previously reported in *Tetrahedron*, 63(2), **2007**, 469-473) and 2,6-lutidine (60.0 mL, 515 mmol) in dichloromethane (900 mL) at room temperature was added *tert*-butyldimethylsilyl trifluoromethanesulfonate (84.5 mL, 368 mmol) via addition funnel over 10 min. The resulting solution was allowed to stir for 16 hours at room temperature. After this time, the reaction mixture was concentrated to roughly 400 mL, washed with water (5 × 400 mL), washed with saturated aqueous sodium chloride (400 mL), dried over sodium sulfate, filtered and concentrated to dryness. The residue obtained was

azeotroped with toluene (3 × 400 mL) and was purified via flash SiO_2 chromatography (100 g silica gel, gradient of dichloromethane to 4% methanol/96% dichloromethane) to give 1-{2-[(*tert*-butyldimethylsilyl)oxy]-2-methylpropyl}-1H-imidazole **(2)** (18.1 g, 97%) as a red oil; ^{1}H NMR (400 MHz, DMSO-d_6) δ 7.51 (s, 1H), 7.06 (t, J = 1.1 Hz, 1H), 6.86 (s, 1H), 3.88 (s, 2H), 1.15 (s, 6H), 0.83 (s, 9H), 0.01 (s, 6H); ^{13}C NMR (101 MHz DMSO-d_6) δ 138.30, 127.41, 120.76, 72.90, 57.87, 26.96, 25.74, 17.71, –2.31; MS (ESI$^+$), calcd $C_{13}H_{26}N_2OSi$ (M+H) = 255.1893, found = 255.1885.

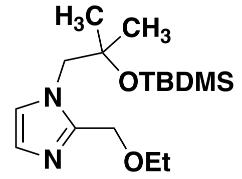

1-{2-[(*tert*-butyldimethylsilyl)oxy]-2-methylpropyl}-2-(ethoxymethyl) 1H-imidazole (5)

A solution of 1-{2-[(*tert*-butyldimethylsilyl)oxy]-2-methylpropyl}-1H-imidazole (15.35 g, 60.33 mmol) in tetrahydrofuran (735 mL) was cooled in a dry ice/isopropyl alcohol bath (~ -78 °C). *n*-Butyllithium (36 mL, 91 mmol, 2.5 M solution in hexanes) was added slowly over 20 min (keeping internal temperature at -55 °C or lower) and the resulting solution was allowed to stir at the same temperature for 40 min. After this time, the reaction flask was transferred to a warmer dry ice/isopropyl alcohol bath (maintained between -40 °C and -25 °C) and was allowed to stir for 1 hour. After this time, the reaction flask was placed back into the original -78 °C bath and was allowed to stir until the internal temperature reached -65 °C. At this time *N*,*N*-dimethylformamide (9.40 mL, 121 mmol) was added and allowed to stir at the same temperature for 30 min. After this

time, the dry ice bath was removed and the reaction was allowed to stir at room temperature for 18 hours. After this time, saturated aqueous ammonium chloride (100 mL) and water (100 mL) was added. The entire biphasic mixture was concentrated via rotary evaporation to remove roughly 500 mL of tetrahydrofuran. The resulting biphasic mixture was further diluted with saturated aqueous ammonium chloride (300 mL) and extracted with ethyl acetate (3 × 200 mL). The combined organic layers were washed with saturated aqueous sodium chloride (300 mL), dried over magnesium sulfate, filtered and concentrated to dryness to give crude 1-{2-[(*tert*-butyldimethylsilyl)oxy]-2-methylpropyl}-1H-imidazole-2-carbaldehyde **(3)** as a yellow oil. This oil was taken up into methanol (450 mL) and cooled in an ice bath. Sodium borohydride (3.42 g, 90.5 mmol) was added and the ice bath was kept for 5 min. After this time, the ice bath was removed and the reaction was allowed to stir at room temperature for 18.5 hours. After this time, saturated aqueous ammonium chloride (300 mL) was added and the resulting mixture was extracted with dichloromethane (3 × 200 mL). The combined organic layers were washed with saturated aqueous sodium chloride (400 mL), dried over sodium sulfate, filtered and concentrated to dryness. The off-white solid obtained was azeotroped with toluene (400 mL), then left on a high-vacuum pump for several hours to give crude (1-{2-[(*tert*-butyldimethylsilyl)oxy]-2-methylpropyl}-1H-imidazol-2-yl)methanol **(4)**. The white solid was taken up into *N*,*N*-dimethylformamide (450 mL) and cooled in an ice bath. Sodium hydride (3.62 g, 90.5 mmol, 60% dispersion in mineral oil) was added and allowed to stir in the ice bath for 30 min. After this time, bromoethane (9.0 mL, 120 mmol) was added. The ice bath was then removed and the reaction was allowed to stir for 18 hours. After this time, saturated aqueous ammonium chloride (100 mL) and water

(150 mL) was added. The resulting mixture was extracted with ethyl acetate (3 × 200 mL). The combined organic layers were then washed with 5% aqueous lithium chloride (3 × 200 mL), washed with saturated aqueous sodium chloride (300 mL), dried over magnesium sulfate, filtered and concentrated to dryness. The resulting residue was purified via flash SiO_2 chromatography (340 g silica gel, gradient of dichloromethane to 5% methanol/95% dichloromethane) to give 1-{2-[(*tert*-butyldimethylsilyl)oxy]-2-methylpropyl}-2-(ethoxymethyl) 1H-imidazole **(5)** (13.45 g, 71% over three steps) as an orange oil; ^{1}H NMR (400 MHz, DMSO-d_6) δ 7.11 (d, J = 1.2 Hz, 1H), 6.82 (d, J = 1.2 Hz, 1H), 4.46 (s, 2H), 3.92 (s, 2H), 3.42 (q, J = 7.0 Hz, 2H), 1.19 (s, 6H), 1.08 (t, J = 7.0 Hz, 3H), 0.82 (s, 9H), 0.04 (s, 6H); ^{13}C NMR (101 MHz DMSO-d_6) δ 144.76, 126.18, 121.86, 73.53, 64.74, 63.80, 56.56, 27.22, 25.77, 17.70, 14.90, –2.23; MS (ESI$^+$), calcd $C_{16}H_{33}N_2O_2Si$ (M+H) = 313.2311, found = 313.2316.

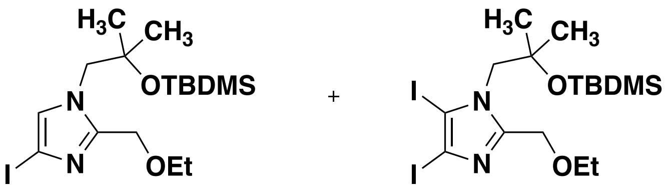

1-{2-[(*tert*-butyldimethylsilyl)oxy]-2-methylpropyl}-2-(ethoxymethyl)-4-iodo-1H-imidazole (7) and 1-{2-[(tert-butyldimethylsilyl)oxy]-2-methylpropyl}-2-(ethoxymethyl)-4,5-diiodo-1H-imidazole (6)

A solution of 1-{2-[(*tert*-butyldimethylsilyl)oxy]-2-methylpropyl}-2-(ethoxymethyl) 1H-imidazole (3.00 g, 9.60 mmol) in *N*,*N*-dimethylformamide (60 mL) was heated to 80 °C. A separate solution of *N*-iodosuccinimide (4.32 g, 19.2 mmol) in *N*,*N*-dimethylformamide (30 mL) was prepared and slowly added to the original reaction flask

over 5 min. The reaction was allowed to stir at 80 °C for 16 hours. After this time, the reaction was allowed to cool to room temperature and water (100 mL) was added. Solid sodium thiosulfate pentahydrate was added until no further color change was observed, resulting in a pale yellow suspension. The resulting mixture was extracted with ethyl acetate (3 × 70 mL). The combined organic layers were then washed with 5% aqueous lithium chloride (3 × 150 mL), washed with saturated aqueous sodium chloride (200 mL), dried over magnesium chloride, filtered and concentrated to dryness. The resulting residue was purified via flash SiO_2 chromatography (100 g silica gel, gradient of hexanes to 25% ethyl acetate/75% hexanes), to give 1-{2-[(*tert*-butyldimethylsilyl)oxy]-2-methylpropyl}-2-(ethoxymethyl)-4-iodo-1H-imidazole **(7)** (2.44 g, 58%) as pale yellow oil; ^{1}H NMR (400 MHz, DMSO-d_6) δ 7.25 (s, 1H), 4.44 (s, 2H), 3.91 (s, 2H), 3.42 (q, J = 7.0 Hz, 2H), 1.17 (s, 6H), 1.08 (t, J = 7.0 Hz, 3H), 0.83 (s, 9H), 0.04 (s, 6H); ^{13}C NMR (101 MHz DMSO-d_6) δ 147.02, 127.62, 80.04, 73.43, 64.94, 63.11, 56.66, 27.05, 25.77, 17.75, 14.90, –2.26; MS (ESI$^+$), calcd $C_{16}H_{32}IN_2O_2Si$ (M+H) = 439.1278, found = 439.1274. Chromatography also gave 1-{2-[(tert-butyldimethylsilyl)oxy]-2-methylpropyl}-2-(ethoxymethyl)-4,5-diiodo-1H-imidazole **(6)** (0.434 g, 8%) as an off-white solid; ^{1}H NMR (400 MHz, DMSO-d_6) δ 4.58 (s, 2H), 4.09 (s, 2H), 3.40 (q, J = 7.0 Hz, 2H), 1.29 (s, 6H), 1.08 (t, J = 7.0 Hz, 3H), 0.75 (s, 9H), 0.05 (s, 6H); ^{13}C NMR (101 MHz DMSO-d_6) δ 150.27, 96.31, 89.26, 74.14, 65.14, 64.68, 58.01, 28.83, 25.92, 17.80, 14.92, -2.02; MS (ESI$^+$), calcd $C_{16}H_{31}I_2N_2O_2Si$ (M+H) = 565.0244, found = 565.0242.

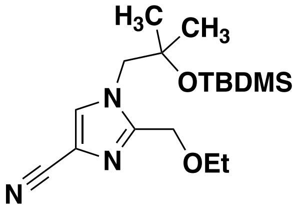

1-{2-[(*tert*-butyldimethylsilyl)oxy]-2-methylpropyl}-2-(ethoxymethyl) 1H-imidazole-4-carbonitrile (8)

A solution of 1-{2-[(*tert*-butyldimethylsilyl)oxy]-2-methylpropyl}-2-(ethoxymethyl)-4-iodo-1H-imidazole (2.00 g, 4.56 mmol) in dimethyl sulfoxide (60 mL) was degassed and backfilled with nitrogen gas (3×). Copper (I) cyanide (0.817 g, 9.12 mmol) was added and the resulting mixture was degassed and backfilled with nitrogen (3×) again. The reaction was heated at 150 °C for 17 hours and then allowed to cool to room temperature. ethyl acetate (300 mL) was added and filtered through a plug of silica gel (~ 70 g). The filtrate was washed with water (3 × 200 mL), washed with saturated aqueous sodium chloride (200 mL), dried over magnesium sulfate, and filtered through an additional plug of silica gel (~ 70 g, eluting with ethyl acetate) to give 1-{2-[(*tert*-butyldimethylsilyl)oxy]-2-methylpropyl}-2-(ethoxymethyl) 1H-imidazole-4-carbonitrile **(8)** (1.467 g, 95%) as a yellow oil; ^{1}H NMR (400 MHz, DMSO-d_6) δ 7.98 (s, 1H), 4.52 (s, 2H), 4.02 (s, 2H), 3.45 (q, J = 7.0 Hz, 2H), 1.21 (s, 6H), 1.09 (t, J = 7.0 Hz, 3H), 0.79 (s, 9H), 0.03 (s, 6H); ^{13}C NMR (101 MHz DMSO-d_6) δ 147.38, 131.98, 115.47, 109.95, 73.14, 65.25, 63.21, 57.05, 27.16, 25.74, 17.69, 14.85, -2.28; MS (ESI^{+}), calcd $C_{17}H_{32}N_3O_2Si$ (M+H) = 338.2264, found = 338.2257.

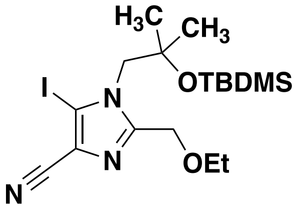

1-{2-[(*tert*-butyldimethylsilyl)oxy]-2-methylpropyl}-2-(ethoxymethyl) 5-iodo-1H-imidazole-4-carbonitrile (9)

A solution of 1-{2-[(*tert*-butyldimethylsilyl)oxy]-2-methylpropyl}-2-(ethoxymethyl) 1H-imidazole-4-carbonitrile (1.467 g, 4.346 mmol) in tetrahydrofuran (60 mL) was prepared in an oven-dried flask and cooled in a dry ice/isopropyl alcohol bath (~ -78 °C). *n*-Butyllithium (3.0 mL, 4.8 mmol, 1.6 M solution in hexanes) was added slowly over 5 min and the reaction was allowed to stir in the ice bath for 30 min. After this time, a solution of iodine (2.21 g, 8.69 mmol) in tetrahydrofuran (9 mL) was added slowly over 10 min. The reaction was allowed to stir in the dry ice bath for an additional 30 min. After this time, the dry ice bath was removed and the reaction was allowed to stir at room temperature for 17 hours. After this time, saturated aqueous ammonium chloride (60 mL) was added and the resulting mixture was extracted with ethyl acetate (3 × 40 mL). The combined organic layers were washed with aqueous sodium thiosulfate pentahydrate (100 mL, 1 M), washed with saturated aqueous sodium chloride (100 mL), dried over magnesium sulfate, filtered and concentrated to dryness. The residue obtained was purified via flash SiO_2 chromatography (100 g silica gel, gradient of dichloromethane to 6% ethyl acetate/94% dichloromethane) to give 1-{2-[(*tert*-butyldimethylsilyl)oxy]-2-methylpropyl}-2-(ethoxymethyl) 5-iodo-1H-imidazole-4-carbonitrile **(9)** (1.928 g, 96%) as an orange solid; ^{1}H NMR (400 MHz, DMSO-d_6) δ 4.63 (s, 2H), 4.10 (s, 2H), 3.43 (q, *J* = 7.0 Hz, 2H), 1.32 (s, 6H), 1.09 (t, *J* = 7.0 Hz, 3H), 0.72 (s, 9H), 0.06 (s, 6H); ^{13}C NMR

(101 MHz DMSO-d_6) δ 150.64, 119.51, 115.66, 91.76, 73.93, 65.38, 64.55, 57.23, 28.93, 25.87, 17.75, 14.87, -2.05; MS (ESI$^+$), calcd $C_{17}H_{31}IN_3O_2Si$ (M+H) = 464.1230, found = 464.1225.

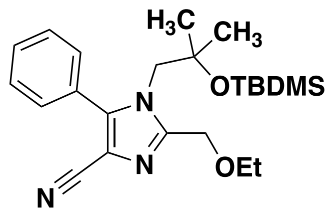

1-{2-[(*tert*-butyldimethylsilyl)oxy]-2-methylpropyl}-2-(ethoxymethyl)-5-phenyl-1H-imidazole-4-carbonitrile (10)

A mixture of 1-{2-[(*tert*-butyldimethylsilyl)oxy]-2-methylpropyl}-2-(ethoxymethyl) 5-iodo-1H-imidazole-4-carbonitrile (2.239 g, 4.831 mmol), phenylboronic acid (0.647 g, 5.31 mmol), and aqueous sodium carbonate (32 mL, 1 M) in 1,4-dioxane (63 mL) was degassed and backfilled with nitrogen gas (4x). Tetrakis(triphenylphosphine)palladium(0) was added and the resulting mixture was degassed and backfilled with nitrogen gas (4x) again. The mixture was heated at 80 °C for 20 hours. After this time, the reaction was allowed to cool to room temperature, diluted with water (200 mL), and extracted with ethyl acetate (3 × 100 mL). The combined organic layers were washed with saturated aqueous sodium chloride (200 mL), dried over magnesium sulfate, filtered and concentrated to dryness. The residue obtained was purified via flash SiO_2 chromatography (100 g silica gel, gradient of hexanes to 15% ethyl acetate/85% hexanes) to give 1-{2-[(*tert*-butyldimethylsilyl)oxy]-2-methylpropyl}-2-(ethoxymethyl)-5-phenyl-1H-imidazole-4-carbonitrile **(10)** (1.99 g, >99%) as a yellow oil; ^{1}H NMR (400 MHz, DMSO-d_6) δ 7.61-7.51 (m, 5H), 4.64 (s, 2H), 4.23 (s, 2H), 3.52 (q, J = 7.0 Hz, 2H), 1.15

(t, J =7.0 Hz, 3H), 0.86 (s, 6H), 0.70 (s, 9H), 0.03 (s, 6H); ^{13}C NMR (101 MHz DMSO-d_6) δ 148.97, 142.61, 129.76, 129.36, 129.17, 127.49, 115.81, 110.52, 73.60, 65.51, 64.56, 54.77, 28.19, 25.72, 17.65, 14.91, -2.27; MS (ESI$^+$), calcd $C_{23}H_{36}N_3O_2Si$ (M+H) = 414.2577, found = 414.2569.

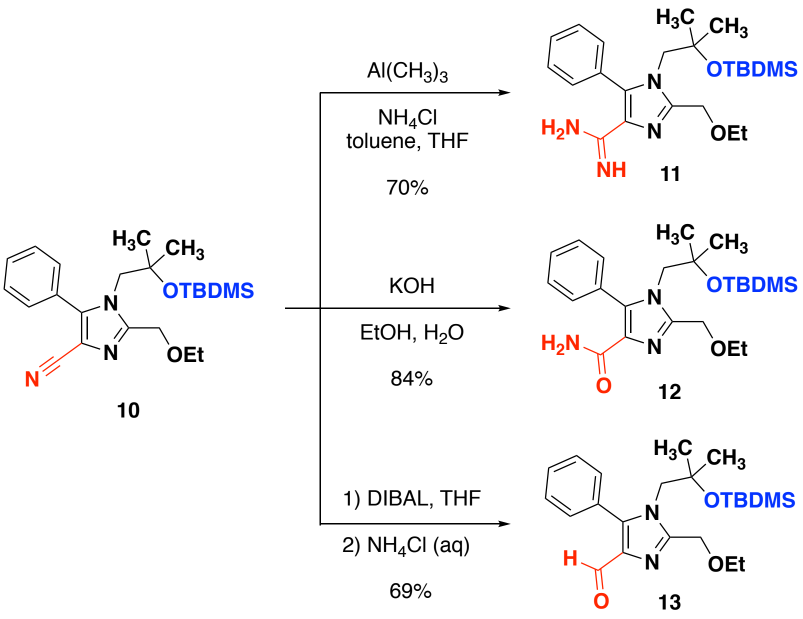

Supplementary Fig. 11 Amidine, amide, and aldehyde analog synthesis: Key intermediate **10** was used to synthesize amidine **11**, primary amide **12**, and aldehyde **13**.

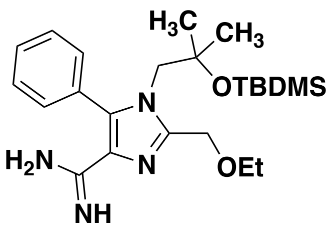

1-{2-[(tert-butyldimethylsilyl)oxy]-2-methylpropyl}-2-(ethoxymethyl)-5-phenyl-1H-imidazole-4-carboximidamide (11)

A mixture of solid ammonium chloride (0.026 g, 0.48 mmol) in toluene (4 mL) was cooled in an ice bath. Trimethylaluminum (0.73 mL, 0.73 mmol, 1 M solution in heptane)

was added over 2 min, and the resulting mixture was allowed to stir in the ice bath for 30 min. After this time, the ice bath was removed and the resulting mixture was allowed to stir at room temperature for 90 min. After this time, 1-{2-[(*tert*-butyldimethylsilyl)oxy]-2-methylpropyl}-2-(ethoxymethyl)-5-phenyl-1H-imidazole-4-carbonitrile (0.100 g, 0.242 mmol) in tetrahydrofuran (1.5 mL) was added and the solution was heated at 80 °C for 17 hours. After this time, the solution was allowed to cool to room temperature and saturated aqueous sodium bicarbonate (10 mL) was added, followed by saturated aqueous potassium sodium tartrate (30 mL), and the resulting mixture was extracted with ethyl acetate (3 × 30 mL). The combined organic layers were washed with saturated aqueous sodium chloride (50 mL), dried over magnesium sulfate, filtered and concentrated to dryness. The residue obtained was purified via flash SiO_2 chromatography (10 g silica gel, gradient of dichloromethane to 20% [1% triethylamine/99% methanol]/80% dichloromethane) to give 1-{2-[(tert-butyldimethylsilyl)oxy]-2-methylpropyl}-2-(ethoxymethyl)-5-phenyl-1H-imidazole-4-carboximidamide **(11)** (0.075 g, 72%) as a white solid; ^{1}H NMR (400 MHz, DMSO-d_6) δ 8.38 (br s, 3H), 7.60–7.55 (m, 3H), 7.54-7.50 (m, 2H), 4.70 (s, 2H), 4.12 (s, 2H), 3.55 (q, J = 7.0 Hz, 2H), 1.16 (t, J = 7.0 Hz, 3H), 0.83 (s, 6H), 0.75 (s, 9H), -0.01 (s, 6H); ^{13}C NMR (101 MHz DMSO-d_6) δ 159.50, 147.67, 137.48, 130.84, 130.05, 129.33, 127.37, 125.33, 73.57, 65.60, 64.54, 54.50, 28.21, 25.84, 17.73, 14.96, -2.17; MS (ESI$^+$), calcd $C_{23}H_{39}N_4O_2Si$ (M+H) = 431.2842, found = 431.2841.

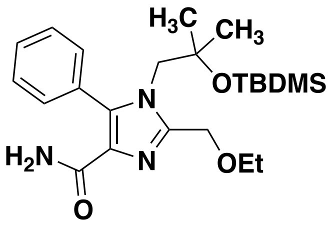

1-{2-[(tert-butyldimethylsilyl)oxy]-2-methylpropyl}-2-(ethoxymethyl)-5-phenyl-1H-imidazole-4-carboxamide (12)

To a solution of 1-{2-[(*tert*-butyldimethylsilyl)oxy]-2-methylpropyl}-2-(ethoxymethyl)-5-phenyl-1H-imidazole-4-carbonitrile (0.100 g, 0.242 mmol) in ethanol (3 mL) was added a solution of potassium hydroxide (0.135 g, 2.42 mmol) in water (3 mL), and the resulting mixture was heated at reflux for 18 hours. After this time, the mixture was allowed to cool to room temperature, added hydrochloric acid (0.2 mL, 12.1 M), then saturated aqueous sodium bicarbonate was added until neutral, and the resulting mixture was then extracted with ethyl acetate (3 × 20 mL). The combined organic layers were washed with saturated aqueous sodium chloride, dried over magnesium sulfate, filtered and concentrated to dryness. The resulting solid was triturated with a diethyl ether/hexanes mixture to give 1-{2-[(tert-butyldimethylsilyl)oxy]-2-methylpropyl}-2-(ethoxymethyl)-5-phenyl-1H-imidazole-4-carboxamide **(12)** (0.085 g, 82%) as an off-white solid; ^{1}H NMR (400 MHz, DMSO-d_6) δ 7.44-7.38 (m, 5H), 7.26 (s, 1H), 6.96 (s, 1H), 4.63 (s, 2H), 4.07 (s, 2H), 3.52 (q, J = 7.0 Hz, 2H), 1.15 (t, J = 7.0 Hz, 3H), 0.81 (s, 6H), 0.73 (s, 9H), -0.03 (s, 6H); ^{13}C NMR (101 MHz DMSO-d_6) δ 164.09, 145.29, 135.40, 131.22, 130.95, 130.04, 128.15, 127.76, 73.65, 65.34, 64.86, 54.05, 28.26, 25.81, 17.71, 15.01, -2.20; MS (ESI$^+$), calcd $C_{23}H_{38}N_3O_3Si$ (M+H) = 432.2682, found = 432.2688.

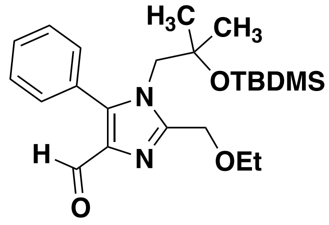

1-{2-[(tert-butyldimethylsilyl)oxy]-2-methylpropyl}-2-(ethoxymethyl)-5-phenyl-1H-imidazole-4-carbaldehyde (13)

A solution of 1-{2-[(*tert*-butyldimethylsilyl)oxy]-2-methylpropyl}-2-(ethoxymethyl)-5-phenyl-1H-imidazole-4-carbonitrile (1.70 g, 4.11 mmol) was cooled in a dry ice/isopropanol bath (~ -78 °C). Diisobutylaluminum hydride (8.2 mL, 8.2 mmol, 1 M in hexanes) was slowly added over 10 min. The solution was allowed to stir in the dry ice bath for 3.5 hours. While still in the dry ice bath, saturated aqueous ammonium chloride (100 mL) was added, the mixture was allowed to warm to room temperature and was extracted with ethyl acetate (3 × 75 mL). The combined organic layers were washed with saturated aqueous potassium sodium tartrate (150 mL), washed with saturated aqueous sodium chloride (150 mL), dried over magnesium sulfate, filtered and concentrated to dryness. The residue obtained was purified via flash SiO_2 chromatography (100 g silica gel, gradient of hexanes to 50% ethyl acetate/50% hexanes) to give 1-{2-[(tert-butyldimethylsilyl)oxy]-2-methylpropyl}-2-(ethoxymethyl)-5-phenyl-1H-imidazole-4-carbaldehyde **(13)** (1.21 g, 71%) as an off-white solid; ^{1}H NMR (400 MHz, DMSO-d_6) δ 9.56 (s, 1H), 7.55-7.51 (m, 5H), 4.68 (s, 2H), 4.18 (s, 2H), 3.53 (q, J = 7.0 Hz, 2H), 1.16 (t, J = 7.0 Hz, 3H), 0.85 (s, 6H), 0.72 (s, 9H), -0.02 (s, 6H); ^{13}C NMR (101 MHz DMSO-d_6) δ 184.32, 148.29, 142.59, 136.24, 130.59, 129.36, 128.78, 127.95, 73.69, 65.43, 64.92, 54.18, 28.24, 25.76, 17.67, 14.94, -2.23; MS (ESI$^+$), calcd $C_{23}H_{37}N_2O_3Si$ (M+H) = 417.2574, found = 417.2570.

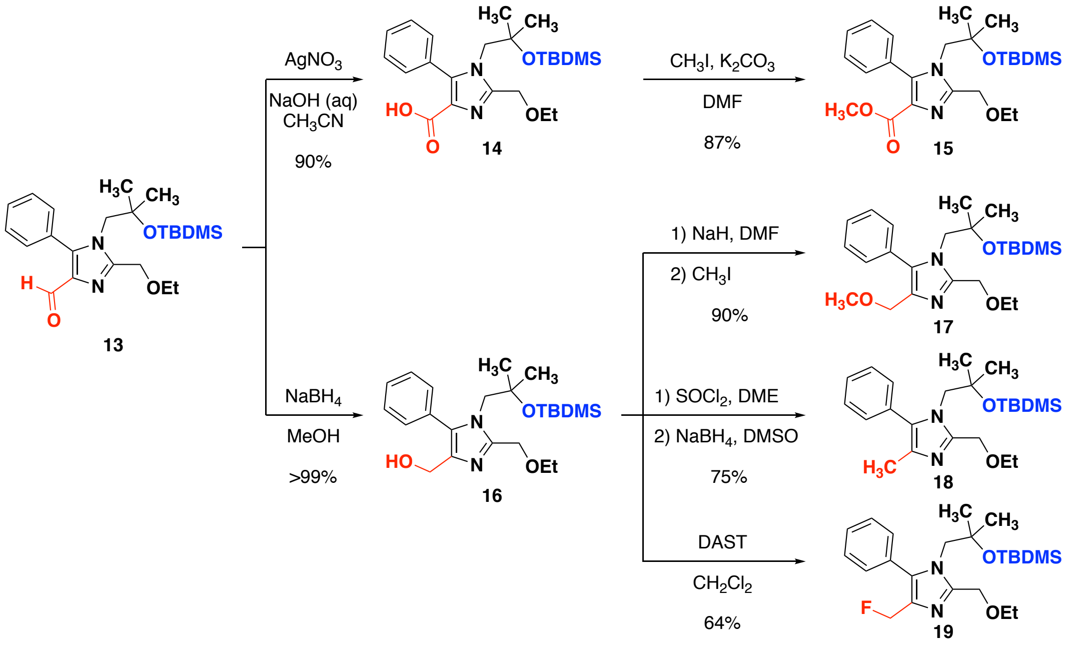

Supplementary Fig. 12 Carboxylic acid, ester, alcohol, ether, methyl, and fluoromethyl analog synthesis: Aldehyde **13** was used to synthesize carboxylic acid **14**, which was used to make methyl ester **15**. Aldehyde **13** was also used to make primary alcohol **16**, which was used to make methyl ether **17**, methyl **18**, and fluoromethyl **19**.

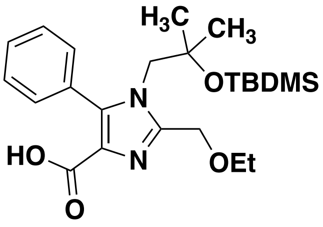

1-{2-[(tert-butyldimethylsilyl)oxy]-2-methylpropyl}-2-(ethoxymethyl)-5-phenyl-1H-imidazole-4-carboxylic acid (14)

To a solution of 1-{2-[(tert-butyldimethylsilyl)oxy]-2-methylpropyl}-2-(ethoxymethyl)-5-phenyl-1H-imidazole-4-carbaldehyde (0.537 g, 1.29 mmol) in acetonitrile (5.4 mL) was

added 10% aqueous sodium hydroxide (6.4 mL), followed by silver (I) nitrate (0.438 g, 2.58 mmol), and the resulting mixture was allowed to stir at room temperature for 24 hours. After this time, the mixture was neutralized with sodium phosphate monobasic monohydrate (80 mL, 1 M in water), diluted with saturated aqueous sodium chloride (50 mL), and the resulting mixture was extracted with ethyl acetate (4 × 50 mL). The combined organic layers were washed with saturated aqueous sodium chloride (100 mL), dried over magnesium sulfate, filtered and concentrated to dryness to give 1-{2-[(tert-butyldimethylsilyl)oxy]-2-methylpropyl}-2-(ethoxymethyl)-5-phenyl-1H-imidazole-4-carboxylic acid **(14)** (0.490 g, 88%) as a white solid; ^{1}H NMR (400 MHz, DMSO-d_6) δ 12.0 (br s, 1H), 7.47-7.38 (m, 5H), 4.64 (s, 2H), 4.06 (s, 2H), 3.50 (q, J = 7.0 Hz, 2H), 1.15 (t, J = 7.0 Hz, 3H), 0.82 (s, 6H), 0.73 (s, 9H), -0.03 (s, 6H); ^{13}C NMR (101 MHz DMSO-d_6) δ 163.78, 146.21, 138.53, 131.04, 129.88, 128.70, 128.48, 127.95, 73.61, 65.26, 64.94, 54.17, 28.29, 25.79, 17.70, 14.98, -2.21; MS (ESI$^+$), calcd $C_{23}H_{37}N_2O_4Si$ (M-H) = 431.2366, found = 431.2375.

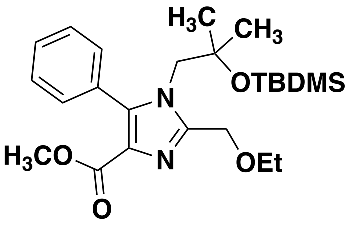

methyl 1-{2-[(tert-butyldimethylsilyl)oxy]-2-methylpropyl}-2-(ethoxymethyl)-5-phenyl-1H-imidazole-4-carboxylate (15)

To a mixture of potassium carbonate (0.363 g, 2.62 mmol), in *N*,*N*-dimethylformamide (8.5 mL) was added 1-{2-[(tert-butyldimethylsilyl)oxy]-2-methylpropyl}-2-(ethoxymethyl)-5-phenyl-1H-imidazole-4-carboxylic acid (0.283 g, 0.654 mmol), and the

resulting mixture was allowed to stir for 15 min. After this time, iodomethane (61 µL, 0.98 mmol) was added and allowed to stir for 17 hours. After this time, water (20 mL) was added and the resulting mixture was extracted with ethyl acetate (3 × 10 mL). The combined organic layers were washed with 5% aqueous lithium chloride (3 × 30 mL), washed with saturated aqueous sodium chloride (30 mL), dried over magnesium sulfate, filtered and concentrated to dryness. The residue obtained was purified via flash SiO_2 chromatography (10 g silica gel, gradient of hexanes to 35% ethyl acetate/65% hexanes) to give methyl 1-{2-[(tert-butyldimethylsilyl)oxy]-2-methylpropyl}-2-(ethoxymethyl)-5-phenyl-1H-imidazole-4-carboxylate **(15)** (0.254 g, 87%) as a colorless oil; ^{1}H NMR (400 MHz, DMSO-d_6) δ 7.49-7.43 (m, 3H), 7.43-7.39 (m, 2H), 4.64 (s, 2H), 4.08 (s, 2H), 3.59 (s, 3H), 3.50 (q, J = 7.0 Hz, 2H), 1.14 (t, J = 7.0 Hz, 3H), 0.83 (s, 6H), 0.72 (s, 9H), -0.03 (s, 6H); ^{13}C NMR (101 MHz DMSO-d_6) δ 162.84, 146.60, 139.09, 130.92, 129.49, 128.66, 128.02, 127.81, 73.60, 65.25, 64.91, 54.20, 50.74, 28.26, 25.77, 17.67, 14.94, -2.22; MS (ESI$^+$), calcd $C_{24}H_{39}N_2O_4Si$ (M+H) = 447.2679, found = 447.2677.

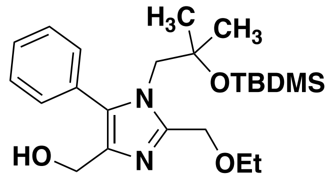

(1-{2-[(tert-butyldimethylsilyl)oxy]-2-methylpropyl}-2-(ethoxymethyl)-5-phenyl-1H-imidazol-4-yl)methanol (16)

To a solution of 1-{2-[(tert-butyldimethylsilyl)oxy]-2-methylpropyl}-2-(ethoxymethyl)-5-phenyl-1H-imidazole-4-carbaldehyde (0.458 g, 1.10 mmol) in methanol (17 mL) was added sodium borohydride (0.064 g, 1.7 mmol). The mixture was allowed to stir at room

temperature for 3 hours, and then saturated aqueous ammonium chloride (50 mL) was added. The mixture was extracted with dichloromethane (4 × 25 mL). The combined organic layers were washed with saturated aqueous sodium chloride (100 mL), dried over sodium sulfate, filtered and concentrated to dryness. The residue obtained was purified via flash SiO_2 chromatography (50 g silica gel, gradient of dichloromethane to 7% methanol/93% dichloromethane) to give (1-{2-[(tert-butyldimethylsilyl)oxy]-2-methylpropyl}-2-(ethoxymethyl)-5-phenyl-1H-imidazol-4-yl)methanol **(16)** (0.413 g, 90%) as a white solid; ^{1}H NMR (400 MHz, DMSO-d_6) δ 7.48-7.45 (m, 2H), 7.41–7.36 (m, 3H), 4.78 (t, J = 5.4, 1H), 4.60 (s, 2H), 4.17 (d, J = 5.2, 2H), 4.11 (s, 2H), 3.50 (q, J = 7.0 Hz, 2H), 1.14 (t, J = 7.0 Hz, 3H), 0.81 (s, 6H), 0.74 (s, 9H), 0.04 (s, 6H); ^{13}C NMR (101 MHz DMSO-d_6) δ 145.30, 138.16, 130.81, 130.61, 129.93, 128.61, 127.61, 74.00, 65.14, 65.08, 56.09, 54.24, 28.15, 25.82, 17.71, 15.01, -2.19.

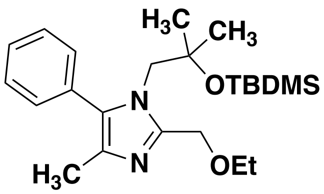

1-{2-[(tert-butyldimethylsilyl)oxy]-2-methylpropyl}-2-(ethoxymethyl)-4-methyl-5-phenyl-1H-imidazole (18)

A solution of (1-{2-[(tert-butyldimethylsilyl)oxy]-2-methylpropyl}-2-(ethoxymethyl)-5-phenyl-1H-imidazol-4-yl)methanol (0.050 g, 0.12 mmol) in 1,2-dichloroethane (2 mL) was cooled in an ice bath. Thionyl chloride (18 µL, 0.24 mmol) was added and the solution was allowed to stir in the ice bath for an additional 10 min. After this time, the solution was allowed to warm to room temperature and then heated at 60 °C for 2.5 hours.

After this time, the solution was allowed to cool to room temperature, diluted with dichloromethane (10 mL). The resulting organic solution was neutralized with saturated aqueous sodium bicarbonate (10 mL), washed with saturated aqueous sodium chloride (30 mL), dried over sodium sulfate, filtered and concentrated to dryness. The resulting residue was further dried on a high-vacuum pump for 1 hour. The resulting residue was dissolved in dimethyl sulfoxide (2 mL). Sodium borohydride (0.042 g, 1.1 mmol) was added. Additional dimethyl sulfoxide (1 mL) was immediately used to rinse the sides of the reaction flask. The mixture was allowed to stir at room temperature for 24 hours. After this time, water was added (10 mL) and the resulting mixture was extracted with diethyl ether (3 × 10 mL). The combined organic layers were washed with saturated aqueous sodium chloride (30 mL), dried over magnesium sulfate, filtered and concentrated to dryness. The resulting residue was purified via flash SiO_2 chromatography (10 g silica gel, gradient of hexanes to 14% ethyl acetate/86% hexanes) to give 1-{2-[(tert-butyldimethylsilyl)oxy]-2-methylpropyl}-2-(ethoxymethyl)-4-methyl-5-phenyl-1H-imidazole **(18)** (0.033 g, 75%) as a white solid; ^{1}H NMR (400 MHz, DMSO-d_6) δ 7.55-7.51 (m, 2H), 7.49-7.42 (m, 3H), 4.93 (s, 2H), 4.18 (s, 2H), 3.55 (q, J = 7.0 Hz), 2.13 (s, 3H), 1.16 (t, J = 7.0 Hz, 3H), 0.86 (s, 6H), 0.71 (s, 9H), -0.04 (s, 6H); ^{13}C NMR (101 MHz DMSO-d_6) δ 144.11, 131.26, 130.43, 129.21, 129.03, 128.77, 128.57, 73.36, 65.75, 60.67, 54.59, 28.26, 25.73, 17.64, 14.96, 10.92, -2.29; MS (ESI$^+$), calcd $C_{23}H_{39}N_2O_2Si$ (M+H) = 403.2781, found = 403.2779.

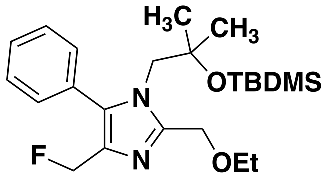

1-{2-[(tert-butyldimethylsilyl)oxy]-2-methylpropyl}-2-(ethoxymethyl)-4-(fluoromethyl)-5-phenyl-1H-imidazole (19)

A solution of (1-{2-[(tert-butyldimethylsilyl)oxy]-2-methylpropyl}-2-(ethoxymethyl)-5-phenyl-1H-imidazol-4-yl)methanol (0.113 g, 0.270 mmol) in dichloromethane (6 mL) was cooled in an ice bath. (Diethylamino)sulfur trifluoride (0.35 mL, 0.35 mmol, 1 M solution in dichloromethane) was added and the solution was allowed to stir in the ice bath for 1 hour. After this time, saturated aqueous sodium bicarbonate (10 mL) was added and the resulting mixture was extracted with dichloromethane (3 × 10 mL). The combined organic layers were washed with saturated aqueous sodium chloride (30 mL), dried over sodium sulfate, filtered and concentrated to dryness. The residue obtained was purified via flash SiO_2 chromatography (10 g silica gel, gradient of hexanes to 30% ethyl acetate/70% hexanes) to give 1-{2-[(tert-butyldimethylsilyl)oxy]-2-methylpropyl}-2-(ethoxymethyl)-4-(fluoromethyl)-5-phenyl-1H-imidazole **(19)** (0.073 g, 64%) as an off-white solid; ^{1}H NMR (400 MHz, DMSO-d_6) δ 7.54–7.50 (m, 2H), 7.47-7.40 (m, 3H), 5.08 (d, J = 50.0 Hz, 2H), 4.62 (s, 2H), 4.15 (s, 2H), 3.50 (q, J = 7.0 Hz, 2H), 1.15 (t, J = 7.0 Hz, 3H), 0.84 (s, 6H), 0.72 (s, 9H), -0.05 (s, 6H); ^{13}C NMR (101 MHz DMSO-d_6) δ 146.40, 134.22, 134.16, 132.88, 132.70, 129.75, 129.73, 129.70, 128.93, 128.33, 78.38, 76.80, 73.76, 65.23, 64.97, 54.36, 28.21, 25.76, 17.67, 14.97, -2.25; MS (ESI^{+}), calcd $C_{23}H_{38}FN_2O_2Si$ (M+H) = 421.2686, found = 421.2696.

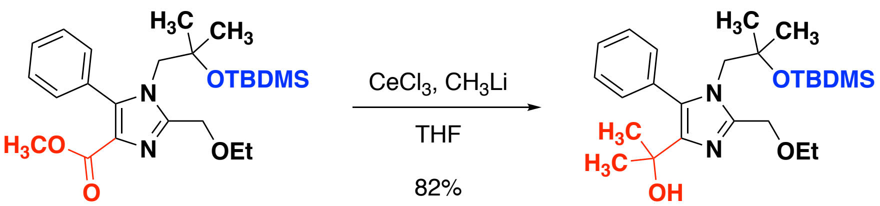

15 20

Supplementary Fig. 13 Tertiary alcohol analog synthesis: Methyl ester **15** was used to make tertiary alcohol **20**.

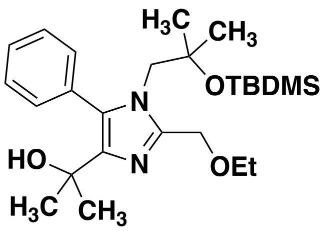

2-(1-{2-[(tert-butyldimethylsilyl)oxy]-2-methylpropyl}-2-(ethoxymethyl)-5-phenyl-1H-imidazol-4-yl)propan-2-ol (20)

In an oven-dried flask, cerium (III) chloride (0.054 g, 0.22 mmol) was taken up into tetrahydrofuran (2 mL) and stirred at room temperature for 2.5 hours. After this time, the mixture was cooled in a dry ice/isopropyl alcohol bath (~ -78 °C). Methyl lithium (0.24 mL, 0.39 mmol, 1.6 M solution in diethyl ether) and was allowed to stir in the dry ice bath for 30 min. In a second oven-dried flask solution of 1-{2-[(tert-butyldimethylsilyl)oxy]-2-methylpropyl}-2-(ethoxymethyl)-5-phenyl-1H-imidazole-4-carboxylate (0.051 g, 0.11 mmol) in tetrahydrofuran (1 mL) was prepared, cannulated into the first, cooled flask and stirred in the dry ice bath for 30 min. The mixture was removed from the dry ice bath and allowed to stir at room temperature for 17 hours. After this time, saturated aqueous ammonium chloride (10 mL) was added and the resulting mixture was extracted with ethyl acetate (3 × 5 mL). The combined organic layers were

washed with saturated aqueous sodium chloride (10 mL), dried over magnesium sulfate, filtered and concentrated to dryness. The residue obtained was purified via flash SiO_2 chromatography (10 g silica gel, gradient of dichloromethane to 80% ethyl acetate/20% dichloromethane) to give 2-(1-{2-[(tert-butyldimethylsilyl)oxy]-2-methylpropyl}-2-(ethoxymethyl)-5-phenyl-1H-imidazol-4-yl)propan-2-ol **(20)** (0.037 g, 76%) as a white solid; ^{1}H NMR (400 MHz, DMSO-d_6) δ 7.43–7.33 (m, 5H), 4.57 (s, 2H), 4.40 (s, 1H), 3.92 (s, 2H), 3.50 (q, J = 7.0 Hz, 2H), 1.27 (s, 6H), 1.14 (t, J = 7.0 Hz, 3H), 0.82 (s, 6H), 0.74 (s, 9H), -0.04 (s, 6H); ^{13}C NMR (101 MHz DMSO-d_6) δ 144.20, 143.26, 132.16, 131.97, 127.73, 127.65, 127.39, 73.66, 69.17, 65.22, 65.16, 53.88, 31.32, 28.46, 25.83, 17.73, 15.05, -2.17; MS (ESI^+), calcd $C_{25}H_{43}N_2O_3Si$ (M+H) = 447.3043, found = 447.3035.

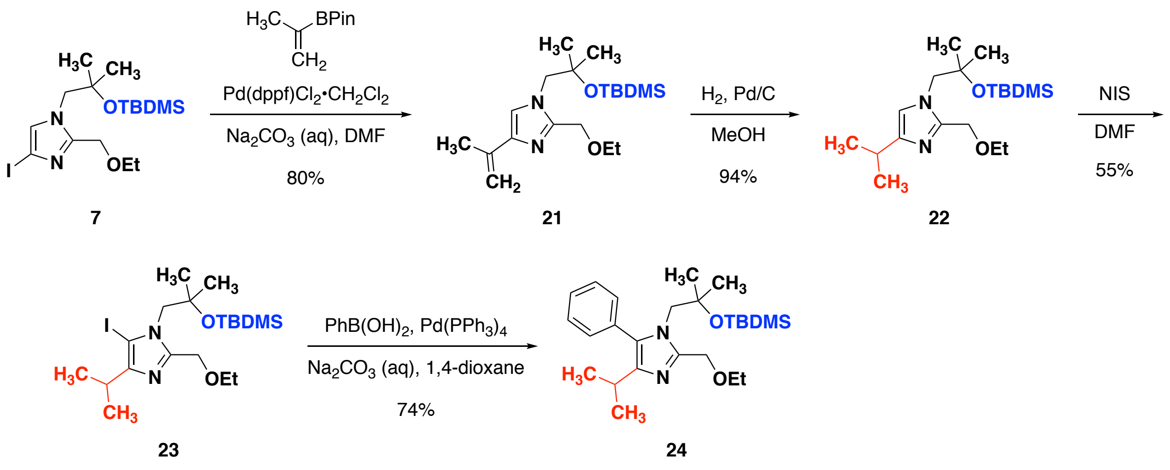

Supplementary Fig. 14 *iso*-Propyl analog synthesis: Iso-propyl **24** was made from iodo **7** in 31% yield over four steps.

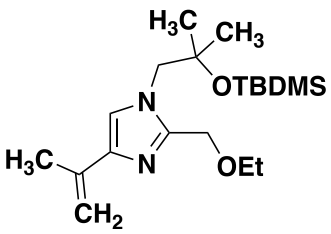

1-{2-[(tert-butyldimethylsilyl)oxy]-2-methylpropyl}-2-(ethoxymethyl)-4-(prop-1-en-2-yl)-1H-imidazole (21)

A mixture of 1-{2-[(*tert*-butyldimethylsilyl)oxy]-2-methylpropyl}-2-(ethoxymethyl)-4-iodo-1H-imidazole (0.300 g, 0.684 mmol), and aqueous sodium carbonate (2.5 mL, 2 M) in *N*,*N*-dimethylformamide (5 mL) was degassed and backfilled with nitrogen gas (4 ×). (1,1'-Bis(diphenylphosphino)ferrocene)palladium(II) dichloride•dichloromethane (0.028 g, 0.034 mmol) was added and the resulting mixture was degassed and backfilled with nitrogen gas (4 ×) again. Isopropenylboronic acid pinacol ester (0.14 mL, 0.75 mmol) was added and the resulting mixture was heated at 65 °C for 17 hours. After this time, the mixture was allowed to cool to room temperature, diluted with water (30 mL), and extracted with ethyl acetate (3 × 20 mL). The combined organic layers were washed with 5% aqueous lithium chloride (2 × 40 mL), washed with saturated aqueous sodium chloride (50 mL), dried over magnesium sulfate, filtered and concentrated to dryness. The residue obtained was purified via flash SiO_2 chromatography (25 g silica gel, gradient of hexanes to 30% ethyl acetate/70% hexanes) to give 1-{2-[(tert-butyldimethylsilyl)oxy]-2-methylpropyl}-2-(ethoxymethyl)-4-(prop-1-en-2-yl)-1H-imidazole **(21)** (0.193 g, 80%) as a yellow oil; ^{1}H NMR (400 MHz, DMSO-d_6) δ 7.14 (s, 1H), 5.48 (dd, J = 2.8, 0.57 Hz, 1H), 4.77 (dd, J = 2.8, 1.5 Hz, 1H), 4.45 (s, 2H), 3.89 (s, 2H), 3.44 (q, J = 7.0 Hz, 2H), 1.93 (s, 3H), 1.19 (s, 6H), 1.09 (t, J = 7.0 Hz, 3H), 0.86 (s, 9H), 0.06 (s, 6H); ^{13}C NMR

(101 MHz DMSO-d_6) δ 144.92, 139.48, 136.06, 118.69, 108.09, 73.63, 64.89, 63.78, 56.65, 27.01, 25.77, 19.95, 17.74, 14.95, -2.22.

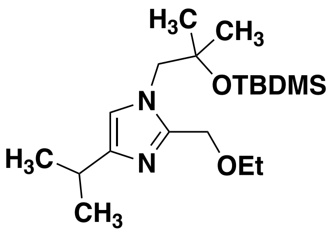

1-{2-[(tert-butyldimethylsilyl)oxy]-2-methylpropyl}-2-(ethoxymethyl)-4-(propan-2-yl)-1H-imidazole (22)

To a solution of 1-{2-[(tert-butyldimethylsilyl)oxy]-2-methylpropyl}-2-(ethoxymethyl)-4-(prop-1-en-2-yl)-1H-imidazole (0.190 g, 0.539 mmol) in methanol (9 mL) was added palladium on carbon (0.010 g, 5% basis). The mixture was degassed and backfilled with nitrogen gas (4 ×), then degassed and backfilled with hydrogen gas (4 ×). The reaction was then allowed to stir under hydrogen atmosphere (balloon) for 17 hours. After this time, the mixture was degassed and backfilled with nitrogen gas (4 ×), diluted with dichloromethane (20 mL), and filtered through Celite (rinsing with additional dichloromethane). The filtrate was concentrated to dryness. The residue obtained was purified via flash SiO_2 chromatography (10 g silica gel, gradient of hexanes to 50% ethyl acetate/40% hexanes) to give 1-{2-[(tert-butyldimethylsilyl)oxy]-2-methylpropyl}-2-(ethoxymethyl)-4-(propan-2-yl)-1H-imidazole **(22)** (0.180 g, 94%) as a colorless oil; ^{1}H NMR (400 MHz, DMSO-d_6) δ 6.81 (d, J = 0.7 Hz, 1H), 4.40 (s, 2H), 3.83 (s, 2H), 3.42 (q, J = 7.0 Hz, 2H), 2.70 (dsep, J = 6.9, 0.7 Hz, 1H), 1.17 (s, 6H), 1.13 (d, J = 6.9 Hz, 6H), 1.08 (t, J = 7.0 Hz, 3H), 0.85 (s, 9H), 0.05 (s, 6H) ; ^{13}C NMR (101 MHz DMSO-d_6) δ

145.53, 143.67, 115.95, 73.63, 64.79, 63.87, 56.52, 27.22, 27.09, 25.77, 22.38, 17.72, 14.95, -2.24.

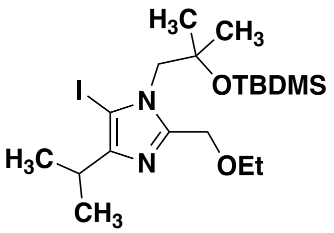

1-{2-[(tert-butyldimethylsilyl)oxy]-2-methylpropyl}-2-(ethoxymethyl)-5-iodo-4-(propan-2-yl)-1H-imidazole (23)

To a solution of 1-{2-[(tert-butyldimethylsilyl)oxy]-2-methylpropyl}-2-(ethoxymethyl)-4-(propan-2-yl)-1H-imidazole (0.175 g, 0.493 mmol) in *N*,*N*-dimethylformamide (5 mL) was added *N*-iodosuccinimide (0.166 g, 0.740 mmol) and the resulting solution was heated at 80 °C for 17 hours. After this time, the solution was allowed to cool to room temperature, diluted with water (30 mL), and solid sodium thiosulfate pentahydrate was added until no further color change was observed. The resulting mixture was extracted with ethyl acetate (3 × 20 mL). The combined organic layers were washed with 5% aqueous lithium chloride (3 × 40 mL), washed with saturated aqueous sodium chloride (50 mL), dried over magnesium sulfate, filtered and concentrated to dryness. The residue obtained was purified via flash SiO_2 chromatography (10 g silica gel, gradient of hexanes to 20% ethyl acetate/80% hexanes) to give 1-{2-[(tert-butyldimethylsilyl)oxy]-2-methylpropyl}-2-(ethoxymethyl)-5-iodo-4-(propan-2-yl)-1H-imidazole **(23)** (0.130 g, 55%) as a colorless oil; ^{1}H NMR (400 MHz, DMSO-d_6) δ 4.57 (s, 2H), 3.97 (s, 2H), 3.43 (q, J = 7.0 Hz, 2H), 2.81 (sep, J = 6.9 Hz, 1H), 1.30 (s, 6H), 1.12 (d, J = 6.9 Hz, 6H),

1.10 (t, J = 7.0 Hz, 3H), 0.74 (s, 9H), 0.05 (s, 6H); ^{13}C NMR (101 MHz DMSO-d_6) δ 149.15, 147.72, 74.05, 73.65, 65.31, 65.07, 56.16, 29.05, 27.45, 25.92, 22.27, 17.77, 14.96, –2.05; MS (ESI$^+$), calcd $C_{19}H_{38}IN_2O_2Si$ (M+H) = 481.1747, found = 481.1745.

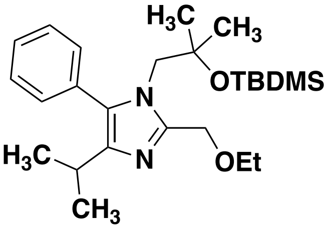

1-{2-[(tert-butyldimethylsilyl)oxy]-2-methylpropyl}-2-(ethoxymethyl)-5-phenyl-4-(propan-2-yl)-1H-imidazole (24)

A mixture of 1-{2-[(tert-butyldimethylsilyl)oxy]-2-methylpropyl}-2-(ethoxymethyl)-5-iodo-4-(propan-2-yl)-1H-imidazole (0.120 g, 0.250 mmol), phenylboronic acid (0.034 g, 0.28 mmol), and aqueous sodium carbonate (1.7 mL, 1 M) in 1,4-dioxane (3.4 mL) was degassed and backfilled with nitrogen gas (4×). Tetrakis(triphenylphosphine)palladium(0) (0.015 g, 0.013 mmol) was added, the mixture was degassed and backfilled with nitrogen gas (4×) again, and the mixture was heated at 80 °C for 18 hours. After this time, the mixture was allowed to cool to room temperature, diluted with water (10 mL), and the resulting mixture was extracted with ethyl acetate (3 × 5 mL). The combined organic layers were washed with saturated aqueous sodium chloride (10 mL), dried over magnesium sulfate, filtered and concentrated to dryness. The residue obtained was purified via flash SiO_2 chromatography (10 g silica gel, gradient of hexanes to 25% ethyl acetate/75% hexanes) to give 1-{2-[(tert-butyldimethylsilyl)oxy]-2-methylpropyl}-2-(ethoxymethyl)-5-phenyl-4-(propan-2-yl)-1H-imidazole **(24)** (0.080 g, 74%) as a colorless

oil; ^{1}H NMR (400 MHz, DMSO-d_6) δ 7.49–7.45 (m, 2H), 7.38–7.34 (m, 1H), 4.56 (s, 2H), 4.03 (s, 2H), 3.50 (q, J = 7.0 Hz, 2H), 2.76 (sep, J = 6.8 Hz, 1H), 1.15 (t, J = 7.0 Hz, 3H), 1.11 (d, J = 6.2 Hz, 6H), 0.82 (s, 6H), 0.70 (s, 9H), –0.06 (s, 6H); ^{13}C NMR (101 MHz DMSO-d_6) δ 145.31, 143.19, 131.37, 129.92, 128.74, 127.35, 127.03, 73.62, 65.28, 65.20, 54.05, 28.43, 25.78, 25.43, 23.17, 17.67, 15.01, –2.26; MS (ESI$^+$), calcd $C_{25}H_{43}N_2O_2Si$ (M+H) = 431.3094, found = 431.3090.

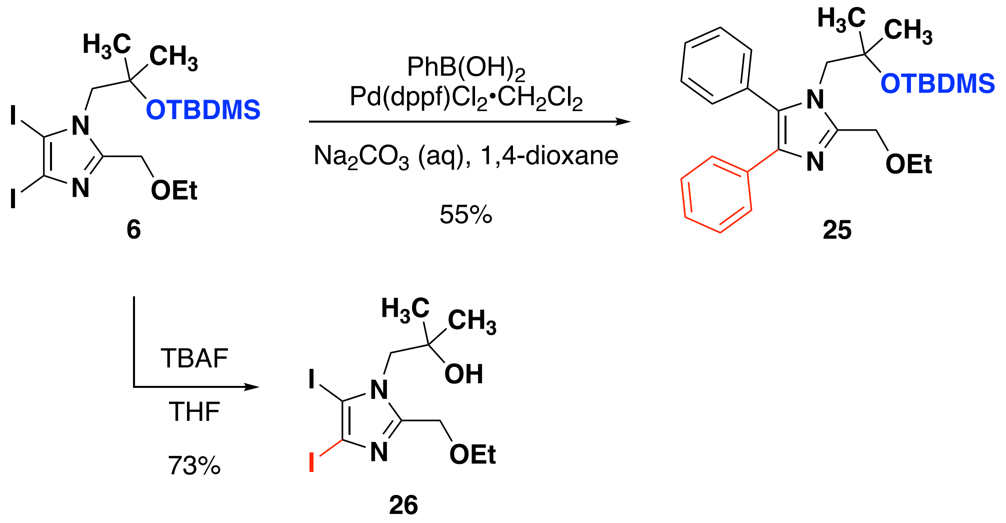

Supplementary Fig. 15 4,5-Diphenyl and 4,5-diiodo analog synthesis: Diphenyl intermediate **25** and final diiodo target **26** were made from diiodo intermediate **6**.

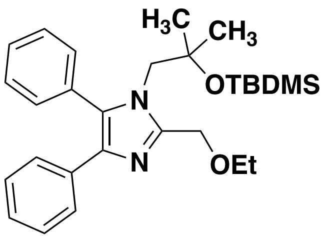

1-{2-[(tert-butyldimethylsilyl)oxy]-2-methylpropyl}-2-(ethoxymethyl)-4,5-diphenyl-1H-imidazole (25)

A mixture of 1-{2-[(tert-butyldimethylsilyl)oxy]-2-methylpropyl}-2-(ethoxymethyl)-4,5-diiodo-1H-imidazole (0.100 g, 0.177 mmol), phenylboronic acid (0.065 g, 0.53 mmol), and aqueous sodium carbonate (0.55 mL, 1.1 mmol, 2 M) in 1,4-dioxane (4 mL) was degassed and backfilled with nitrogen gas (4×). (1,1'-Bis(diphenylphosphino)ferrocene)palladium(II) dichloride•dichloromethane (0.015 g, 0.018 mmol) was added and the resulting mixture was degassed and backfilled with nitrogen gas (4x) again. The reaction mixture was then heated at 100 °C for 17 hours. After this time, the reaction mixture was diluted with water (10 mL) and extracted with ethyl acetate (3 × 10 mL). The combined organic layers were washed with saturated aqueous sodium chloride (20 mL), dried over magnesium sulfate, filtered and concentrated to dryness. The residue obtained was purified via flash SiO_2 chromatography (10 g silica gel, gradient of hexanes to 30% ethyl acetate/70% hexanes) to give 1-{2-[(tert-butyldimethylsilyl)oxy]-2-methylpropyl}-2-(ethoxymethyl)-4,5-diphenyl-1H-imidazole **(25)** (0.045 g, 55%) as a yellow oil;

^{1}H NMR (400 MHz, DMSO-d_6) δ 7.50–7.41 (m, 3H), 7.36–7.33 (m, 2H), 7.31–7.29 (m, 2H), 7.20–7.16 (m, 2H), 7.13–7.09 (m, 1H), 4.67 (s, 2H), 4.06 (s, 2H), 3.52 (q, J = 7.0 Hz, 2H), 1.15 (t, J = 7.0 Hz, 3H), 0.86 (s, 6H), 0.75 (s, 9H), –0.02 (s, 6H); ^{13}C NMR (101 MHz DMSO-d_6) δ 146.07, 136.00, 134.88, 131.19, 131.06, 129.40, 129.06, 128.45, 127.99, 126.53, 126.07, 73.64, 65.20, 65.09, 54.11, 28.48, 25.83, 17.76, 15.05, –2.16.

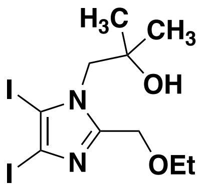

1-[2-(ethoxymethyl)-4,5-diiodo-1H-imidazol-1-yl]-2-methylpropan-2-ol (26)

To a solution of 1-{2-[(tert-butyldimethylsilyl)oxy]-2-methylpropyl}-2-(ethoxymethyl)-4,5-diiodo-1H-imidazole (0.100 g, 0.177 mmol) in tetrahydrofuran (4.5 mL) was added tetrabutylammonium fluoride (0.53 mL, 0.53 mmol, 1 M solution in tetrahydrofuran). The reaction was allowed to stir at room temperature for 16 hours. After this time, saturated aqueous ammonium chloride (10 mL) was added and the resulting mixture was extracted with ethyl acetate (3 × 5 mL). The combined organic layers were washed with saturated aqueous sodium chloride (20 mL), dried over magnesium sulfate, filtered and concentrated to dryness. The residue obtained was purified via flash SiO_2 chromatography (10 g silica gel, gradient of hexanes to 70% ethyl acetate/30% hexanes) to give 1-[2-(ethoxymethyl)-4,5-diiodo-1H-imidazol-1-yl]-2-methylpropan-2-ol **(26)** (0.058 g, 73%) as an off-white solid; ^{1}H NMR (400 MHz, DMSO-d_6) δ 4.88 (s, 1H), 4.65 (s, 2H), 4.03 (s, 2H), 3.41 (q, J = 7.0 Hz, 2H), 1.13 (s, 6H), 1.08 (t, J = 7.0 Hz, 3H); ^{13}C NMR (101 MHz DMSO-d_6) δ 150.77, 95.89, 89.07, 70.17, 65.08, 64.77, 57.36, 28.14, 14.98; MS (ESI$^+$), calcd $C_{10}H_{17}I_2N_2O_2$ (M+H) = 450.9379, found = 450.9389.

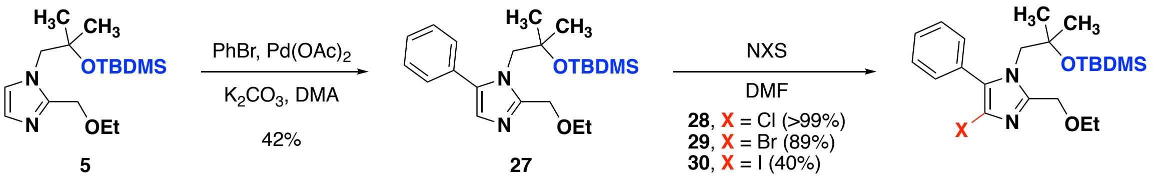

Supplementary Fig. 16 4-Chloro, 4-bromo, and 4-iodo analog synthesis: Halogenated compounds **28**, **29**, and **30** were made from imidazole intermediate **5**.

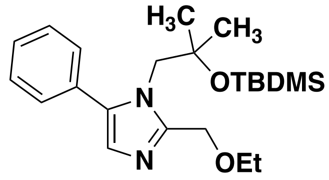

1-{2-[(tert-butyldimethylsilyl)oxy]-2-methylpropyl}-2-(ethoxymethyl)-5-phenyl-1H-imidazole (27)

A mixture of 1-{2-[(*tert*-butyldimethylsilyl)oxy]-2-methylpropyl}-2-(ethoxymethyl) 1H-imidazole (7.00 g, 22.4 mmol), bromobenzene (3.9 mL, 37 mmol), and potassium carbonate (10.2 g, 74.0 mmol) in *N*,*N*-dimethylacetamide (180 mL) was degassed and backfilled with nitrogen gas (4 ×). Palladium (II) acetate (0.831 g, 3.70 mmol) was added and the mixture was degassed and backfilled with nitrogen gas (4×) again. The mixture was heated at 150 °C for 21 hours, and after this time the reaction was allowed to cool to room temperature. Water (200 mL) was added and the resulting mixture was extracted with ethyl acetate (3 × 200 mL). The combined organic layers were washed with 5% aqueous lithium chloride (3 × 400 mL), washed with saturated aqueous sodium chloride (400 mL), dried over magnesium sulfate, filtered and concentrated to dryness. The residue obtained was purified via flash SiO_2 chromatography (340 g silica gel, gradient of dichloromethane to 4% methanol/96% dichloromethane) to give 1-{2-[(tert-butyldimethylsilyl)oxy]-2-methylpropyl}-2-(ethoxymethyl)-5-phenyl-1H-imidazole **(27)** (2.88 g, 20%) as a red oil; ^{1}H NMR (400 MHz, DMSO-d_6) δ 7.47-7.40 (m, 4H), 7.38-7.33 (m, 1H), 6.91 (s, 1H), 4.60 (s, 2H), 4.20 (s, 2H), 3.49 (q, *J* = 7.0 Hz, 2H), 1.14 (t, *J* = 7.0 Hz, 3H), 0.86 (s, 6H), 0.72 (s, 9H), -0.05 (s, 6H); ^{13}C NMR (101 MHz DMSO-d_6) δ 147.32, 133.86, 131.35, 128.88, 128.30, 127.49, 126.83, 73.90, 65.16, 65.04, 54.23, 28.21,

25.75, 17.66, 14.97, –2.26; MS (ESI$^+$), calcd $C_{22}H_{37}N_2O_2Si$ (M+H) = 389.2624, found = 389.2622.

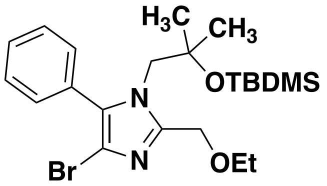

4-bromo-1-{2-[(tert-butyldimethylsilyl)oxy]-2-methylpropyl}-2-(ethoxymethyl)-5-phenyl-1H-imidazole (29)

To a solution of 1-{2-[(tert-butyldimethylsilyl)oxy]-2-methylpropyl}-2-(ethoxymethyl)-5-phenyl-1H-imidazole (0.300 g, 0.772 mmol) in *N*,*N*-dimethylformamide (11 mL) was added a solution of *N*-bromosuccinimide (0.144 g, 0.811 mmol) in *N*,*N*-dimethylformamide (4 mL), and the solution was allowed to stir for 18 hours. After this time, the solution was diluted with water (15 mL), and solid sodium thiosulfate pentahydrate was added until no further color change was observed, resulting in a pale yellow suspension. The resulting mixture was extracted with ethyl acetate (3 × 20 mL). The combined organic layers were washed with 5% aqueous lithium chloride (3 × 40 mL), washed with saturated aqueous sodium chloride (40 mL), dried over magnesium sulfate, filtered and concentrated to dryness. The residue obtained was purified via flash SiO_2 chromatography (25 g silica gel, gradient of dichloromethane to 5% methanol/95% dichloromethane) to give 4-bromo-1-{2-[(tert-butyldimethylsilyl)oxy]-2-methylpropyl}-2-(ethoxymethyl)-5-phenyl-1H-imidazole **(29)** (0.320 g, 89%) as an off white solid; ^{1}H NMR (400 MHz, DMSO-d_6) δ 7.52–7.48 (m, 2H), 7.45–7.40 (m, 3H), 4.56 (s, 2H), 4.13 (s, 2H), 3.50 (q, J = 7.0 Hz, 2H), 1.15 (t, J = 7.0 Hz, 3H), 0.84 (s, 6H), 0.72 (s, 9H), –0.04

(s, 6H); ^{13}C NMR (101 MHz DMSO-d_6) δ 146.45, 130.39, 130.09, 129.26, 128.79, 128.40, 113.50, 73.62, 65.25, 64.61, 54.90, 28.18, 25.75, 17.66, 14.94, –2.25; MS (ESI$^+$), calcd $C_{22}H_{36}BrN_2O_2Si$ (M+H) = 353.0866, found = 353.0868.

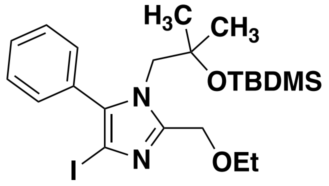

1-{2-[(tert-butyldimethylsilyl)oxy]-2-methylpropyl}-2-(ethoxymethyl)-4-iodo-5-phenyl-1H-imidazole (30)

To a solution of 1-{2-[(tert-butyldimethylsilyl)oxy]-2-methylpropyl}-2-(ethoxymethyl)-5-phenyl-1H-imidazole (0.178 g, 0.458 mmol) in *N*,*N*-dimethylformamide (5 mL) at 60 °C was added a solution of *N*-iodosuccinimide (0.113 g, 0.504 mmol) in *N*,*N*-dimethylformamide (1 mL) and the solution was allowed to stir 60 °C for 17 hours. After this time, water (10 mL) was added, solid sodium thiosulfate pentahydrate was added until no further color change was observed, and the resulting mixture was extracted with ethyl acetate (3 × 10 mL). The combined organic layers were washed with 5% aqueous lithium chloride (3 × 30 mL), washed with saturated aqueous sodium chloride (30 mL), dried over magnesium sulfate, filtered and concentrated to dryness. The residue obtained was purified via flash SiO_2 chromatography (10 g silica gel, gradient of hexanes to 20% ethyl acetate/80% hexanes) to give 1-{2-[(tert-butyldimethylsilyl)oxy]-2-methylpropyl}-2-(ethoxymethyl)-4-iodo-5-phenyl-1H-imidazole **(30)** (0.094 g, 40%) as a colorless oil; ^{1}H NMR (400 MHz, DMSO-d_6) δ 7.52–7.48 (m, 2H), 7.44–7.40 (m, 3H), 4.57 (s, 2H), 4.13 (s, 2H), 3.50 (q, *J* = 7.0 Hz, 2H), 1.15 (t, *J* = 7.0 Hz, 3H), 0.84 (s, 6H), 0.73 (s, 9H),

–0.03 (s, 6H); ^{13}C NMR (101 MHz DMSO-d_6) δ 146.46, 130.40, 130.10, 129.27, 128.80, 128.41, 113.50, 73.62, 65.26, 64.61, 54.90, 28.18, 25.76, 17.67, 14.95, –2.24; MS (ESI$^+$), calcd $C_{22}H_{36}IN_2O_2Si$ (M+H) = 515.1591, found = 515.1609.

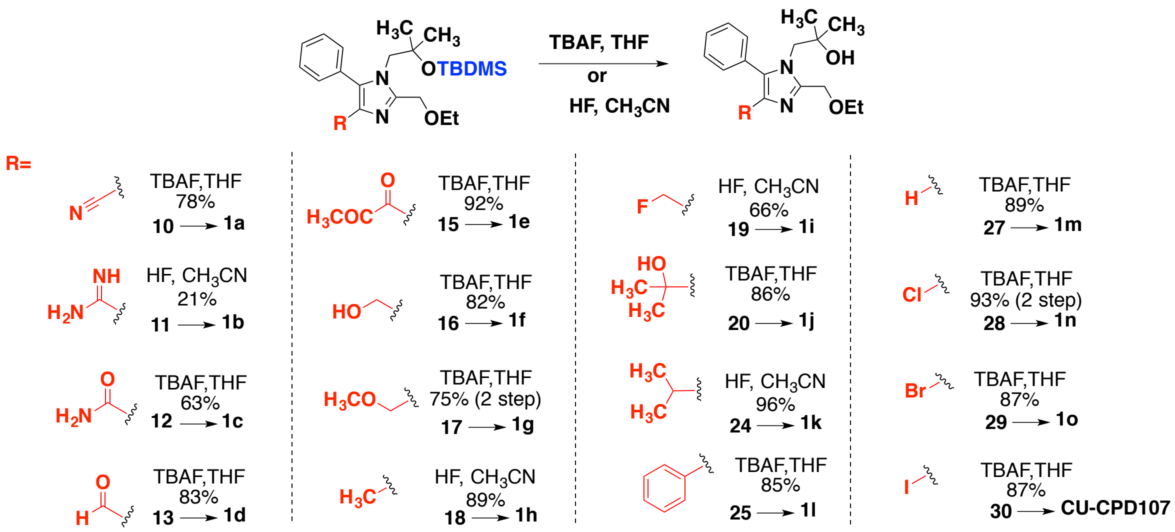

Supplementary Fig. 17 TBDMS deprotections: TBDMS deprotections were carried out with either TBAF or hydrofluoric acid to yield a variety of tertiary alcohols.

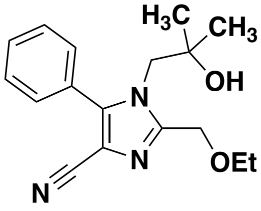

2-(ethoxymethyl)-1-(2-hydroxy-2-methylpropyl)-5-phenyl-1H-imidazole-4-carbonitrile (31, also called as 1a)

To a solution of 1-{2-[(*tert*-butyldimethylsilyl)oxy]-2-methylpropyl}-2-(ethoxymethyl)-5-phenyl-1H-imidazole-4-carbonitrile (0.050 g, 0.12 mmol) in tetrahydrofuran (2 mL) was

added tetrabutylammonium fluoride (0.36 mL, 0.36 mmol, 1 M solution in tetrahydrofuran). The solution was allowed to stir at room temperature for 18 hours. After this time, saturated aqueous ammonium chloride (5 mL) was added and the resulting mixture was extracted with ethyl acetate (3 × 5 mL). The combined organic layers were washed with saturated aqueous sodium chloride (10 mL), dried over magnesium sulfate, filtered and concentrated to dryness. The residue obtained was purified via flash SiO_2 chromatography (10 g silica gel, gradient of hexanes to 70% ethyl acetate/30% hexanes) to give 2-(ethoxymethyl)-1-(2-hydroxy-2-methylpropyl)-5-phenyl-1H-imidazole-4-carbonitrile **(31)** (0.028 g, 78%) as an off-white solid; ^{1}H NMR (400 MHz, DMSO-d_6) δ 7.59–7.50 (m, 5H), 4.80 (s, 1H), 4.72 (s, 2H), 4.13 (s, 2H), 3.52 (q, J = 7.0 Hz, 2H), 1.15 (t, J = 7.0 Hz, 3H), 0.75 (s, 6H); ^{13}C NMR (101 MHz DMSO-d_6) δ 149.06, 142.88, 129.58, 129.43, 129.13, 127.50, 115.87, 110.36, 69.65, 65.38, 64.47, 53.94, 27.57, 14.95; MS (ESI$^+$), calcd $C_{17}H_{22}N_3O_2$ (M+H) = 300.1712, found = 300.1717.

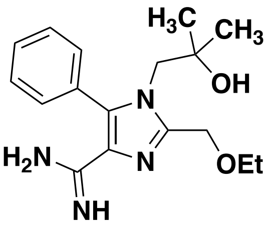

2-(ethoxymethyl)-1-(2-hydroxy-2-methylpropyl)-5-phenyl-1H-imidazole-4-carboximidamide (32, also called as 1b)

To a solution of 1-{2-[(tert-butyldimethylsilyl)oxy]-2-methylpropyl}-2-(ethoxymethyl)-5-phenyl-1H-imidazole-4-carboximidamide (0.098 g, 0.23 mmol) in acetonitrile (5 mL, in an HDPE scintillation vial) was added hydrofluoric acid (5 mL, 48 wt % in water) and the solution was allowed to stir at room temperature for 21 hours. After this time, the solution

was added to a mixture of sodium carbonate (50.0 g, 472 mmol) in acetonitrile (100 mL, cooled in an ice bath to ~ 0 °C) and stirred for 30 min. After this time, the solution was filtered and the filtrate was concentrated to dryness. The residue obtained was purified via flash SiO_2 chromatography (10 g silica gel, gradient of dichloromethane to 20%[1% triethylamine/99% methanol]/80% dichloromethane, performed 3×) to give 2-(ethoxymethyl)-1-(2-hydroxy-2-methylpropyl)-5-phenyl-1H-imidazole-4-carboximidamide **(32)** (0.015 g, 21%) as a white solid; ^{1}H NMR (400 MHz, DMSO-d_6) δ 8.44 (br s, 3H), 7.58–7.47 (m, 5H), 4.88 (s, 1H), 4.77 (s, 2H), 4.03 (s, 2H), 3.55 (q, J = 7.0 Hz, 2H), 1.16 (t, J = 7.0 Hz, 3H), 0.74 (s, 6H); ^{13}C NMR (101 MHz DMSO-d_6) δ 159.95, 148.05, 137.53, 130.92, 129.86, 129.15, 127.46, 125.27, 69.50, 65.49, 64.58, 53.57, 27.76, 15.05; MS (ESI$^+$), calcd $C_{17}H_{25}N_4O_2$ (M+H) = 317.1978, found = 317.1970.

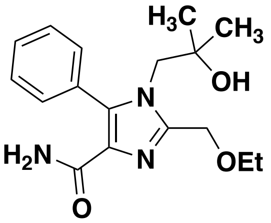

2-(ethoxymethyl)-1-(2-hydroxy-2-methylpropyl)-5-phenyl-1H-imidazole-4-carboxamide (33, also called as 1c)

To a solution of 1-{2-[(tert-butyldimethylsilyl)oxy]-2-methylpropyl}-2-(ethoxymethyl)-5-phenyl-1H-imidazole-4-carboxamide (0.041 g, 0.095 mmol) in tetrahydrofuran (4 mL) was added tetrabutylammonium fluoride (95 µL, 0.095 mmoL, 1 M solution in tetrahydrofuran), and the solution was allowed to stir at room temperature for 18 hours. After this time, saturated aqueous ammonium chloride (10 mL) was added and the resulting mixture was extracted with ethyl acetate (3 × 5 mL). The combined organic

layers were washed with saturated aqueous sodium chloride (10 mL), dried over magnesium sulfate, filtered and concentrated to dryness. The solid obtained was recrystallized from ethyl acetate and hexanes to give 2-(ethoxymethyl)-1-(2-hydroxy-2-methylpropyl)-5-phenyl-1H-imidazole-4-carboxamide **(33)** (0.019 g, 63%) as a white solid; ^{1}H NMR (400 MHz, DMSO-d_6) δ 7.43–7.34 (m, 5H), 7.24 (d, *J* = 2.1 Hz, 1H), 6.94 (d, *J* = 2.1 Hz, 1H), 4.75 (s, 1H), 4.71 (s, 2H), 3.98 (s, 2H), 3.52 (q, *J* = 7.0 Hz, 2H), 1.15 (t, *J* = 7.0 Hz, 3H), 0.71 (s, 6H); ^{13}C NMR (101 MHz DMSO-d_6) δ 164.15, 145.52, 135.59, 131.28, 130.77, 130.08, 128.03, 127.63, 69.61, 65.20, 64.76, 53.13, 27.77, 15.05; MS (ESI$^+$), calcd $C_{17}H_{24}N_3O_3$ (M+H) = 318.1818, found = 318.1825.

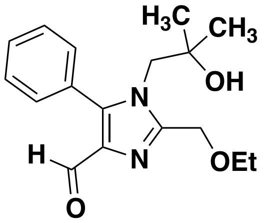

2-(ethoxymethyl)-1-(2-hydroxy-2-methylpropyl)-5-phenyl-1H-imidazole-4-carbaldehyde (34, also called as 1d)

To a solution of 1-{2-[(tert-butyldimethylsilyl)oxy]-2-methylpropyl}-2-(ethoxymethyl)-5-phenyl-1H-imidazole-4-carbaldehyde (0.075 g, 0.18 mmol) in tetrahydrofuran (6 mL) was added tetrabutylammonium fluoride (0.54 mL, 0.54 mmol, 1 M solution in tetrahydrofuran). The solution was allowed to stir at room temperature for 16 hours. After this time, saturated aqueous ammonium chloride (10 mL) was added and the resulting mixture was extracted with ethyl acetate (3 × 5 mL). The combined organic layers were washed with saturated aqueous sodium chloride (10 mL), dried over magnesium sulfate, filtered and concentrated to dryness. The residue obtained was purified via flash SiO_2

chromatography (10 g silica gel, gradient of dichloromethane to 7% methanol/93% dichloromethane) to give 2-(ethoxymethyl)-1-(2-hydroxy-2-methylpropyl)-5-phenyl-1H-imidazole-4-carbaldehyde **(34)** (0.083 g, 83%) as a white solid; ^{1}H NMR (400 MHz, DMSO-d_6) δ 9.53 (s, 1H), 7.57–7.47 (m, 5H), 4.80 (s, 1H), 4.76 (s, 2H), 4.09 (s, 2H), 3.53 (q, *J* = 7.0 Hz, 2H), 1.16 (t, *J* = 7.0 Hz, 3H), 0.75 (s, 6H); ^{13}C NMR (101 MHz DMSO-d_6) δ 145.30, 138.16, 130.81, 130.61, 129.93, 128.61, 127.61, 74.00, 65.14, 65.08, 56.09, 54.24, 28.15, 25.82, 17.71, 15.01, –2.19; MS (ESI$^+$), calcd $C_{17}H_{23}N_2O_3$ (M+H) = 303.1709, found = 303.1711.

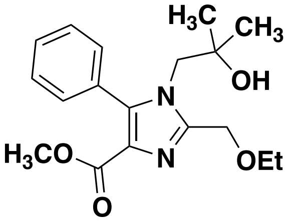

methyl 2-(ethoxymethyl)-1-(2-hydroxy-2-methylpropyl)-5-phenyl-1H-imidazole-4-carboxylate (35, also called as 1e)

To a solution of methyl 1-{2-[(tert-butyldimethylsilyl)oxy]-2-methylpropyl}-2-(ethoxymethyl)-5-phenyl-1H-imidazole-4-carboxylate (0.050 g, 0.11 mmol) in tetrahydrofuran (3 mL) was added tetrabutylammonium fluoride (0.33 mL, 0.33 mmol, 1 M solution in tetrahydrofuran), and the solution was allowed to stir at room temperature for 16 hours. After this time, saturated aqueous ammonium chloride (10 mL) was added and the resulting mixture was extracted with ethyl acetate (3 × 5 mL). The combined organic layers were washed with saturated aqueous sodium chloride, dried over magnesium sulfate, filtered and concentrated to dryness. The residue obtained was purified via flash SiO_2 chromatography (10 g silica gel, gradient of dichloromethane to 8%

methanol/92% dichloromethane) to give methyl 2-(ethoxymethyl)-1-(2-hydroxy-2-methylpropyl)-5-phenyl-1H-imidazole-4-carboxylate **(35)** (0.034 g, 92%) as an off-white solid; ^{1}H NMR (400 MHz, DMSO-d_6) δ 7.49–7.45 (m, 2H), 7.41–7.34 (m, 3H), 4.67 (2 x s, 3H), 4.09 (s, 2H), 4.03 (s, 2H), 3.50 (q, J = 7.0 Hz, 2H), 3.16 (s, 3H), 1.14 (t, J = 7.0 Hz, 3H), 0.71 (s, 6H); ^{13}C NMR (101 MHz DMSO-d_6) δ 145.78, 134.30, 132.45, 130.45, 130.03, 128.58, 127.79, 69.82, 66.77, 65.02, 64.87, 56.94, 53.39, 27.72, 15.06; MS (ESI^{+}), calcd $C_{18}H_{25}N_2O_4$ (M+H) = 319.2022, found = 319.2020.

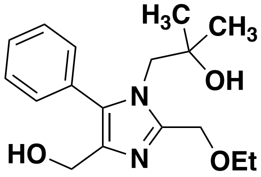

1-[2-(ethoxymethyl)-4-(hydroxymethyl)-5-phenyl-1H-imidazol-1-yl]-2-methylpropan-2-ol (36, also called as 1f)

To a solution of (1-{2-[(tert-butyldimethylsilyl)oxy]-2-methylpropyl}-2-(ethoxymethyl)-5-phenyl-1H-imidazol-4-yl)methanol (0.030 g, 0.072 mmol) in tetrahydrofuran (2 mL) was added tetrabutylammonium fluoride (0.22 mL, 0.22 mmol, 1 M solution in tetrahydrofuran). The solution was allowed to stir at room temperature for 16 hours. After this time, saturated aqueous ammonium chloride (10 mL) was added and the resulting mixture was extracted with ethyl acetate (3 × 5 mL). The combined organic layers were washed with saturated aqueous sodium chloride (10 mL), dried over magnesium sulfate, filtered and concentrated to dryness. The residue obtained was purified via flash SiO_2 chromatography (10 g silica gel, gradient of hexanes to 70% ethyl acetate/30% hexanes) to give 1-[2-(ethoxymethyl)-4-(hydroxymethyl)-5-phenyl-1H-imidazol-1-yl]-2-

methylpropan-2-ol **(36)** (0.018 g, 82%) as a white solid; ^{1}H NMR (400 MHz, DMSO-d_6) δ 7.48–7.44 (m, 2H), 7.39–7.35 (m, 3H), 4.74 (t, *J* = 5.4 Hz, 1H), 4.66 (s, 2H), 4.64 (s, 1H), 4.16 (d, *J* = 5.4 Hz, 2H), 4.03 (s, 2H), 3.51 (q, *J* = 7.0 Hz, 2H), 1.14 (t, *J* = 7.0 Hz, 3H), 0.71 (s, 6H); ^{13}C NMR (101 MHz DMSO-d_6) δ 145.53, 137.69, 130.86, 130.76, 130.09, 128.45, 127.52, 69.82, 65.01, 64.91, 56.15, 53.30, 27.72, 15.04; MS (ESI^{+}), calcd $C_{17}H_{25}N_2O_3$ (M+H) = 305.1865, found = 305.1873 .

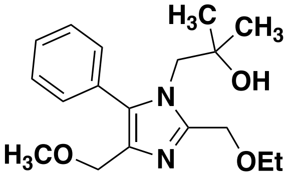

1-[2-(ethoxymethyl)-4-(methoxymethyl)-5-phenyl-1H-imidazol-1-yl]-2-methylpropan-2-ol (37, also called as 1g)

A solution of (1-{2-[(tert-butyldimethylsilyl)oxy]-2-methylpropyl}-2-(ethoxymethyl)-5-phenyl-1H-imidazol-4-yl)methanol (0.050 g, 0.12 mmol) in *N*,*N*-dimethylformamide (2 mL) was cooled in an ice bath (~ 0 °C). Sodium hydride (0.008 g, 0.2 mmol, 60 dispersion in mineral oil) was added and the resulting mixture was allowed to stir in the ice bath for 20 min. After this time, iodomethane (15 μL, 0.24 mmol) was added. The ice bath was removed and the solution was allowed to stir at room temperature for 19 hours. After this time, saturated aqueous ammonium chloride (10 mL) was added and the resulting mixture was extracted with ethyl acetate (3 × 5 mL). The combined organic layers were washed with 5% aqueous lithium chloride (3 × 10 mL), washed with saturated aqueous sodium chloride (10 mL), dried over magnesium sulfate, filtered and concentrated to dryness. The residue obtained was filtered through a plug of silica gel, (≈

10 g, eluting with ethyl acetate) and the filtrate was concentrated to dryness to give crude 1-{2-[(tert-butyldimethylsilyl)oxy]-2-methylpropyl}-2-(ethoxymethyl)-4-(methoxymethyl)-5-phenyl-1H-imidazole **(17)** (0.051 g, 98%) as a colorless oil, which was used in the next reaction without further purification. The colorless oil obtained (0.045 g, 0.10 mmol) was dissolved in tetrahydrofuran (3 mL), and tetrabutylammonium fluoride (0.30 mL, 0.30 mmol, 1 M solution in tetrahydrofuran) was added. The solution was allowed to stir for 18 hours at room temperature. After this time, saturated aqueous ammonium chloride (10 mL) was added and the resulting mixture was extracted with ethyl acetate (3 × 5 mL). The combined organic layers were washed with saturated aqueous sodium chloride (10 mL), dried over magnesium sulfate, filtered and concentrated to dryness. The residue obtained was purified via flash SiO_2 chromatography (10 g silica gel, gradient of hexanes to ethyl acetate to 10% methanol/90% ethyl acetate to give 1-[2-(ethoxymethyl)-4-(methoxymethyl)-5-phenyl-1H-imidazol-1-yl]-2-methylpropan-2-ol **(37)** (0.024 g, 75%) as a white solid; ^{1}H NMR (400 MHz, DMSO-d_6) δ 7.48–7.41 (m, 3H), 7.39–7.35 (m, 2H), 4.78 (s, 1H), 4.72 (s, 2H), 3.98 (s, 2H), 3.57 (s, 3H), 3.50 (q, *J* = 7.0 Hz, 2H), 1.14 (t, *J* = 7.0 Hz, 3H), 0.72 (s, 6H); ^{13}C NMR (101 MHz DMSO-d_6) δ 130.88, 129.89, 128.75, 127.60, 73.89, 65.20, 54.25, 28.24, 25.82, 17.71, 15.03, –2.19; MS (ESI$^+$), calcd $C_{18}H_{27}N_2O_3$ (M+H) = 319.2022, found = 319.2020.

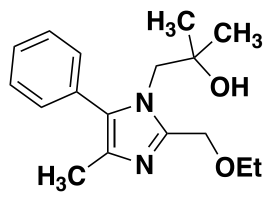

1-[2-(ethoxymethyl)-4-methyl-5-phenyl-1H-imidazol-1-yl]-2-methylpropan-2-ol (38, also called as 1h)

To a solution of 1-{2-[(tert-butyldimethylsilyl)oxy]-2-methylpropyl}-2-(ethoxymethyl)-4-methyl-5-phenyl-1H-imidazole (0.025 g, 0.062 mmol), in acetonitrile (1.3 mL, in an HDPE scintillation vial) was added hydrofluoric acid (1.3 mL, 48 wt % in water). The solution was allowed to stir at room temperature for 16 hours. After this time, saturated aqueous sodium carbonate was added until pH ≈ 10, and the resulting mixture was extracted with ethyl acetate (3 × 20 mL). The combined organic layers were was with saturated aqueous sodium chloride (40 mL), dried over magnesium sulfate, filtered and concentrated to dryness. The residue obtained was purified via flash SiO_2 chromatography (10 g silica gel, gradient of dichloromethane to 7% methanol/93% dichloromethane) to give 1-[2-(ethoxymethyl)-4-methyl-5-phenyl-1H-imidazol-1-yl]-2-methylpropan-2-ol **(38)** (0.016, 89%) as a white solid; ^{1}H NMR (400 MHz, DMSO-d_6) δ 7.47–7.44 (m, 2H), 7.37–7.33 (m, 1H), 7.30–7.28 (m, 2H), 4.62–4.62 (m, 3H), 3.98 (s, 2H), 3.49 (q, J = 7.0 Hz, 2H), 2.03 (s, 3H), 1.13 (t, J = 7.0 Hz, 3H), 0.70 (s, 6H); ^{13}C NMR (101 MHz DMSO-d_6) δ 145.20, 132.93, 131.36, 130.05, 129.13, 128.63, 127.30, 69.83, 64.97, 64.81, 53.38, 27.76, 15.08, 13.13; MS (ESI$^+$), calcd $C_{17}H_{25}N_2O_2$ (M+H) = 289.1916, found = 289.1905.

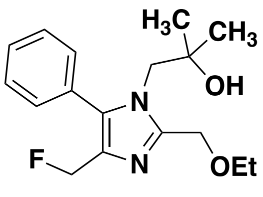

1-[2-(ethoxymethyl)-4-(fluoromethyl)-5-phenyl-1H-imidazol-1-yl]-2-methylpropan-2-ol (39, also called as 1i)

To a solution of 1-{2-[(tert-butyldimethylsilyl)oxy]-2-methylpropyl}-2-(ethoxymethyl)-4-(fluoromethyl)-5-phenyl-1H-imidazole (0.040 g, 0.095 mmol) in acetonitrile (2 mL, in an HDPE scintillation vial) was added hydrofluoric acid (2 mL, 48 wt % in water), and the solution was allowed to stir at room temperature for 17 hours. After this time, saturated aqueous sodium carbonate was added until pH ~ 10, and the resulting mixture was extracted with ethyl acetate (3 × 30 mL). The combined organic layers were was with saturated aqueous sodium chloride (50 mL), dried over magnesium sulfate, filtered and concentrated to dryness. The residue obtained was purified via flash SiO_2 chromatography (10 g silica gel, gradient of dichloromethane to 6% methanol/94% dichloromethane) to give 1-[2-(ethoxymethyl)-4-(fluoromethyl)-5-phenyl-1H-imidazol-1-yl]-2-methylpropan-2-ol **(39)** (0.019 g, 66%) as a colorless oil; ^{1}H NMR (400 MHz, DMSO-d_6) δ 7.53–7.49 (m, 2H), 7.46–7.42 (m, 1H), 7.39–7.37 (m, 2H), 5.07 (d, J = 50.4 Hz, 2H), 4.73 (s, 1H), 4.69 (s, 2H), 4.06 (s, 2H), 3.51 (q, J = 7.0 Hz, 2H), 1.14 (t, J = 7.0 Hz, 3H), 0.72 (s, 6H); ^{13}C NMR (101 MHz DMSO-d_6) δ 146.59, 134.55, 134.48, 132.52, 132.33, 130.06, 130.04, 129.67, 129.64, 128.82, 128.33, 78.49, 76.91, 69.79, 65.18, 64.84, 53.50, 27.73, 15.07; MS (ESI$^+$), calcd $C_{17}H_{24}FN_2O_2$ (M+H) = 307.1822, found = 307.1819.

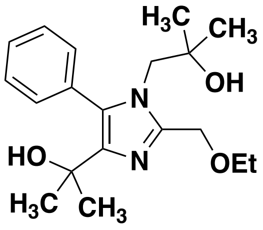

1-[2-(ethoxymethyl)-4-(2-hydroxypropan-2-yl)-5-phenyl-1H-imidazol-1-yl]-2-methylpropan-2-ol (40, also called as 1j)

To a solution of 2-(1-{2-[(tert-butyldimethylsilyl)oxy]-2-methylpropyl}-2-(ethoxymethyl)-5-phenyl-1H-imidazol-4-yl)propan-2-ol (0.030 g, 0.067 mmol) in tetrahydrofuran (2 mL) was added tetrabutylammonium fluoride (0.20 mL, 0.20 mmol, 1 M solution in tetrahydrofuran), and the solution was allowed to stir at room temperature for 22 hours. After this time, saturated aqueous ammonium chloride (10 mL) was added and the resulting mixture was extracted with ethyl acetate (3 × 5 mL). The combined organic layers were washed with saturated aqueous sodium chloride (10 mL), dried over magnesium sulfate, filtered and concentrated to dryness. The residue obtained was purified via flash SiO_2 chromatography (10 g silica gel, gradient of dichloromethane to 5% methanol/95% dichloromethane) to give 1-[2-(ethoxymethyl)-4-(2-hydroxypropan-2-yl)-5-phenyl-1H-imidazol-1-yl]-2-methylpropan-2-ol **(40)** (0.019 g, 86%) as a colorless oil; ^{1}H NMR (400 MHz, DMSO-d_6) δ 7.47–7.30 (m, 5H), 4.69 (s, 1H), 4.66 (s, 2H), 4.50 (s, 2H), 3.83 (s, 2H), 3.51 (q, J = 7.0 Hz, 2H), 1.25 (s, 6H), 1.14 (t, J = 7.0 Hz, 3H), 0.74 (s, 6H); ^{13}C NMR (101 MHz DMSO-d_6) δ 143.40, 143.27, 132.18, 131.87, 127.86, 127.82, 127.68, 69.59, 68.90, 65.17, 64.86, 53.05, 31.16, 27.94, 15.09; MS (ESI$^+$), calcd $C_{19}H_{29}N_2O_3$ (M+H) = 333.2178, found = 333.2191.

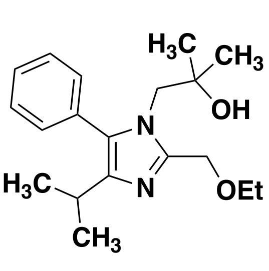

1-[2-(ethoxymethyl)-5-phenyl-4-(propan-2-yl)-1H-imidazol-1-yl]-2-methylpropan-2-ol (41, also called as 1k)

To a solution of 1-{2-[(tert-butyldimethylsilyl)oxy]-2-methylpropyl}-2-(ethoxymethyl)-5-phenyl-4-(propan-2-yl)-1H-imidazole (0.075 g, 0.17 mmol) in acetonitrile (3.5 mL, in an HDPE scintillation vial) was added hydrofluoric acid (3.5 mL, 48 wt % in water), and the solution was allowed to stir at room temperature for 17 hours. After this time, saturated aqueous sodium carbonate was added until pH ~ 10, and the resulting mixture was extracted with ethyl acetate (3 × 30 mL). The combined organic layers were was with saturated aqueous sodium chloride (30 mL), dried over magnesium sulfate, filtered and concentrated to dryness. The residue obtained was purified via flash SiO_2 chromatography (10 g silica gel, gradient of dichloromethane to 7% methanol/93% dichloromethane) to give 1-[2-(ethoxymethyl)-5-phenyl-4-(propan-2-yl)-1H-imidazol-1-yl]-2-methylpropan-2-ol **(41)** (0.052 g, 96%) as a white solid; ^{1}H NMR (400 MHz, DMSO-d_6) δ 7.47–7.44 (m, 2H), 7.38–7.34 (m, 1H), 7.28–7.25 (m, 2H), 4.64 (s, 3H), 3.95 (s, 2H), 3.51 (q, J = 7.0 Hz, 2H), 2.72 (sep, J = 6.8 Hz, 1H), 1.14 (t, J = 7.0 Hz, 3H), 1.10 (d, J = 6.8 Hz, 6H), 0.70 (s, 6H); ^{13}C NMR (101 MHz DMSO-d_6) δ 145.08, 142.72, 131.29, 130.39, 128.59, 127.64, 127.45, 69.82, 65.11, 65.04, 53.19, 27.81, 25.54, 23.22, 15.10; MS (ESI$^+$), calcd $C_{19}H_{29}N_2O_2$ (M+H) = 317.2229, found = 317.2223.

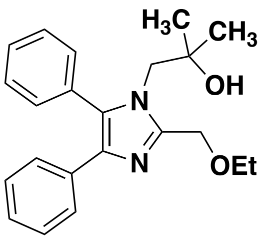

1-[2-(ethoxymethyl)-4,5-diphenyl-1H-imidazol-1-yl]-2-methylpropan-2-ol (42, also called as 1l)

To a solution of 1-{2-[(tert-butyldimethylsilyl)oxy]-2-methylpropyl}-2-(ethoxymethyl)-4,5-diphenyl-1H-imidazole (0.035 g, 0.075 mmol) in tetrahydrofuran (2 mL) was added tetrabutylammonium fluoride (0.23 mL, 0.23 mmol, 1 M solution in tetrahydrofuran). The solution was allowed to stir at room temperature for 17 hours. After this time, saturated aqueous ammonium chloride (5 mL) was added and the resulting mixture was extracted with ethyl acetate (3 × 5 mL). The combined organic layers were washed with saturated aqueous sodium chloride (10 mL), dried over magnesium sulfate, filtered and concentrated to dryness. The residue obtained was purified via flash SiO_2 chromatography (10 g silica gel, gradient of hexanes to ethyl acetate) to give 1-[2-(ethoxymethyl)-4,5-diphenyl-1H-imidazol-1-yl]-2-methylpropan-2-ol **(42)** (0.022 g, 85%) as an off-white solid; ^{1}H NMR (400 MHz, DMSO-d_6) δ 7.50–7.42 (m, 3H), 7.34–7.29 (m, 4H), 7.19–7.15 (m, 2H), 7.12–7.08 (m, 1H), 4.76 (s, 1H), 4.75 (s, 2H), 3.07 (s, 2H), 3.53 (q, J = 7.0 Hz, 2H), 1.15 (t, J = 7.0 Hz, 3H), 0.76 (s, 3H); MS (ESI$^+$), calcd $C_{22}H_{27}N_2O_2$ (M+H) = 351.2072, found = 315.2076.

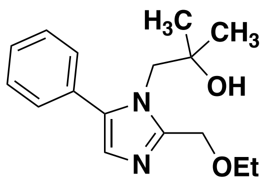

1-[2-(ethoxymethyl)-5-phenyl-1H-imidazol-1-yl]-2-methylpropan-2-ol (43, also called as 1m)

To a mixture of 1-{2-[(tert-butyldimethylsilyl)oxy]-2-methylpropyl}-2-(ethoxymethyl)-5-phenyl-1H-imidazole (0.050 g, 0.13 mmol) in tetrahydrofuran (3 mL) and tetrabutylammonium fluoride (0.39 mL, 0.39 mmol, 1 M solution in tetrahydrofuran) was added. The solution was allowed to stir at room temperature for 18 hours. After this time, saturated aqueous ammonium chloride (10 mL) was added and the resulting mixture was extracted with ethyl acetate (3 × 5 mL). The combined organic layers were washed with saturated aqueous sodium chloride, dried over magnesium sulfate, filtered and concentrated to dryness. The residue obtained was purified via flash SiO_2 chromatography (10 g silica gel, gradient of dichloromethane to 10% methanol/90% dichloromethane) to give 1-[2-(ethoxymethyl)-5-phenyl-1H-imidazol-1-yl]-2-methylpropan-2-ol **(43)** (0.032 g, 89%) as a yellow oil; ^{1}H NMR (400 MHz, DMSO-*d*$_6$) δ 7.46–7.33 (m, 5H), 6.88 (s, 1H), 4.67 (s, 2H), 4.65 (s, 1H), 4.12 (s, 2H), 3.50 (q, *J* = 7.0 Hz, 2 H), 1.14 (t, *J* = 7.0 Hz, 3H), 0.73 (s, 6H); ^{13}C NMR (101 MHz DMSO-*d*$_6$) δ 147.31, 134.13, 131.29, 128.75, 128.68, 127.47, 126.49, 69.92, 64.96, 53.31, 27.72, 15.04; MS (ESI$^+$), calcd $C_{16}H_{22}N_2O_2$ (M+H) = 275.2, found = 275.2.

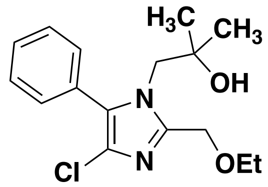

1-[4-chloro-2-(ethoxymethyl)-5-phenyl-1H-imidazol-1-yl]-2-methylpropan-2-ol (44, also called as 1n)

A solution of 1-{2-[(tert-butyldimethylsilyl)oxy]-2-methylpropyl}-2-(ethoxymethyl)-5-phenyl-1H-imidazole (0.050 g, 0.13 mmol) in *N*,*N*-dimethylformamide (2 mL) was

heated at 60 °C. A solution of *N*-chlorosuccinimide (0.027 g, 0.20 mmol) in *N*,*N*-dimethylformamide (1 mL) was added, and the solution was allowed to stir at 60 °C for 15 hours. After this time, the solution was allowed to cool to room temperature and water (20 mL) was added. Solid sodium thiosulfate pentahydrate was added until no further color change was observed, and the resulting mixture was extracted with ethyl acetate (3 × 10 mL). The combined organic layers were washed with 5% aqueous lithium chloride (3 × 15 mL), washed with saturated aqueous sodium chloride (30 mL), dried over magnesium sulfate, filtered and concentrated to dryness. The residue obtained was filtered through a plug of silica gel (~ 10 g, eluting with ethyl acetate) and the filtrate was concentrated to dryness to give crude 1-{2-[(tert-butyldimethylsilyl)oxy]-2-methylpropyl}-4-chloro-2-(ethoxymethyl)-5-phenyl-1H-imidazole **(28)** (0.055 g, >99%). The residue was dissolved in tetrahydrofuran (5 mL) and tetrabutylammonium fluoride (0.39 mL, 0.39 mmol, 1 M solution in tetrahydrofuran) was added. The solution was allowed to stir at room temperature for 16 hours. After this time, saturated aqueous ammonium chloride (10 mL) was added and the resulting mixture was extracted with ethyl acetate (3 × 5 mL). The combined organic layers were washed with saturated aqueous sodium chloride, dried over magnesium sulfate, filtered and concentrated to dryness. The residue obtained was purified via flash SiO_2 chromatography (10 g silica gel, gradient of hexanes to 60% ethyl acetate/40% hexanes) to give 1-[4-chloro-2-(ethoxymethyl)-5-phenyl-1H-imidazol-1-yl]-2-methylpropan-2-ol **(44)** (0.037 g, 93%) as a white solid; ^{1}H NMR (400 MHz, DMSO-d_6) δ 7.51–7.47 (m, 2H), 7.44–7.39 (m, 3H), 4.73 (s, 1H), 4.64 (s, 2H), 4.04 (s, 2H), 3.51 (q, *J* = 7.0 Hz, 2H), 1.14 (t, *J* = 7.0 Hz, 3H), 0.73 (s, 6H); ^{13}C NMR (101 MHz DMSO-d_6) δ 145.27, 130.11, 128.78, 128.68, 128.27,

127.91, 125.17, 69.63, 65.13, 64.45, 54.00, 27.60, 14.98; MS (ESI$^+$), calcd $C_{16}H_{22}ClN_2O_2$ (M+H) = 309.1370, found = 309.1376.

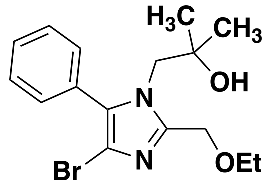

1-[4-bromo-2-(ethoxymethyl)-5-phenyl-1H-imidazol-1-yl]-2-methylpropan-2-ol (45, also called as 1o)

To a solution of 4-bromo-1-{2-[(tert-butyldimethylsilyl)oxy]-2-methylpropyl}-2-(ethoxymethyl)-5-phenyl-1H-imidazole (0.050 g, 0.11 mmol) in tetrahydrofuran (5 mL) was added tetrabutylammonium fluoride (0.33 mL, 0.33 mmol, 1 M solution in tetrahydrofuran) and the solution was allowed to stir for 17 hours. After this time, saturated aqueous ammonium chloride (10 mL) was added and the resulting mixture was extracted with ethyl acetate (3 × 5 mL). The combined organic layers were dried over magnesium sulfate, filtered and concentrated to dryness. The residue obtained was purified via flash SiO_2 chromatography (10 g silica gel, gradient of dichloromethane to 8% methanol/ 92% dichloromethane) to give 1-[4-bromo-2-(ethoxymethyl)-5-phenyl-1H-imidazol-1-yl]-2-methylpropan-2-ol **(45)** (0.034 g, 87%) as a white solid; ^{1}H NMR (400 MHz, DMSO-d_6) δ 7.51–7.47 (m, 2H), 7.44–7.38 (m, 3H), 7.43 (s, 1H), 4.64 (s, 2H), 4.04 (s, 2H), 3.50 (q, J = 7.0 Hz, 2H), 1.14 (t, J = 7.0 Hz, 3H), 0.73 (s, 6H); ^{13}C NMR (101 MHz DMSO-d_6) δ 146.63, 130.67, 130.38, 129.25, 128.67, 128.37, 113.24, 69.67, 65.19, 64.52, 54.10, 27.65, 15.03; MS (ESI$^+$), calcd $C_{16}H_{22}BrN_2O_2$ (M+H) = 353.0865 & 355.0846, found = 353.0868 & 355.0847.

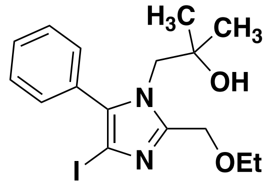

1-[2-(ethoxymethyl)-4-iodo-5-phenyl-1H-imidazol-1-yl]-2-methylpropan-2-ol (CU-CPD107)

To a solution of 1-{2-[(tert-butyldimethylsilyl)oxy]-2-methylpropyl}-2-(ethoxymethyl)-4-iodo-5-phenyl-1H-imidazole (0.040 g, 0.078 mmol) in tetrahydrofuran (5 mL) was added tetrabutylammonium fluoride (0.23 mL, 0.23 mmol, 1 M solution in tetrahydrofuran) and the solution was allowed to stir at room temperature for 17 hours. After this time, saturated aqueous ammonium chloride (10 mL) was added and the mixture was extracted with ethyl acetate (3 × 5 mL). The combined organic layers were washed with saturated aqueous sodium chloride (10 mL), dried over magnesium sulfate, filtered and concentrated to dryness. The residue obtained was purified via flash SiO_2 chromatography (10 g silica gel, gradient of hexanes to 70% ethyl acetate/30% hexanes) to give 1-[2-(ethoxymethyl)-4-iodo-5-phenyl-1H-imidazol-1-yl]-2-methylpropan-2-ol **(CU-CPD107)** (0.027 g, 87%) as a white solid; ^{1}H NMR (400 MHz, DMSO-d_6) δ 7.51–7.47 (m, 2H), 7.44–7.38 (m, 3H), 4.74 (s, 2H), 4.65 (s, 2H), 4.04 (s, 2H), 3.50 (q, J = 7.0 Hz, 2H), 1.14 (t, J = 7.0 Hz, 3H), 0.73 (s, 6H); ^{13}C NMR (101 MHz DMSO-d_6) δ 146.62, 130.65, 130.37, 129.25, 128.66, 128.35, 113.24, 69.66, 65.17, 64.52, 54.09, 27.64, 15.03; MS (ESI$^+$), calcd $C_{16}H_{22}IN_2O_2$ (M+H) = 401.0726, found = 401.0731.

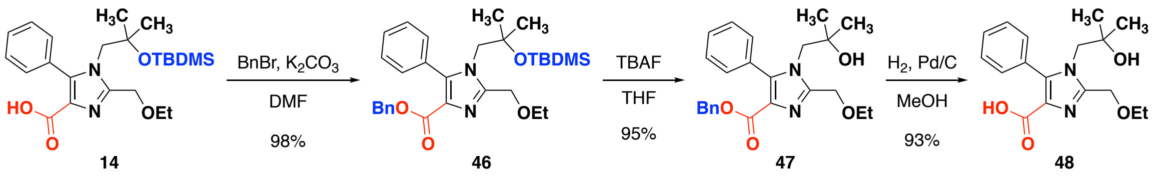

Supplementary Fig. 18 Carboxylic acid analog deprotection: Carboxylic acid **14** was deprotected over three steps to give **48**.

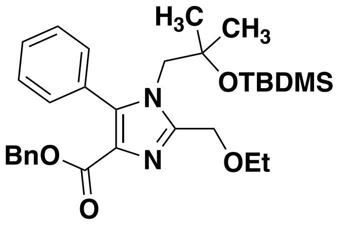

benzyl 1-{2-[(tert-butyldimethylsilyl)oxy]-2-methylpropyl}-2-(ethoxymethyl)-5-phenyl-1H-imidazole-4-carboxylate (46)

To a mixture of 1-{2-[(tert-butyldimethylsilyl)oxy]-2-methylpropyl}-2-(ethoxymethyl)-5-phenyl-1H-imidazole-4-carboxylic acid (0.100 g, 0.231 mmol), potassium carbonate (0.128 g, 0.924 mmol) in *N*,*N*-dimethylformamide (3 mL) was added benzyl bromide (42 μL, 0.35 mmol), and the resulting mixture was allowed to stir at room temperature for 17 hours. After this time, the mixture was diluted with water (20 mL) and extracted with ethyl acetate (4 × 10 mL). The combined organic layers were washed with 5% aqueous lithium chloride (3 × 30 mL), washed with saturated aqueous sodium chloride (40 mL), dried over magnesium sulfate, filtered and concentrated to dryness. The residue obtained was purified via flash SiO_2 chromatography (10 g silica gel, gradient of hexanes to 35% ethyl acetate/65% hexanes)to give benzyl 1-{2-[(tert-butyldimethylsilyl)oxy]-2-methylpropyl}-2-(ethoxymethyl)-5-phenyl-1H-imidazole-4-carboxylate **(46)** (0.118 g,

98%) as a white solid; ^{1}H NMR (400 MHz, DMSO-d_6) δ 7.44–7.38 (m, 5H), 7.32–7.26 (m, 3H), 7.17–7.12 (m, 2H), 5.10 (s, 2H), 4.65 (s, 2H), 4.07 (s, 2H), 3.49 (q, J = 7.0 Hz, 2H), 1.14 (t, J = 7.0 Hz, 3H), 0.83 (s, 6H), 0.72 (s, 9H), –0.03 (s, 6H); ^{13}C NMR (101 MHz DMSO-d_6) δ 162.21, 146.69, 139.36, 136.20, 131.01, 129.54, 128.69, 128.25, 128.09, 127.86, 127.81, 127.78, 73.60, 65.32, 64.97, 64.91, 54.24, 28.32, 25.80, 17.71, 14.98, –2.20; MS (ESI$^+$), calcd $C_{30}H_{43}N_2O_4Si$ (M+H) = 523.2992, found = 523.3004.

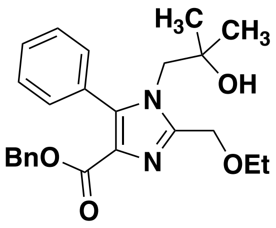

benzyl 2-(ethoxymethyl)-1-(2-hydroxy-2-methylpropyl)-5-phenyl-1H-imidazole-4-carboxylate (47)

To a solution of benzyl 1-{2-[(tert-butyldimethylsilyl)oxy]-2-methylpropyl}-2-(ethoxymethyl)-5-phenyl-1H-imidazole-4-carboxylate (0.108 g, 0.207 mmol) in tetrahydrofuran (3 mL) was added tetrabutylammonium fluoride (0.62 mL, 0.62 mmol, 1 M in tetrahydrofuran), and the solution was allowed to stir for 15 hours. After this time, saturated aqueous ammonium chloride (10 mL) was added and the resulting mixture was extracted with ethyl acetate (3 × 5 mL). The combined organic layers were washed with saturated aqueous sodium chloride (10 mL), dried over magnesium sulfate, filtered and concentrated to dryness. The residue obtained was purified via flash SiO_2 chromatography (10 g silica gel, gradient of hexanes to 95% ethyl acetate/5% hexanes) to give benzyl 2-(ethoxymethyl)-1-(2-hydroxy-2-methylpropyl)-5-phenyl-1H-imidazole-4-carboxylate **(47)** (0.081 g, 95%) as a white solid; ^{1}H NMR (400 MHz, DMSO-d_6) δ 7.43–

7.39 (m, 3H), 7.39–7.35 (m, 2H), 7.16–7.12 (m, 2H), 5.08 (s, 2H), 4.79 (s, 1H), 4.72 (s, 2H), 3.97 (s, 2H), 3.49 (q, J = 7.0 Hz, 2H), 1.13 (t, J = 7.0 Hz, 3H), 0.73 (s, 6H); ^{13}C NMR (101 MHz DMSO-d_6) δ 162.28, 146.95, 139.65, 136.26, 131.11, 129.63, 128.60, 128.27, 127.98, 127.79, 127.67, 69.59, 65.23, 64.90, 64.88, 53.40, 27.84, 15.06; MS (ESI$^+$), calcd $C_{24}H_{29}N_2O_4$ (M+H) = 409.2127, found = 409.2134.

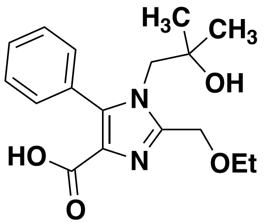

2-(ethoxymethyl)-1-(2-hydroxy-2-methylpropyl)-5-phenyl-1H-imidazole-4-carboxylic acid (48, also called as 1q)

To solution of benzyl 2-(ethoxymethyl)-1-(2-hydroxy-2-methylpropyl)-5-phenyl-1H-imidazole-4-carboxylate (0.076 g, 0.19 mmol) in methanol (5 mL) was added palladium on carbon (0.008 g, 5% basis). The mixture was degassed and backfilled with nitrogen gas (4×), then degassed and backfilled with hydrogen gas (4x). The reaction was then allowed to stir under hydrogen atmosphere (balloon) for 17 hours. After this time, the mixture was degassed and backfilled with nitrogen gas (4x), diluted with dichloromethane (15 mL), and filtered through Celite (rinsing with additional dichloromethane). The filtrate was concentrated to dryness. The resulting solid was triturated with cold diethyl ether to give 2-(ethoxymethyl)-1-(2-hydroxy-2-methylpropyl)-5-phenyl-1H-imidazole-4-carboxylic acid **(48)** (0.056 g, 93%) as a white solid; ^{1}H NMR (400 MHz, DMSO-d_6) δ 11.9 (br s, 1H), 7.46–7.35 (m, 5H), 4.77–4.71 (2 x s, 3H), 3.97 (s, 2H), 3.50 (q, J = 7.0 Hz, 2H), 1.14 (t, J = 7.0 Hz, 3H), 0.72 (s, 6H); ^{13}C NMR (101 MHz

DMSO-d_6) δ 163.88, 146.48, 138.83, 131.15, 129.97, 128.52, 128.39, 127.85, 69.61, 65.18, 64.90, 53.31, 27.82, 15.07; MS (ESI$^+$), calcd $C_{17}H_{23}N_2O_4$ (M–H) = 317.1501, found = 317.1517.

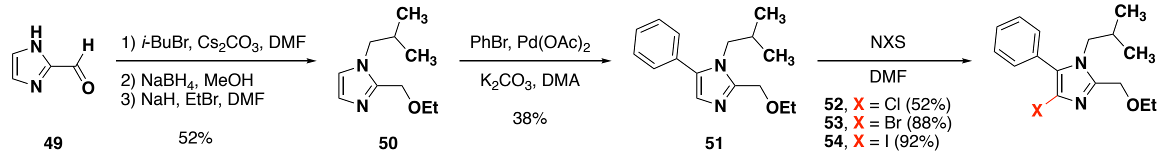

Supplementary Fig. 19 R848/R837/CU-CPD107 hybrid synthesis: Hybrid molecules **52**, **53**, and **54** were made from **49**.

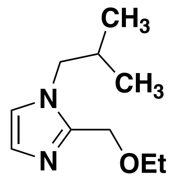

2-(ethoxymethyl)-1-(2-methylpropyl)-1H-imidazole (50)

To a mixture of 2-imidazolecarboxaldehyde **(49)** (5.00 g, 52.0 mmol) and cesium carbonate (33.9 g, 104 mmol) in *N*,*N*-dimethylformamide (250 mL) was added 1-bromo-2-methylpropane (6.2 mL, 57 mmol), and was allowed to stir at room temperature for 18 hours. After this time, the mixture was diluted with ethyl acetate (500 mL), washed with water (2 × 300 mL), washed with 5% aqueous lithium chloride (2 × 200 mL), washed with saturated aqueous sodium chloride (200 mL), dried over magnesium sulfate, filtered and concentrated to dryness to give crude 1-(2-methylpropyl)-1H-imidazole-2-carbaldehyde as a yellow oil. The oil obtained was dissolved in methanol (250 mL). Sodium borohydride was (2.95 g, 78.0 mmol) added and allowed to stir at room temperature for 17 hours. After this time, saturated aqueous ammonium chloride (200 mL)

was added and the resulting mixture was extracted with dichloromethane (3 x 200 mL). The combined organic layers were washed with saturated aqueous sodium chloride (400 mL), dried over sodium sulfate, filtered and concentrated to dryness. The resulting residue was azeotroped with additional dichloromethane and left on a high-vacuum pump for several hours to give crude [1-(2-methylpropyl)-1H-imidazol-2-yl]methanol as a clear, yellow oil. The yellow oil was dissolved in *N*,*N*-dimethylformamide (250 mL) and cooled in an ice bath (~ 0 °C). Sodium hydride (4.17 g, 104 mmol, 60% dispersion in mineral oil) was added and the mixture was allowed to stir in the ice bath for 45 min. After this time, bromoethane (9.7 mL, 130 mmol) was added and the mixture was allowed to stir in the ice bath for 1 hour. After this time, the ice bath was removed and the mixture was allowed to stir at room temperature for 15 hours. After this time, saturated aqueous ammonium chloride (200 mL) and water (50 mL) were added and the resulting mixture was extracted with ethyl acetate (500 mL). The organic layer was washed with water (2 × 400 mL), washed with 5% aqueous lithium chloride (400 mL), washed with saturated aqueous sodium chloride (400 mL), dried over magnesium sulfate, filtered and concentrated to dryness. The residue obtained was filtered through a plug of silica gel (≈ 70 g). The filtrate was concentrated to give 2-(ethoxymethyl)-1-(2-methylpropyl)-1H-imidazole **(50)** (4.966 g, 52% over three steps) as a colorless oil; ^{1}H NMR (400 MHz, DMSO-d_6) δ 7.15 (d, J = 1.2 Hz, 1H), 6.81 (d, J = 1.2 Hz, 1H), 4.44 (s, 2H), 3.75 (d, J = 7.5 Hz, 2H), 3.42 (q, J = 7.0 Hz, 2H), 2.05 (sep, J = 6.7 Hz, 1H), 1.09 (t, J = 7.0 Hz, 3H), 0.84 (d, J = 6.7 Hz, 6H); ^{13}C NMR (101 MHz DMSO-d_6) δ 144.17, 126.60, 121.37, 64.71, 63.62, 52.46, 29.24, 19.66, 14.96; MS (ESI^{+}), calcd $C_{10}H_{19}N_2O$ (M+H) = 183.1497, found = 183.1498.

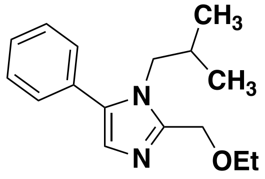

2-(ethoxymethyl)-1-(2-methylpropyl)-5-phenyl-1H-imidazole (51, also called as 2a)

A mixture of 2-(ethoxymethyl)-1-(2-methylpropyl)-1H-imidazole (1.00 g, 1.3 mmol), bromobenzene (0.44 mL, 4.2 mmol), and potassium acetate (1.16 g, 8.40 mmol) in *N*,*N*-dimethylacetamide (30 mL) was degassed and backfilled with nitrogen gas (4×). Palladium (II) acetate (0.094 g, 0.42 mmol) was added, the mixture was degassed and backfilled with nitrogen gas (4x) again and heated at 150 °C for 16 hours. After this time, the mixture was allowed to cool to room temperature, diluted with ethyl acetate (120 mL), washed with water (100 mL), washed with 5% aqueous lithium chloride (3 × 100 mL), washed with saturated aqueous sodium chloride (100 mL), dried over magnesium sulfate, filtered and concentrated to dryness. The residue obtained was purified via flash SiO_2 chromatography (100 g silica gel, gradient of hexanes to 50% ethyl acetate/50% hexanes) to give 2-(ethoxymethyl)-1-(2-methylpropyl)-5-phenyl-1H-imidazole **(51)** (0.540 g, 38%) as a yellow oil; ^{1}H NMR (400 MHz, DMSO-d_6) δ 7.48–7.43 (m, 4H), 7.41–7.36 (m, 1H), 6.92 (s, 1H), 4.52 (s, 2H), 3.94 (d, *J* = 7.7 Hz, 2H), 3.51 (q, *J* = 7.0 Hz, 2H), 1.64 (sep, *J* = 6.7 Hz, 1H), 1.13 (t, *J* = 7.0 Hz, 3H), 0.60 (d, *J* = 6.7 Hz, 6H); ^{13}C NMR (101 MHz DMSO-d_6) δ 145.76, 133.60, 130.77, 128.79, 128.23, 127.75, 126.62, 64.90, 64.23, 50.62, 28.48, 19.36, 14.96.

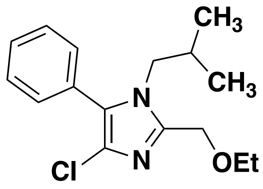

4-chloro-2-(ethoxymethyl)-1-(2-methylpropyl)-5-phenyl-1H-imidazole (52, also called as 2b)

To a solution of 2-(ethoxymethyl)-1-(2-methylpropyl)-5-phenyl-1H-imidazole (0.050 g, 0.19 mmol) in *N*,*N*-dimethylformamide (2 mL) at 60 °C was added a solution of *N*-chlorosuccinimide (0.039 g, 0.29 mmol) in *N*,*N*-dimethylformamide (1 mL) and was allowed to stir at 60 °C for 18 hours. After this time, the solution was allowed to cool to room temperature, diluted with water (10 mL), added solid sodium thiosulfate pentahydrate until the mixture was white, and extracted with ethyl acetate (10 mL). The organic layer was washed with 5% aqueous lithium chloride (2 × 10 mL), washed with saturated aqueous sodium chloride (10 mL), dried over magnesium sulfate, filtered and concentrated to dryness. The residue obtained was purified via flash SiO_2 chromatography (10 g silica gel, gradient of hexanes to 40% ethyl acetate/60% hexanes) to give 4-chloro-2-(ethoxymethyl)-1-(2-methylpropyl)-5-phenyl-1H-imidazole **(52)** (0.029 g, 52%) as a white solid; ^{1}H NMR (400 MHz, DMSO-d_6) δ 7.53–7.41 (m, 5H), 4.50 (s, 2H), 3.86 (d, *J* = 7.7 Hz, 2H), 3.51 (q, *J* = q, 7.0 Hz, 2H), 1.57 (sep, *J* = 6.7 Hz, 1H), 1.14 (t, *J* = 7.0 Hz, 3H), 0.59 (d, *J* = 6.7 Hz, 6H); ^{13}C NMR (101 MHz DMSO-d_6) δ 143.54, 129.73, 128.84, 128.67, 128.37, 127.64, 125.19, 65.08, 63.62, 51.31, 28.20, 19.28, 14.92.

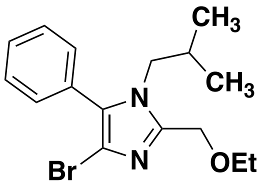

4-bromo-2-(ethoxymethyl)-1-(2-methylpropyl)-5-phenyl-1H-imidazole (53, also called as 2c)

To a solution of 2-(ethoxymethyl)-1-(2-methylpropyl)-5-phenyl-1H-imidazole (0.107 g, 0.414 mmol) in *N*,*N*-dimethylformamide (4 mL) was added a solution of *N*-bromosuccinimide (0.077 g, 0.43 mmol) in *N*,*N*-dimethylformamide (2 mL), and was allowed to stir at room temperature for 48 hours. After this time, the solution was diluted with water (15 mL), added solid sodium thiosulfate pentahydrate until the mixture was white, and extracted with ethyl acetate (20 mL). The organic layer was washed with 5% aqueous lithium chloride (2 × 20 mL), washed with saturated aqueous sodium chloride (20 mL), dried over magnesium sulfate, filtered and concentrated to dryness. The residue obtained was purified via flash SiO_2 chromatography (25 g silica gel, gradient of hexanes to 40% ethyl acetate/60% hexanes) to give 4-bromo-2-(ethoxymethyl)-1-(2-methylpropyl)-5-phenyl-1H-imidazole **(53)** (0.123 g, 88%) as a white solid; ^{1}H NMR (400 MHz, DMSO-d_6) δ 7.54–7.40 (m, 5H), 4.50 (s, 2H), 3.85 (d, J = 7.7 Hz, 2H), 3.51 (q, J = 7.0 Hz, 2H), 1.57 (sep, J = 6.7 Hz, 1H), 1.14 (t, J = 7.0 Hz, 3H), 0.59 (d, J = 6.7 Hz, 6H).

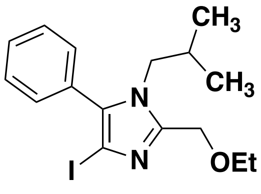

2-(ethoxymethyl)-4-iodo-1-(2-methylpropyl)-5-phenyl-1H-imidazole (54, also called as 2d)

To a solution of 2-(ethoxymethyl)-1-(2-methylpropyl)-5-phenyl-1H-imidazole (0.050 g, 0.19 mmol) in *N*,*N*,-dimethylformamide (2 mL) at 60 °C was added a solution of *N*-iodosuccinimide (0.065 g, 0.29 mmol) in *N*,*N*-dimethylformamide (1 mL), and was allowed to stir at 60 °C for 18 hours. After this time, the solution was allowed to cool to room temperature, diluted with water (10 mL), added solid sodium thiosulfate pentahydrate until the mixture was white, and extracted with ethyl acetate (20 mL). The organic layer was washed with 5% aqueous lithium chloride (2 × 20 mL), washed with saturated aqueous sodium chloride (20 mL), dried over magnesium sulfate, filtered and concentrated to dryness. The residue obtained was purified via flash SiO_2 chromatography (10 g silica gel, gradient of hexanes to 40% ethyl acetate/60% hexanes) to give 2-(ethoxymethyl)-4-iodo-1-(2-methylpropyl)-5-phenyl-1H-imidazole **(54)** (0.067g, 92%) as a white solid; ^{1}H NMR (400 MHz, DMSO-d_6) δ 7.52–7.43 (m, 3H), 7.39–7.36 (m, 2H), 4.49 (s, 2H), 3.83 (d, *J* = 7.7 Hz, 2H), 3.51 (q, *J* = 7.0 Hz, 2H), 1.55 (sep, *J* = 6.7 Hz, 1H), 1.13 (t, *J* = 7.0 Hz, 3H), 0.57 (d, *J* = 6.7 Hz, 6H); ^{13}C NMR (101 MHz DMSO-d_6) δ 147.18, 135.52, 130.22, 129.89, 128.72, 128.68, 84.64, 65.07, 63.70, 51.40, 28.25, 19.31, 14.94.

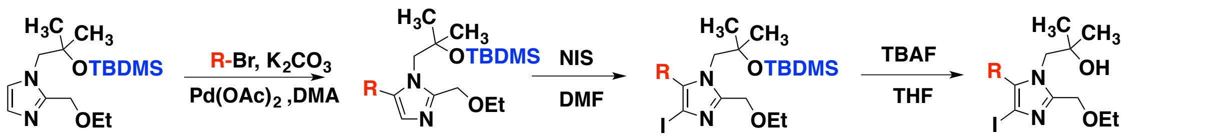

5

55, R = 4-biphenyl (13%)
56, R = 3-biphenyl (10%)
57, R = *o*-methyl phenyl (31%)
58, R = *m*-methyl phenyl (23%)
59, R = *p*-methyl phenyl (5%)

60, R = 4-biphenyl (45%)
61, R = 3-biphenyl (21%)
62, R = *o*-methyl phenyl (82%)
63, R = *m*-methyl phenyl (57%)
64, R = *p*-methyl phenyl (61%)

3a, R = 4-biphenyl (99%)
3b, R = 3-biphenyl (95%)
3c, R = *o*-methyl phenyl (84%)
3d, R = *m*-methyl phenyl (71%)
3e, R = *p*-methyl phenyl (72%)

Supplementary Fig. 20 Aromatic analogs synthesis: 3a, **3b**, **3c**, **3d**, and **3e** were made from imidazole intermediate **5**.

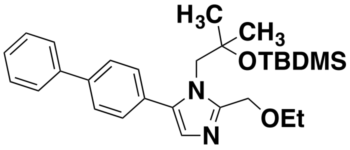

5-([1,1'-biphenyl]-4-yl)-1-(2-((tert-butyldimethylsilyl)oxy)-2-methylpropyl)-2-(ethoxymethyl)-1H-imidazole (55)

A mixture of 1-{2-[(tert-butyldimethylsilyl)oxy]-2-methylpropyl}-2-(ethoxymethyl) 1H-imidazole (312 mg, 1.00 mmol), 4-bromo-1,1'-biphenyl (466 mg， 2.00 mmol), and potassium carbonate (552mg， 4.00mmol) in N,N-dimethylacetamide (10 mL) was degassed and backfilled with nitrogen gas (4 ×). Palladium (II) acetate (38mg， 0.17mmol) was added and the mixture was degassed and backfilled with nitrogen gas (4 ×) again. The mixture was heated at 80 °C for 12 hours, and after this time the reaction was allowed to cool to room temperature. Water (20 mL) was added and the resulting mixture was extracted with ethyl acetate (3 x 100 mL). The combined organic layers were washed with 5% aqueous lithium chloride (3 x 100 mL), washed with saturated aqueous sodium chloride (300 mL), dried over magnesium sulfate, filtered and concentrated to dryness. The residue obtained was purified via flash SiO_2 chromatography (34 g silica gel, gradient of hexanes to 25% ethyl acetate/75% hexanes) to give 5-([1,1'-biphenyl]-4-yl)-1-(2-((*tert*-butyldimethylsilyl)oxy)-2-methylpropyl)-2-(ethoxymethyl)-1*H*-imidazole**(55)**(60 mg, 13%) as a brown oil. ^{1}H NMR (400 MHz, $CDCl_3$) δ 7.66-7.62 (m, 4H), 7.49-7.40 (m, 5H), 7.03 (s, 1H), 4.25 (s, 2H), 3.58 (q, *J* = 7.0 Hz, 2H), 1.25 (d, *J* =

7.0 Hz, 3H), 0.95 (s, 6H), 0.78 (s, 9H), -0.02 (s, 6H); ^{13}C NMR (101 MHz, $CDCl_3$) δ 147.87, 140.43, 134.25, 130.76, 129.26, 128.98, 127.74, 127.66, 127.53, 127.09, 77.16, 74.54, 66.05, 65.89, 55.16, 28.72, 26.07, 18.14, 15.27, -1.92.

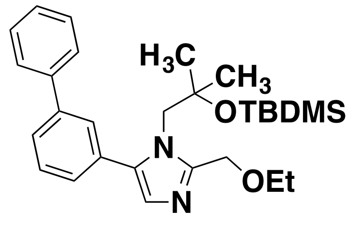

5-([1,1'-biphenyl]-3-yl)-1-(2-((*tert*-butyldimethylsilyl)oxy)-2-methylpropyl)-2-(ethoxymethyl)-1*H*-imidazole (56)

A mixture of 1-{2-[(tert-butyldimethylsilyl)oxy]-2-methylpropyl}-2-(ethoxymethyl) 1*H*-imidazole (312 mg, 1.00 mmol), 3-bromo-1,1'-biphenyl (466 mg，2.00 mmol), and potassium carbonate (552mg， 4.00mmol) in N,N-dimethylacetamide (10 mL) was degassed and backfilled with nitrogen gas (4 ×). Palladium (II) acetate (38mg， 0.17mmol) was added and the mixture was degassed and backfilled with nitrogen gas (4×) again. The mixture was heated at 80 °C for 12 hours, and after this time the reaction was allowed to cool to room temperature. Water (20 mL) was added and the resulting mixture was extracted with ethyl acetate (3 × 100 mL). The combined organic layers were washed with 5% aqueous lithium chloride (3 × 100 mL), washed with saturated aqueous sodium chloride (300 mL), dried over magnesium sulfate, filtered and concentrated to dryness. The residue obtained was purified via flash SiO_2 chromatography (34 g silica gel, gradient of hexanes to 25% ethyl acetate/75% hexanes) to give 5-([1,1'-biphenyl]-3-yl)-1-(2-((*tert*-butyldimethylsilyl)oxy)-2-methylpropyl)-2-(ethoxymethyl)-1*H*-

imidazole**(56)**(48 mg, 10%) as a yellow oil. ^1H NMR (400 MHz, $CDCl_3$) δ 7.61-7.54 (m, 4H), 7.50-7.43 (m, 3H), 7.39-7.31 (m, 2H), 7.04 (s, 1H), 4.78 (s, 2H), 4.24 (s, 2H), 3.58 (q, J = 7.0 Hz, 2H), 1.24 (t, J = 7.0 Hz, 3H), 0.94 (s, 6H), 0.78 (s, 9H); ^{13}C NMR (101 MHz, $CDCl_3$) δ 147.71, 141.85, 140.60, 134.38, 132.21, 129.22, 128.87, 127.68, 127.65, 127.56, 127.15, 126.43, 74.42, 65.93, 65.81, 55.08, 28.62, 25.96, 18.02, 15.15, -2.04.

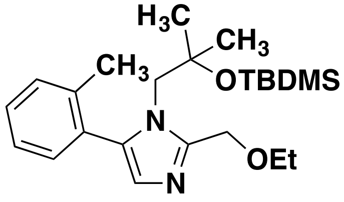

1-(2-((tert-butyldimethylsilyl)oxy)-2-methylpropyl)-2-(ethoxymethyl)-5-(*o*-tolyl)-1*H*-imidazole (57)

A mixture of 1-{2-[(tert-butyldimethylsilyl)oxy]-2-methylpropyl}-2-(ethoxymethyl) 1H-imidazole (469 mg, 1.50 mmol), 1-bromo-2-methylbenzene (513 mg, 3.00 mmol), and potassium carbonate (690mg, 5.00mmol) in N,N-dimethylacetamide (10 mL) was degassed and backfilled with nitrogen gas (4×). Palladium (II) acetate (76 mg，0.34 mmol) was added and the mixture was degassed and backfilled with nitrogen gas (4×) again. The mixture was heated at 80 °C for 12 hours, and after this time the reaction was allowed to cool to room temperature. Water (20 mL) was added and the resulting mixture was extracted with ethyl acetate (3 × 100 mL). The combined organic layers were washed with 5% aqueous lithium chloride (3 × 100 mL), washed with saturated aqueous sodium chloride (300 mL), dried over magnesium sulfate, filtered and concentrated to dryness. The residue obtained was purified via flash SiO_2 chromatography (34 g silica gel, gradient of hexanes to 25% ethyl acetate/75% hexanes)

to give 1-(2-((*tert*-butyldimethylsilyl)oxy)-2-methylpropyl)-2-(ethoxymethyl)-5-(o-tolyl)-1H-imidazole(**57**)(186 mg, 31%) as a brown oil. ^{1}H NMR (400 MHz, $CDCl_3$) δ 7.29 – 7.17 (m, 4H), 6.94 (s, 1H), 4.79 (s, 2H), 4.01 (s, 2H), 3.54 (q, J = 7.0 Hz, 2H), 2.23 (s, 3H), 1.22 (t, J = 7.0 Hz, 3H), 0.93 (s, 6H), 0.79 (s, 9H). ^{13}C NMR (101 MHz, $CDCl_3$) δ 147.08, 137.58, 132.83, 131.24, 130.77, 130.53, 128.43, 127.79, 125.86, 74.35, 65.96, 65.66, 55.02, 28.48, 26.08, 20.18, 18.17, 15.28, -1.89.

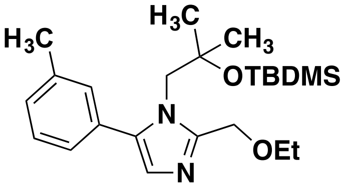

1-(2-((*tert*-butyldimethylsilyl)oxy)-2-methylpropyl)-2-(ethoxymethyl)-5-(*m*-tolyl)-1*H*-imidazole (58)

A mixture of 1-{2-[(tert-butyldimethylsilyl)oxy]-2-methylpropyl}-2-(ethoxymethyl) 1H-imidazole (469 mg, 1.50 mmol), 1-bromo-3-methylbenzene (514 mg， 3.00 mmol), and potassium carbonate (552mg， 4.00mmol) in N,N-dimethylacetamide (10 mL) was degassed and backfilled with nitrogen gas (4×). Palladium (II) acetate (38mg，0.17mmol) was added and the mixture was degassed and backfilled with nitrogen gas (4×) again. The mixture was heated at 80 °C for 12 hours, and after this time the reaction was allowed to cool to room temperature. Water (20 mL) was added and the resulting mixture was extracted with ethyl acetate (3 × 100 mL). The combined organic layers were washed with 5% aqueous lithium chloride (3 × 100 mL), washed with saturated aqueous sodium chloride (300 mL), dried over magnesium sulfate, filtered and concentrated to dryness. The residue obtained was purified via flash SiO_2

chromatography (34 g silica gel, gradient of hexanes to 25% ethyl acetate/75% hexanes) to give 1-(2-((*tert*-butyldimethylsilyl)oxy)-2-methylpropyl)-2-(ethoxymethyl)-5-(*m*-tolyl)-1*H*-imidazole**(58)**(138 mg, 23%) as an orange oil. ^{1}H NMR (400 MHz, Chloroform-*d*) δ 7.18 – 7.10 (m, 4H), 6.96 (s, 1H), 4.76 (s, 2H), 4.20 (s, 2H), 3.55 (q, *J* = 7.0 Hz, 2H), 2.38 (s, 3H), 1.22 (t, *J* = 7.0 Hz, 4H), 0.90 (d, *J* = 7.6 Hz, 6H), 0.78 (s, 9H), -0.03(s, 6H). ^{13}C NMR (101 MHz, $CDCl_3$) δ 147.41, 138.46, 134.60, 131.53, 129.50, 128.64, 128.39, 127.24, 125.88, 76.71, 74.41, 65.85,65.74, 54.93, 28.55, 25.94, 21.44, 18.02, 15.13, -2.06.

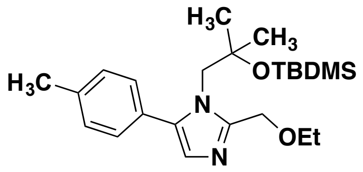

1-(2-((*tert*-butyldimethylsilyl)oxy)-2-methylpropyl)-2-(ethoxymethyl)-5-(*p*-tolyl)-1*H*-imidazole (59)

A mixture of 1-{2-[(tert-butyldimethylsilyl)oxy]-2-methylpropyl}-2-(ethoxymethyl) 1H-imidazole (312 mg, 1.00 mmol), 1-bromo-4-methylbenzene (513 mg，3.00 mmol), and potassium carbonate (552mg， 4.00mmol) in N,N-dimethylacetamide (10 mL) was degassed and backfilled with nitrogen gas (4 ×). Palladium (II) acetate (38mg，0.17mmol) was added and the mixture was degassed and backfilled with nitrogen gas (4×) again. The mixture was heated at 80 °C for 12 hours, and after this time the reaction was allowed to cool to room temperature. Water (20 mL) was added and the resulting mixture was extracted with ethyl acetate (3 × 100 mL). The combined organic layers were washed with 5% aqueous lithium chloride (3 × 100 mL), washed with saturated aqueous sodium chloride (300 mL), dried over magnesium sulfate, filtered and concentrated to dryness.

The residue obtained was purified via flash SiO_2 chromatography (34 g silica gel, gradient of hexanes to 25% ethyl acetate/75% hexanes) to give 1-(2-((*tert*-butyldimethylsilyl)oxy)-2-methylpropyl)-2-(ethoxymethyl)-5-(*p*-tolyl)-1*H*-imidazole**(59)**(20 mg, 5%) as an orange oil. ^{1}H NMR (400 MHz, $CDCl_3$) δ 7.28-7.19 (m, 4H), 6.97 (s, 1H), 4.79 (s, 2H), 4.22 (s, 2H), 3.58 (q, *J* = 7.1 Hz, 2H), 2.40 (s, 3H), 1.25 (t, *J* = 7.0 Hz, 3H), 0.94 (s, 6H), 0.83-0.77 (m, 9H); ^{13}C NMR (101 MHz, $CDCl_3$) δ 147.28, 137.48, 134.47, 129.46, 128.80, 128.67, 127.03, 74.40, 65.83, 65.73, 54.91, 28.56, 25.95, 21.22, 18.02, 15.13, -2.05.

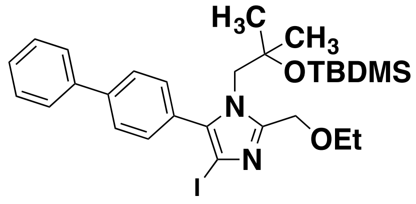

5-([1,1'-biphenyl]-4-yl)-1-(2-((*tert*-butyldimethylsilyl)oxy)-2-methylpropyl)-2-(ethoxymethyl)-4-iodo-1*H*-imidazole (60)

To a solution of 5-([1,1'-biphenyl]-4-yl)-1-(2-((*tert*-butyldimethylsilyl)oxy)-2-methylpropyl)-2-(ethoxymethyl)-1*H*-imidazole(58)(56mg, 0.12 mmol) in *N*,*N*-dimethylformamide (5 mL) at 60 °C was added a solution of *N*-iodosuccinimide (30 mg, 0.13 mmol) in *N*,*N*-dimethylformamide (1 mL) and the solution was allowed to stir 60 °C for 12 hours. After this time, water (10 mL) was added, solid sodium thiosulfate pentahydrate was added until no further color change was observed, and the resulting mixture was extracted with ethyl acetate (3 × 10 mL). The combined organic layers were washed with 5% aqueous lithium chloride (3 × 30 mL), washed with saturated aqueous sodium chloride (30 mL), dried over magnesium sulfate, filtered and concentrated to dryness. The residue obtained was purified via flash SiO_2 chromatography (10 g silica gel,

gradient of hexanes to 20% ethyl acetate/80% hexanes) to give 5-([1,1'-biphenyl]-4-yl)-1-(2-((*tert*-butyldimethylsilyl)oxy)-2-methylpropyl)-2-(ethoxymethyl)-4-iodo-1*H*-imidazole**(60)** (32 mg, 45%) as a yellow oil; ^{1}H NMR (400 MHz, $CDCl_3$) δ 7.67 (dd, *J* = 15.5, 7.8 Hz, 4H), 7.51-7.34 (m, 5H), 4.75 (s, 2H), 4.19 (s, 2H), 3.58 (q, *J* = 7.0 Hz, 2H), 0.93 (s, 6H), 0.79 (s, 9H), -0.01 (s, 6H); ^{13}C NMR (101 MHz, $CDCl_3$) δ 149.23, 141.09, 140.17, 135.72, 133.22, 130.99, 129.51, 128.88, 127.71, 127.26, 127.06, 74.25, 65.96, 65.63, 55.77, 28.59, 25.97, 17.91, 15.13, -2.01.

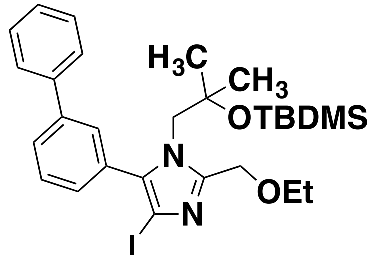

5-([1,1'-biphenyl]-3-yl)-1-(2-((*tert*-butyldimethylsilyl)oxy)-2-methylpropyl)-2-(ethoxymethyl)-4-iodo-1*H*-imidazole (61)

To a solution of 5-([1,1'-biphenyl]-3-yl)-1-(2-((*tert*-butyldimethylsilyl)oxy)-2-methylpropyl)-2-(ethoxymethyl)-1*H*-imidazole(59)(48mg, 0.10 mmol) in *N*,*N*-dimethylformamide (5 mL) at 60 °C was added a solution of *N*-iodosuccinimide (28 mg, 0.12 mmol) in *N*,*N*-dimethylformamide (1 mL) and the solution was allowed to stir 60 °C for 12 hours. After this time, water (10 mL) was added, solid sodium thiosulfate pentahydrate was added until no further color change was observed, and the resulting mixture was extracted with ethyl acetate (3 × 10 mL). The combined organic layers were washed with 5% aqueous lithium chloride (3 × 30 mL), washed with saturated aqueous sodium chloride (30 mL), dried over magnesium sulfate, filtered and concentrated to

dryness. The residue obtained was purified via flash SiO_2 chromatography (10 g silica gel, gradient of hexanes to 20% ethyl acetate/80% hexanes) to give 5-([1,1'-biphenyl]-3-yl)-1-(2-((*tert*-butyldimethylsilyl)oxy)-2-methylpropyl)-2-(ethoxymethyl)-4-iodo-1*H*-imidazole**(61)** (13 mg, 21%) as a yellow oil; ^{1}H NMR (400 MHz, $CDCl_3$) δ 7.67-7.58 (m, 4H), 7.50 (dt, J = 19.6, 7.7 Hz, 3H), 7.41-7.30 (m, 2H), 4.75 (s, 2H), 4.19 (s, 2H), 3.58 (q, J = 7.0 Hz, 2H), 1.25 (d, J = 7.0 Hz, 3H), 0.92 (s, 6H), 0.78 (s, 9H), -0.02 (s, 6H); ^{13}C NMR (101 MHz, $CDCl_3$) δ 149.20, 141.63, 140.42, 135.96, 131.09, 129.47, 129.27, 129.11, 127.69, 127.17, 84.76, 74.26, 66.00, 65.63, 55.83, 28.59, 25.96, 18.03, 15.13, -2.02.

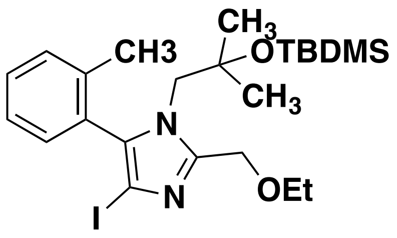

1-(2-((*tert*-butyldimethylsilyl)oxy)-2-methylpropyl)-2-(ethoxymethyl)-4-iodo-5-(*o*-tolyl)-1*H*-imidazole (62)

To a solution of 1-(2-((*tert*-butyldimethylsilyl)oxy)-2-methylpropyl)-2-(ethoxymethyl)-5-(*o*-tolyl)-1*H*-imidazole(62)(186mg, 0.46 mmol) in *N*,*N*-dimethylformamide (5 mL) at 60 °C was added a solution of *N*-iodosuccinimide (125 mg, 0.56 mmol) in *N*,*N*-dimethylformamide (1 mL) and the solution was allowed to stir 60 °C for 12 hours. After this time, water (10 mL) was added, solid sodium thiosulfate pentahydrate was added until no further color change was observed, and the resulting mixture was extracted with ethyl acetate (3 x 10 mL). The combined organic layers were washed with 5% aqueous lithium chloride (3 x 30 mL), washed with saturated aqueous sodium chloride (30 mL),

dried over magnesium sulfate, filtered and concentrated to dryness. The residue obtained was purified via flash SiO_2 chromatography (10 g silica gel, gradient of hexanes to 16.7% ethyl acetate/83.3% hexanes) to give 1-(2-((*tert*-butyldimethylsilyl)oxy)-2-methylpropyl)-2-(ethoxymethyl)-4-iodo-5-(*o*-tolyl)-1*H*-imidazole**(62)**(200 mg, 82%) as a yellow oil; ^{1}H NMR (400 MHz, Chloroform-*d*) δ 7.38 – 7.27 (m, 3H), 7.25 – 7.14 (m, 1H), 4.85 – 4.67 (m, 2H), 4.11 (d, *J* = 14.5 Hz, 1H), 3.78 (d, *J* = 14.5 Hz, 1H), 3.54 (dqt, *J* = 9.5, 7.0, 4.1 Hz, 2H), 2.17 (s, 3H), 1.21 (t, *J* = 7.0 Hz, 3H), 0.96 (s, 3H), 0.93 (s, 3H), 0.79 (s, 9H), 0.00(s, 6H).

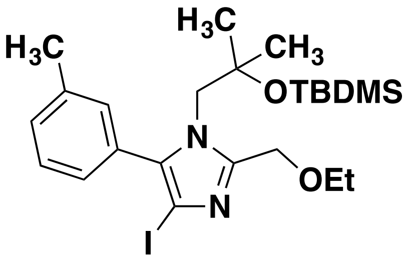

1-(2-((*tert*-butyldimethylsilyl)oxy)-2-methylpropyl)-2-(ethoxymethyl)-4-iodo-5-(*m*-tolyl)-1*H*-imidazole (63)

To a solution of 1-(2-((*tert*-butyldimethylsilyl)oxy)-2-methylpropyl)-2-(ethoxymethyl)-5-(*m*-tolyl)-1*H*-imidazole(63)(127mg, 0.32 mmol) in *N*,*N*-dimethylformamide (5 mL) at 60 °C was added a solution of *N*-iodosuccinimide (127 mg, 0.56 mmol) in *N*,*N*-dimethylformamide (1 mL) and the solution was allowed to stir 60 °C for 12 hours. After this time, water (10 mL) was added, solid sodium thiosulfate pentahydrate was added until no further color change was observed, and the resulting mixture was extracted with ethyl acetate (3 × 10 mL). The combined organic layers were washed with 5% aqueous lithium chloride (3 × 30 mL), washed with saturated aqueous sodium chloride (30 mL), dried over magnesium sulfate, filtered and concentrated to dryness. The residue obtained

was purified via flash SiO_2 chromatography (10 g silica gel, gradient of hexanes to 20% ethyl acetate/80% hexanes) to give 1-(2-((*tert*-butyldimethylsilyl)oxy)-2-methylpropyl)-2-(ethoxymethyl)-4-iodo-5-(*m*-tolyl)-1*H*-imidazole**(63)**(90 mg, 71%) as colorless oil; ^{1}H NMR (400 MHz, Chloroform-*d*) δ 7.32 (t, *J* = 7.8 Hz, 1H), 7.24 – 7.12 (m, 3H), 4.73 (s, 2H), 4.12 (d, *J* = 14.3 Hz, 2H), 3.55 (q, *J* = 7.0 Hz, 2H), 2.40 (s, 3H), 1.23 (dt, *J* = 12.1, 7.2 Hz, 3H), 0.89 (s, 6H), 0.78 (s, 9H), -0.02 (s, 6H). ^{13}C NMR (101 MHz, $CDCl_3$) δ 148.93, 138.32, 136.16, 131.11, 130.42, 129.24, 128.52, 127.82, 84,25, 74.23, 65.92, 65.57, 55.67, 28.55, 25.96, 21.43, 18.03, 15.12, -2.03.

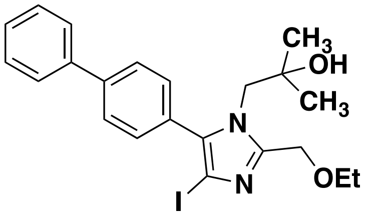

1-(5-([1,1'-biphenyl]-4-yl)-2-(ethoxymethyl)-4-iodo-1*H*-imidazol-1-yl)-2-methylpropan-2-ol (3a)

To a solution of 5-([1,1'-biphenyl]-4-yl)-1-(2-((*tert*-butyldimethylsilyl)oxy)-2-methylpropyl)-2-(ethoxymethyl)-4-iodo-1*H*-imidazole(65) (0.027 g, 0.05 mmol) in tetrahydrofuran (5 mL) was added tetrabutylammonium fluoride (0.14 mL, 0.14 mmol, 1 M solution in tetrahydrofuran) and the solution was allowed to stir at room temperature for 12hours. After this time, saturated aqueous ammonium chloride (10 mL) was added and the mixture was extracted with ethyl acetate (3 × 5 mL). The combined organic layers were washed with saturated aqueous sodium chloride (10 mL), dried over magnesium sulfate, filtered and concentrated to dryness. The residue obtained was purified via flash SiO_2 chromatography (10 g silica gel, gradient of dichloromethane to 4% methanol/96% dichloromethane) to give1-(5-([1,1'-biphenyl]-4-yl)-2-(ethoxymethyl)-4-

iodo-1*H*-imidazol-1-yl)-2-methylpropan-2-ol**(3a)** (0.021 g, 99%) as a yellow oil; ^{1}H NMR (400 MHz, $CDCl_3$) δ 7.71 (d, *J* = 8.3 Hz, 2H), 7.66 (d, *J* = 7.3 Hz, 2H), 7.47 (d, *J* = 15.1 Hz, 2H), 7.39 (d, *J* = 7.8 Hz, 3H), 4.68 (s, 2H), 4.17 (s, 2H), 3.68 (q, *J* = 7.0 Hz, 2H), 3.61 (s, 1H), 1.26 (d, *J* = 7.0 Hz, 3H), 0.93 (s, 6H); ^{13}C NMR (101 MHz, $CDCl_3$) δ 147.61, 141.54, 140.02, 136.64, 131.26, 128.91, 128.82, 127.82, 127.36, 127.08, 84.98, 70.45, 66.31, 64.61, 55.59, 27.74, 14.90. MS (ESI^+), calcd $C_{22}H_{25}IN_2O_2$ (M+H) = 477.1039, found = 477.1046.

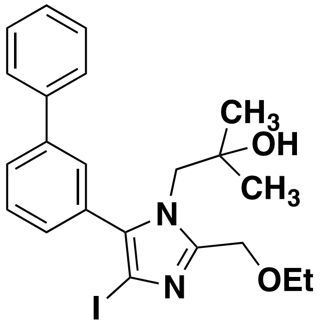

1-(5-([1,1'-biphenyl]-3-yl)-2-(ethoxymethyl)-4-iodo-1*H*-imidazol-1-yl)-2-methylpropan-2-ol (3b)

To a solution of 5-([1,1'-biphenyl]-3-yl)-1-(2-((*tert*-butyldimethylsilyl)oxy)-2-methylpropyl)-2-(ethoxymethyl)-4-iodo-1*H*-imidazole(66)(0.013 g, 0.02 mmol) in tetrahydrofuran (5 mL) was added tetrabutylammonium fluoride (0.066 mL, 0.066 mmol, 1 M solution in tetrahydrofuran) and the solution was allowed to stir at room temperature for 12hours. After this time, saturated aqueous ammonium chloride (10 mL) was added and the mixture was extracted with ethyl acetate (3 × 5 mL). The combined organic layers were washed with saturated aqueous sodium chloride (10 mL), dried over magnesium sulfate, filtered and concentrated to dryness. The residue obtained was purified via flash SiO_2 chromatography (10 g silica gel, gradient of dichloromethane to 4%

methanol/96% dichloromethane) to give1-(5-([1,1'-biphenyl]-3-yl)-2-(ethoxymethyl)-4-iodo-1*H*-imidazol-1-yl)-2-methylpropan-2-ol**(3b)**(0.010 g, 95%) as a yellow oil; ^{1}H NMR (400 MHz, $CDCl_3$) δ 7.71-7.54 (m, 5H), 7.50 (t, *J* = 7.5 Hz, 2H), 7.41 (t, *J* = 7.2 Hz, 1H), 7.31 (d, *J* = 7.6 Hz, 1H), 4.71 (s, 2H), 4.20 (s, 2H), 3.71 (q, *J* = 7.0 Hz, 2H), 3.61 (s, 1H), 1.29 (s, 3H), 0.95 (s, 6H);^{13}C NMR (101 MHz, $CDCl_3$) δ 147.58, 141.72, 140.23, 136.90, 130.44, 129.71, 129.51, 129.22, 128.95, 127.79, 127.63, 127.15, 85.30, 70.45, 66.33, 64.56, 55.63, 27.74, 14.89. MS (ESI^+), calcd $C_{22}H_{25}IN_2O_2$ (M+H) = 477.1039, found = 477.1046.

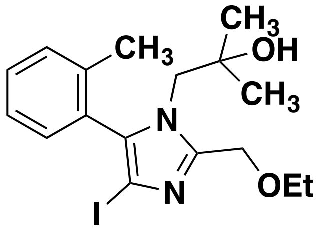

1-(2-(ethoxymethyl)-4-iodo-5-(*o*-tolyl)-1*H*-imidazol-1-yl)-2-methylpropan-2-ol (3c)

To a solution of 1-(2-((*tert*-butyldimethylsilyl)oxy)-2-methylpropyl)-2-(ethoxymethyl)-4-iodo-5-(*o*-tolyl)-1*H*-imidazole(69) (0.10 g, 0.19 mmol) in tetrahydrofuran (5 mL) was added tetrabutylammonium fluoride (0.28 mL, 0.28 mmol, 1 M solution in tetrahydrofuran) and the solution was allowed to stir at room temperature for 12 hours. After this time, saturated aqueous ammonium chloride (10 mL) was added and the mixture was extracted with ethyl acetate (3 × 5 mL). The combined organic layers were washed with saturated aqueous sodium chloride (10 mL), dried over magnesium sulfate, filtered and concentrated to dryness. The residue obtained was purified via flash SiO_2 chromatography (10 g silica gel, gradient of dichloromethane to 4% methanol/96% dichloromethane) to give 1-(2-(ethoxymethyl)-4-iodo-5-(*o*-tolyl)-1*H*-imidazol-1-yl)-2-

methylpropan-2-ol **(3c)**(0.021 g, 99%) as a yellow solid; ^{1}H NMR (400 MHz, Chloroform-*d*) δ 7.39 – 7.27 (m, 3H), 7.16 (dd, *J* = 7.6, 1.4 Hz, 1H), 4.70 – 4.62 (m, 2H), 4.07 (d, *J* = 14.9 Hz, 1H), 3.88 (s, 1H), 3.76 – 3.58 (m, 3H), 2.12 (s, 3H), 1.23 (t, *J* = 7.0 Hz, 3H), 0.94 (d, *J* = 1.6 Hz, 6H). MS (ESI^{+}), calcd $C_{17}H_{24}IN_2O_2$ (M+H) = 415.0882, found = 415.0889.

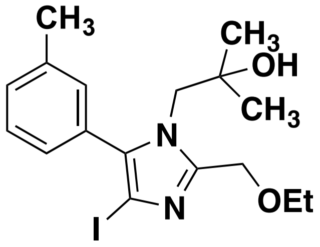

1-(2-(ethoxymethyl)-4-iodo-5-(*m*-tolyl)-1*H*-imidazol-1-yl)-2-methylpropan-2-ol (3d)

To a solution of 1-(2-((*tert*-butyldimethylsilyl)oxy)-2-methylpropyl)-2-(ethoxymethyl)-4-iodo-5-(*m*-tolyl)-1*H*-imidazole(70)(0.090 g, 0.17 mmol) in tetrahydrofuran (5 mL) was added tetrabutylammonium fluoride (0.60mL, 0.60 mmol, 1 M solution in tetrahydrofuran) and the solution was allowed to stir at room temperature for 12hours. After this time, saturated aqueous ammonium chloride (10 mL) was added and the mixture was extracted with ethyl acetate (3 × 5 mL). The combined organic layers were washed with saturated aqueous sodium chloride (10 mL), dried over magnesium sulfate, filtered and concentrated to dryness. The residue obtained was purified via flash SiO_2 chromatography (10 g silica gel, gradient of dichloromethane to 4% methanol/96% dichloromethane) to give1-(2-(ethoxymethyl)-4-iodo-5-(*m*-tolyl)-1*H*-imidazol-1-yl)-2-methylpropan-2-ol**(3d)** (0.050 g, 71%) as a colorless oil; 1H NMR (400 MHz, Chloroform-*d*) δ 7.38 (t, *J* = 7.7 Hz, 1H), 7.26 (d, *J* = 7.7 Hz, 1H), 7.13 (d, *J* = 7.2 Hz, 2H), 4.68 (s, 2H), 4.14 (s, 2H), 3.69 (q, *J* = 7.0 Hz, 2H), 3.64 (s, 1H), 2.44 (s, 3H), 1.27

(d, J = 7.1 Hz, 3H), 0.93 (s, 6H). ^{13}C NMR (101 MHz, $CDCl_3$) δ 147.29, 138.48, 137.11, 131.42, 129.82, 129.73, 128.63, 128.00, 84.67, 70.39, 66.24, 64.56, 55.59, 27.67, 21.43, 14.87. MS (ESI$^+$), calcd $C_{17}H_{24}IN_2O_2$ (M+H) = 415.0882, found = 415.0889.

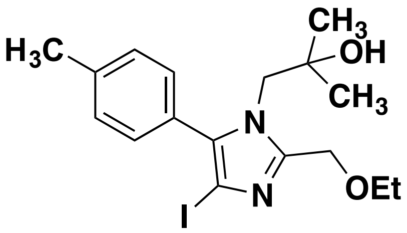

1-(2-(ethoxymethyl)-4-iodo-5-(*p*-tolyl)-1*H*-imidazol-1-yl)-2-methylpropan-2-ol (3e)

To a solution of 1-(2-((*tert*-butyldimethylsilyl)oxy)-2-methylpropyl)-2-(ethoxymethyl)-5-(*p*-tolyl)-1*H*-imidazole(**59**)(56mg, 0.12 mmol) in *N*,*N*-dimethylformamide (5 mL) at 60 °C was added a solution of *N*-iodosuccinimide (30 mg, 0.13 mmol) in *N*,*N*-dimethylformamide (1 mL) and the solution was allowed to stir 60 °C for 12 hours. After this time, water (10 mL) was added, solid sodium thiosulfate pentahydrate was added until no further color change was observed, and the resulting mixture was extracted with ethyl acetate (3 × 10 mL). The combined organic layers were washed with 5% aqueous lithium chloride (3 × 30 mL), washed with saturated aqueous sodium chloride (30 mL), dried over magnesium sulfate, filtered and concentrated to dryness to obtain the crude pruduct 1-(2-((*tert*-butyldimethylsilyl)oxy)-2-methylpropyl)-2-(ethoxymethyl)-4-iodo-5-(*p*-tolyl)-1*H*-imidazole(**64**) without further purification. Then tetrahydrofuran (5ml) and tetrabutylammonium fluoride (0.23 mL, 0.23 mmol, 1 M solution in tetrahydrofuran) were added seperately and the solution was allowed to stir at room temperature for 17 hours. After this time, saturated aqueous ammonium chloride (10 mL) was added and the mixture was extracted with ethyl acetate (3 × 5 mL). The combined organic layers were

washed with saturated aqueous sodium chloride (10 mL), dried over magnesium sulfate, filtered and concentrated to dryness. The residue obtained was purified via flash SiO_2 chromatography (10 g silica gel, gradient of hexanes to 25% ethyl acetate/75% hexanes) to give 1-(2-(ethoxymethyl)-4-iodo-5-(*p*-tolyl)-1*H*-imidazol-1-yl)-2-methylpropan-2-ol**(3e)** (15 mg, 72%) as a yellow solid; ^1H NMR (400 MHz, $CDCl_3$) δ 7.31 (s, 2H), 7.22 (d, *J* = 8.1 Hz, 2H), 4.69 (s, 2H), 4.14 (s, 2H), 3.69 (q, *J* = 7.0 Hz, 2H), 2.44 (s, 3H), 1.26 (t, *J* = 7.0 Hz, 3H), 0.93 (s, 6H); ^{13}C NMR (101 MHz, $CDCl_3$) δ 147.22, 138.97, 137.04, 130.76, 129.53, 126.87, 84.33, 70.38, 66.25, 64.54, 55.56, 27.66, 21.42, 14.88. MS (ESI^+), calcd $C_{17}H_{24}IN_2O_2$ (M+H) = 415.0882, found =415.0889.
